# Transient Fcho1/2⋅Eps15/R⋅AP-2 Nanoclusters Prime the AP-2 Clathrin Adaptor for Cargo Binding

**DOI:** 10.1016/j.devcel.2016.05.003

**Published:** 2016-06-06

**Authors:** Li Ma, Perunthottathu K. Umasankar, Antoni G. Wrobel, Anastasia Lymar, Airlie J. McCoy, Sachin S. Holkar, Anupma Jha, Tirthadipa Pradhan-Sundd, Simon C. Watkins, David J. Owen, Linton M. Traub

**Affiliations:** 1 Department of Cell Biology, University of Pittsburgh School of Medicine, 3500 Terrace Street, S312 BST, Pittsburgh, PA 15261, USA; 2 Cambridge Institute for Medical Research, University of Cambridge, Cambridge CB2 0XY, UK

## Abstract

Clathrin-coated vesicles form by rapid assembly of discrete coat constituents into a cargo-sorting lattice. How the sequential phases of coat construction are choreographed is unclear, but transient protein-protein interactions mediated by short interaction motifs are pivotal. We show that arrayed Asp-Pro-Phe (DPF) motifs within the early-arriving endocytic pioneers Eps15/R are differentially decoded by other endocytic pioneers Fcho1/2 and AP-2. The structure of an Eps15/R⋅Fcho1 μ-homology domain complex reveals a spacing-dependent DPF triad, bound in a mechanistically distinct way from the mode of single DPF binding to AP-2. Using cells lacking FCHO1/2 and with Eps15 sequestered from the plasma membrane, we establish that without these two endocytic pioneers, AP-2 assemblies are fleeting and endocytosis stalls. Thus, distinct DPF-based codes within the unstructured Eps15/R C terminus direct the assembly of temporary Fcho1/2⋅Eps15/R⋅AP-2 ternary complexes to facilitate conformational activation of AP-2 by the Fcho1/2 interdomain linker to promote AP-2 cargo engagement.

## Highlights

• The endocytic pioneer protein Eps15 engages AP-2 and Fcho1/2 noncompetitively
• Structural analysis shows arrayed DPF motif triad in Eps15 for Fcho1/2 μHD binding
• DPF-based codes direct transient Fcho1/2⋅Eps15/R⋅AP-2 ternary complex formation
• In ternary complex, Fcho1 interdomain linker primes AP-2 for cargo capture

## Graphical Abstract

**Figure:**
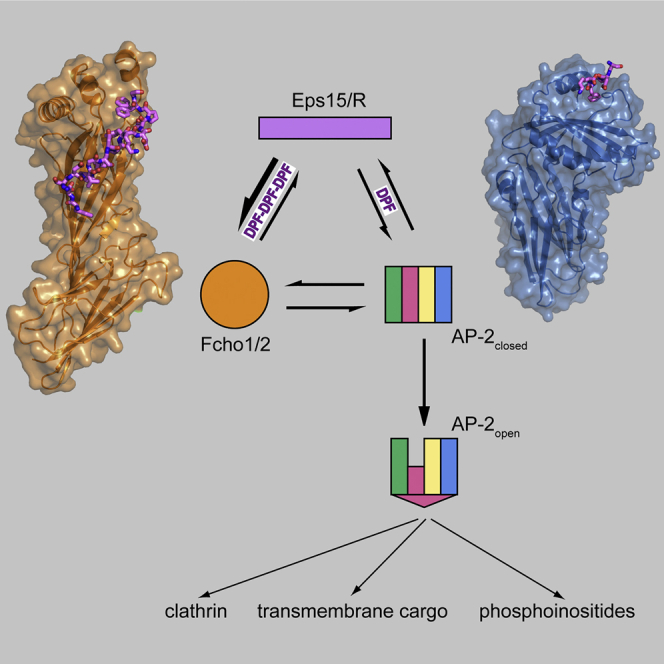

## Teaser

How clathrin-coated vesicles initiate at the plasma membrane is uncertain. Ma et al. show through structural and biochemical analyses that a three-way interaction between early-arriving endocytic factors Eps15/R and Fcho1/2, and the adaptor AP-2, promotes membrane deposition of AP-2 in open conformation for sustained clathrin-coat assembly and cargo loading.

## Introduction

The minute-to-minute protein composition of the eukaryotic plasma membrane is managed by clathrin-mediated endocytosis (CME) (Bitsikas et al., 2014). Rapid internalization occurs at specialized zones called clathrin-coated structures (CCSs), where signal-dependent cargo packaging is handled by the heterotetrameric AP-2 adaptor complex with an attendant set of clathrin-associated sorting proteins (CLASPs) (Reider and Wendland, 2011, Traub, 2009). As new CCSs initiate, a subset of coat machinery congregates first, priming the plasma membrane for effective transport vesicle production. These early-arriving—pioneer—proteins include AP-2 and clathrin, the principal structural units of surface-derived CCSs, as well as Eps15/R (epidermal growth factor pathway substrate 15/Eps15 related) and Fcho1/2 (Fer/CIP4 homology domain only protein 1 or 2) (Taylor et al., 2011). The pioneer module of endocytic proteins is densely interconnected; deposition and residence of these factors depends on physical connections with the organizing plasma membrane lipid phosphatidylinositol 4,5-bisphosphate (PtdIns(4,5)P_2_) (Antonescu et al., 2011, Schifferer et al., 2015, Zoncu et al., 2007) and on direct connections among one another, typically mediated by short linear peptide interaction motifs (McMahon and Boucrot, 2011, Traub, 2009).

The lack of a tightly coupled energy input suggests that arrival of AP-2, pioneers, and CLASPs at the plasma membrane is a stochastic phenomenon (Ehrlich et al., 2004, Godlee and Kaksonen, 2013, Larson et al., 2014), in part underpinning the variability in the precise location of CCS initiation. Given the multiplicity of possible protein-protein interactions at nucleation, it is uncertain how timely forward progression is achieved, and how promiscuous or off-pathway associations are avoided. This issue is critical, as the autoinhibited soluble AP-2 adaptor requires allosteric activation at CCSs (Jackson et al., 2010, Kelly et al., 2014).

Fcho1 and Fcho2 likely play a key role in local AP-2 restructuring, as they are among the earliest proteins to mark a nascent CCS (Henne et al., 2010, Taylor et al., 2011) and affect AP-2 conformation directly (Hollopeter et al., 2014, Umasankar et al., 2014). The two paralogs contain an N-terminal crescent-shaped, membrane-binding EFC (extended FCH) domain also called an F-BAR domain (Henne et al., 2007). The leading EFC domain is followed by an evolutionarily least conserved and intrinsically disordered segment of ∼200 residues that associates with the AP-2 adaptor physically (Hollopeter et al., 2014, Umasankar et al., 2012, Umasankar et al., 2014). But what makes Fchos unusual is that the C-terminal SH3 domain typical of most EFC-domain proteins is replaced by a μ-homology domain (μHD), distantly related in primary sequence to cargo-selective μ subunits of the heterotetrameric clathrin adaptors such as AP-2 (Reider et al., 2009). This combination of an EFC domain with a μHD is exclusive and phylogenetically conserved in opisthokonts. The thus misnamed Fcho1 and Fcho2 share overall domain architecture (Katoh, 2004), while the neuron-enriched Sgip1 (SH3-domain GRB2-like [endophilin] interacting protein 1) protein has a μHD but lacks the folded EFC domain (Uezu et al., 2007). The μHD of all three (designated the muniscins) binds directly to the pioneer protein Eps15 (Henne et al., 2010, Reider et al., 2009, Uezu et al., 2007, Umasankar et al., 2012). To better comprehend the functional consequence of inaugural protein encounters at a clathrin assembly zone, we report three sequential stages of inquiry. First, we delineate the minimal sequence tract in Eps15/R necessary to bind the μHD. Next, a 2.4-Å-resolution structure of these two interaction partners provides an atomic-level description of the binding mechanism. Lastly, delineating the structurally distinct manners in which the μHD and AP-2 appendages bind to Asp-Pro-Phe (DPF) triplets within Eps15/R allows us to formulate and test in vivo a concept for pioneer-coordinated activation of AP-2 at plasma membrane bud sites by Eps15 bringing conformationally closed AP-2 into proximity with the Fcho interdomain linker.

## Results

### A μHD Interaction Network

Fcho1 and Fcho2 have numerous binding partners (Henne et al., 2010, Mulkearns and Cooper, 2012, Reider et al., 2009, Uezu et al., 2007, Umasankar et al., 2012). The chief interaction surface is the globular C-terminal μHD. This is seen as apparent loss of coincidence of an expressed GFP-FCHO1 lacking the μHD with endogenous AP-2- and HRB-positive surface puncta in transfected HeLa cells (Figures S1A and S1B). Conversely, a tandem dimer red fluorescent protein (tdRFP)-tagged FCHO1 μHD alone (residues 609–889) localizes to AP-2-marked CCSs when transiently expressed (Figures S1C and S1D). The FCHO1 μHD binds directly to HRB as well as to EPS15, EPS15R, intersectin 1, DAB2, and CALM (Figure S1E) (Henne et al., 2010, Reider et al., 2009, Umasankar et al., 2012). Amino acid divergence between the FCHO1 and structurally related FCHO2 (53% identity) and Sgip1 (49% identity) μHDs is paralleled by differences in partner protein selectivity; FCHO2 and Sgip1 contact a subset of FCHO1 partners (Mulkearns and Cooper, 2012, Uezu et al., 2011, Umasankar et al., 2012) including EPS15 (Figure S1E), but only the FCHO1 μHD binds to HRB and CALM in our assays.

**Figure 1:**
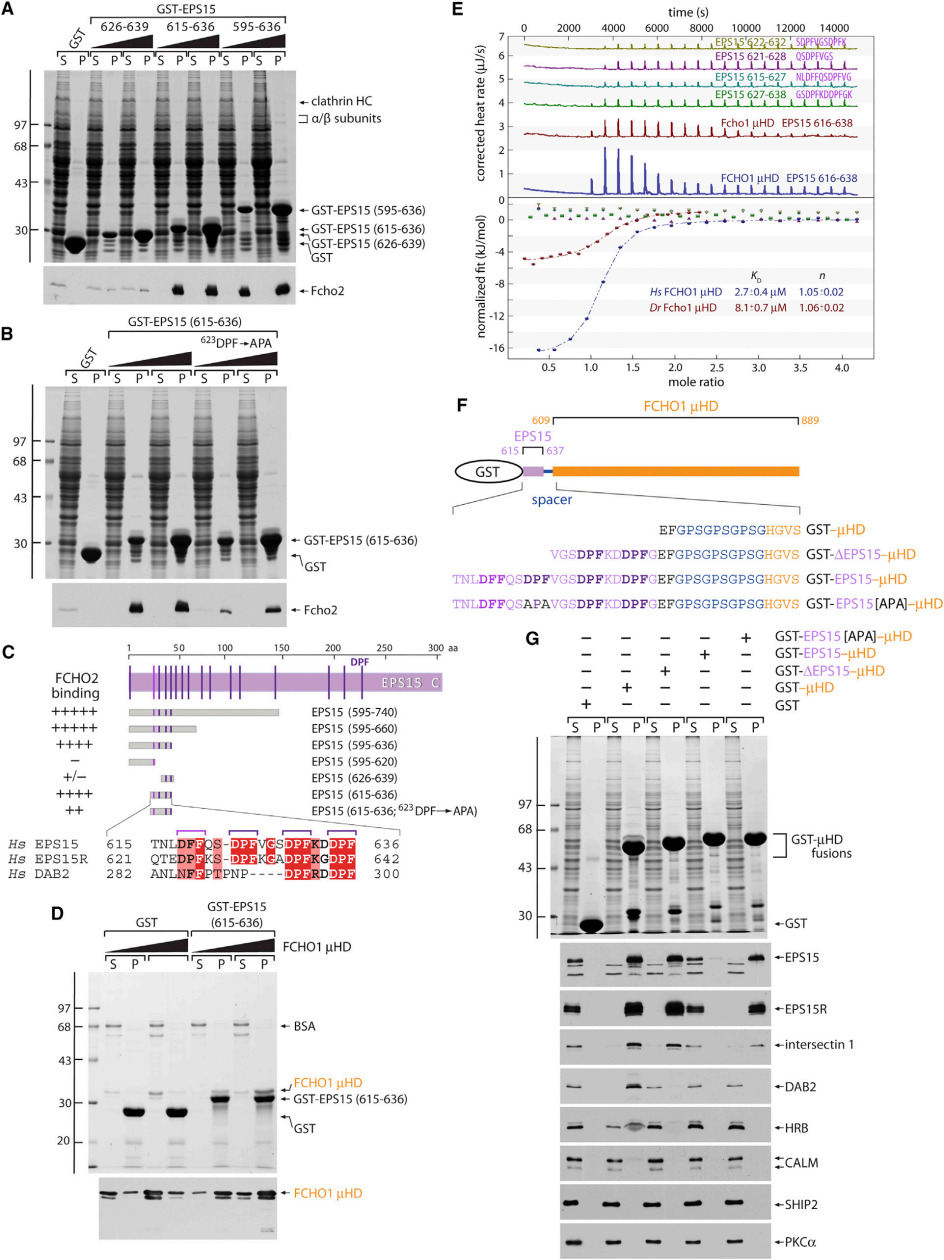
A Minimal EPS15 Interaction Motif (A and B) GST pull-down assays with rat brain cytosol and 250 μg of GST and either 50 or 250 μg of the indicated GST-EPS15 fusion proteins. SDS-PAGE-separated supernatant (S, 2%) and pellet (P, 10%) fractions were stained or immunoblotted with antibodies against Fcho2. (C) Location of DPF triplets in the EPS15 C-terminal domain, with the relative location of the various fragments tested below. Alignment of *Homo sapiens* (*Hs*) EPS15, EPS15R, and DAB2 sequences corresponding to the minimal motif, boxed with red for identity and pink for similarity. (D) GST pull-down assay with purified FCHO1 μHD and ∼50 μg of either GST or GST-μHD fusion protein in the presence of carrier BSA. SDS-PAGE-separated supernatant and pellet fractions were stained or immunoblotted with an anti-FCHO1 monoclonal antibody. (E) Representative ITC experiments with the *Danio rerio* (*Dr*) Fcho1 μHD and EPS15 616–638 peptide or the *Hs* FCHO1 μHD titrated with the 616–638 or indicated EPS15 peptides. *K*_D_, reaction stoichiometry (*n*), and errors were calculated from a minimum of four runs. (F) Cartoon representation of GST-EPS15-FCHO1 μHD chimeras, with the specific EPS15 sequence for each variant preceding the GlyProSer spacer shown below. (G) GST pull-down assay using HeLa cell lysate and 250 μg of either GST or GST-μHD fusion proteins as indicated. Separated supernatant and pellet fractions were stained or replicates immunoblotted with the indicated antibodies as in (A).

The association of muniscin μHDs with EPS15 requires the unstructured C terminus (residues 595–896) of the protein (Cupers et al., 1997), rich in tripeptide DPF repeats. The AP-2 α appendage binds to this general segment of EPS15 as well, but the mode of engagement is clearly different; AP-2 binding increases linearly with additional EPS15 DPFs (Benmerah et al., 1996, Iannolo et al., 1997) but Fcho2 binding does not (Figure S1F). When EPS15 (595–896) is immobilized on excess glutathione S-transferase (GST)-AP-2 α appendage, the FCHO1 μHD can simultaneously also bind EPS15 (Figures S1G and S1H). This suggests non-overlapping DPF targets, as ternary complexes can form by synchronous binding to different regions of the EPS15 C terminus. Further truncation/deletion analysis defines a minimum region (^615^TNLDFFQSDPFVGSDPFKDDPF) of EPS15 sufficient for engaging Fcho1/2 (Figures 1A–1C). FCHO1 μHD binding to EPS15 (615–636) is dose dependent and direct (Figure 1D). This EPS15 tract contains three closely spaced DPF repeats (and a leading DFF triplet) highly conserved in chordates, and is additionally present in EPS15R (Figure 1C). The distal region (^629^DPFKDDPF) of the EPS15 μHD-binding element is nearly identical to a small portion of the CLASP DAB2 (Figures S1 and 1C) that is needed for a direct association with Fcho2 (Henne et al., 2010, Mulkearns and Cooper, 2012, Umasankar et al., 2012). Yet EPS15 and EPS15R bind the μHD with higher apparent affinity than DAB2 (see Figure 3F), suggesting the participation of the first of the three tandem DPFs, which is not found in DAB2 (Figure 1C) (Mulkearns and Cooper, 2012).

### Dissecting the DPF-Based EPS15 μHD-Binding Sequence

The role of the proximal half of the mapped DPF tract on μHD engagement is highlighted by the substantially reduced apparent affinity of cytosolic Fcho2 for the GST-EPS15 fusion protein when either residues 615–625 (TNLDFFQSDPF) are removed (Figure 1A) or a ^623^DPF→APA mutation is introduced into the longer 615–636 form (Figure 1B). No binding of four short peptides encompassing only one or two DPFs between EPS15 residues 616 and 632 is detectable by isothermal titration calorimetry (ITC) (Figure 1E). In agreement, artificially fusing portions of EPS15 directly to the N terminus of the FCHO1 μHD (Figure 1F) affects free partner binding, presumably through intramolecular competition. Fusing EPS15 residues 626–637 (ΔEPS15; containing only DPFX_2_DPF), while not interfering effectively with EPS15/R or intersectin 1 binding, prohibits the engagement of the weaker-binding proteins, including DAB2 (Figure 1G). By contrast, linking EPS15 residues 615–637 (with DFF and DPFX_3_DPFX_2_DPF) essentially eliminates all partner binding. A ^623^DPF→APA substitution in the full (residues 615–637) tandem fusion diminishes EPS15/R and intersectin binding more efficiently than does the short fusion (residues 626–637). This again implicates the proximal section of the EPS15 sequence in higher-affinity binding. Neither HRB nor CALM have related DPFX_2_DPF motifs within the unstructured polypeptide regions, but are Phe rich, suggesting that the weaker affinity of these FCHO1 μHD partners likely reflects use of variant but related interaction motif(s).

**Figure 2:**
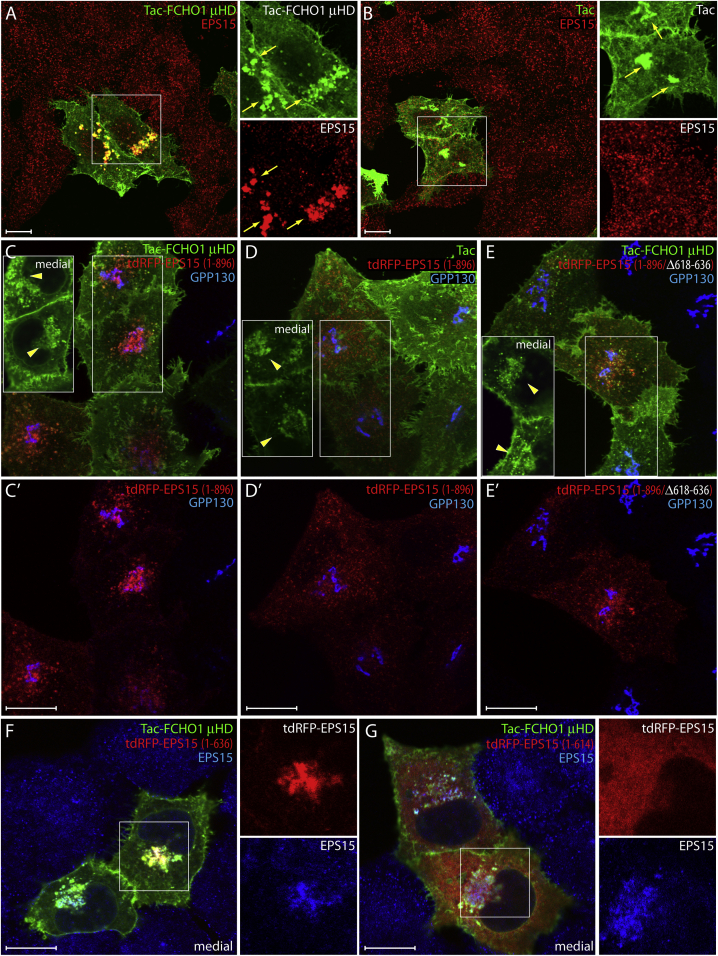
The EPS15 μHD Interaction Motif In Vivo (A and B) Representative single confocal optical sections of basal surface of HeLa SS6 cells transfected with either Tac-FCHO1 μHD (A) or Tac (B) as indicated. Fixed cells were stained with anti-Tac or anti-EPS15 antibodies. Enlarged color-separated regions corresponding to the boxed areas are shown and Golgi-localized Tac and EPS15 (arrows) are indicated. Scale bar, 10 μm. (C–E′) Typical basal confocal sections of fixed HeLa cells double transfected with the indicated Tac and tdRFP-EPS15 plasmids and stained with antibodies against Tac and GPP130 as indicated (C′–E′). Corresponding medial optical sections of the boxed regions are shown (C–E) indicating the Golgi pool (arrowheads) of Tac protein. Scale bar, 10 μm. (F and G) Representative medial confocal optical sections of HeLa cells transiently transfected with Tac-FCHO1 μHD and either tdRFP-EPS15 (1–636) or (1–614) as indicated after fixation and staining with anti-Tac and anti-Eps15 antibodies. Enlargements of boxes as in (A). Note that the anti-Eps15 is a peptide-specific antibody raised against the extreme C terminus of the protein, which is truncated in the two tagged exogenous proteins. Scale bar, 10 μm.

An ectopic yet experimentally tractable manifestation of μHD partner engagement occurs in cultured cells in the context of a customized membrane-anchored Tac-FCHO1 μHD fusion protein (Umasankar et al., 2014). Transmembrane Tac, the α chain of the interleukin-2 receptor also termed CD25, normally progresses through the biosynthetic pathway before residing at the plasma membrane (Humphrey et al., 1993). When overexpressed, a newly synthesized pool of Tac is typically seen in the Golgi en route to the surface. In Tac-μHD-, but not Tac-expressing cells, endogenous EPS15 relocates prominently to the juxtanuclear Golgi region where the cytosol-oriented μHD is highly concentrated (Figures 2A and 2B). This Tac-μHD can act as a near-quantitative sink for endogenous EPS15 in highly expressing cells, depleting EPS15 from CCSs (Figures 2A and 2C). The short DPF tract between EPS15 residues 615 and 636 promotes most effective translocation of a transfected tdRFP-full-length EPS15 onto Tac-μHD-rich membranes in the Golgi region (Figures 2C–2E). Similar ectopic concentration of the RFP-tagged full-length EPS15 does not occur upon co-transfection with Tac alone (although a pool of Tac also concentrates in the Golgi area) and is considerably reduced upon internal deletion of residues 615–636 within the tdRFP-EPS15 reporter (Figures 2D and 2E). Comparing the intracellular positioning of transfected tdRFP-EPS15 that is truncated after residue 636 with the endogenous EPS15 shows the atypical deposition of both proteins at Tac-μHD-positive organelles within transfected cells (Figure 2F). Further C-terminal truncation of tdRFP-tagged EPS15 at residue 615 produces little clustering in the juxtanuclear area, despite redistribution of endogenous EPS15 by ectopic Tac-μHD in the same cells (Figure 2G). Altogether, the results emphasize the key contribution of the mapped EPS15 DPF stretch to μHD engagement intracellularly, and suggest that these interactions play a key role in clustering these EFC-domain proteins at clathrin buds.

### Molecular Architecture of the μHD Interaction Interface

To locate the molecular surface of the μHD that physically contacts Eps15, we attempted crystallization of μHDs from several species, both alone and in complex with EPS15 peptides. When all efforts failed, the tailored chimeras (Figure 1F) were evaluated. A fusion of EPS15 (designated ^E^T615-^E^G637) to the zebrafish Fcho1 μHD (residues 867–1,152) with a GAGA spacer crystallized and diffracted to 2.4 Å. The structure was solved by single-wavelength anomalous dispersion using an Xe derivative (Table S1). In the final structure, excellent-quality electron density is visible for residues ^E^F620-^E^G637 of EPS15 (Figure S2A) and P874-L1152 of the μHD. There is no evidence of the spacer residues separating the EPS15 and μHD sections, but distance constraints make it highly likely that the interaction is intramolecular.

**Figure 3:**
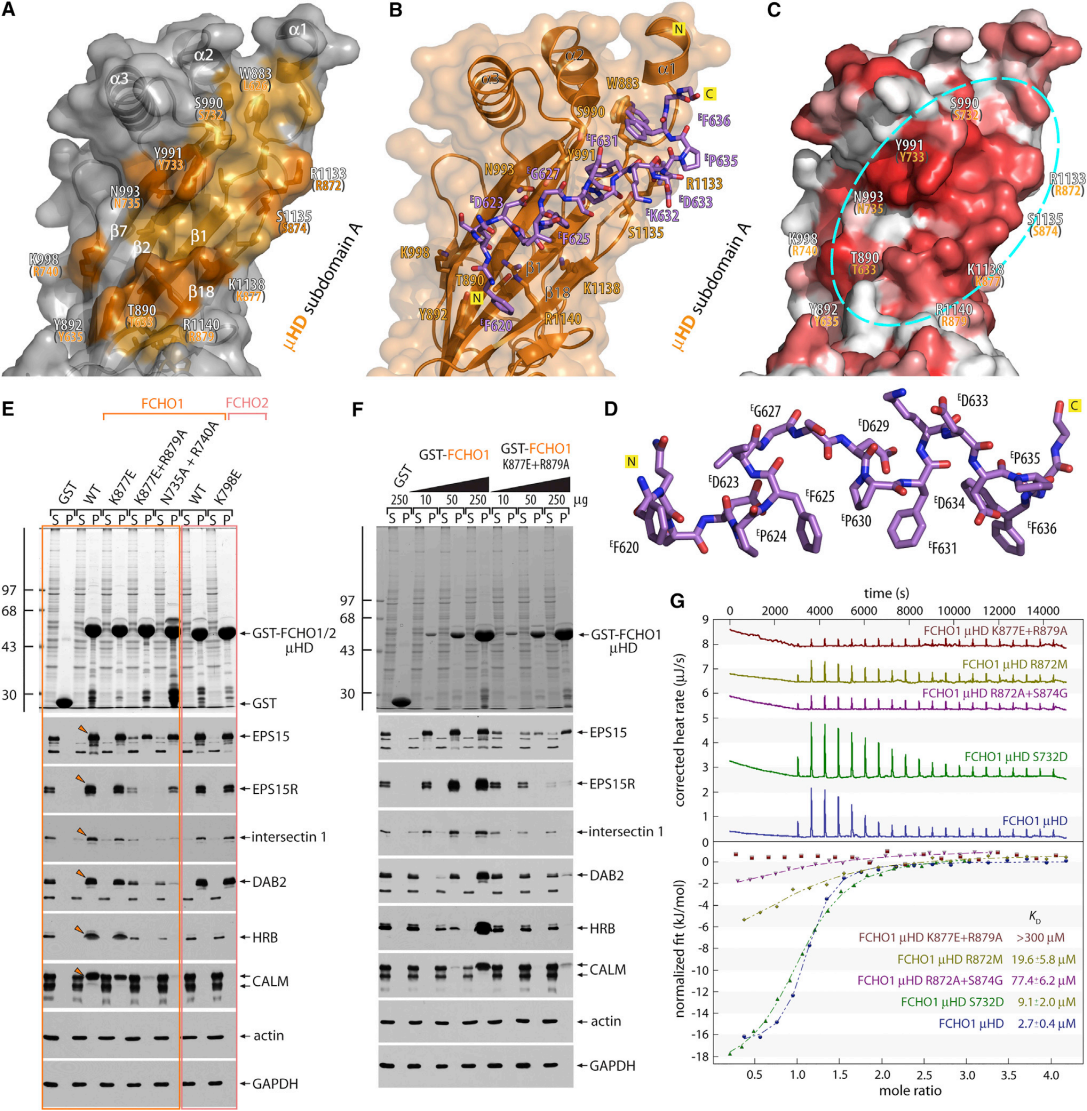
A Major Interaction Surface on Fcho1 μHD Subdomain A (A–C) Combination ribbon and molecular surface representation of the concave face of zebrafish Fcho1 μHD subdomain A showing the EPS15 binding trough. (A) The β strands involved and projecting μHD contact residues (light orange), determined by the PISA server. Selected side chains mutated are indicated (dark orange) with the corresponding residue number in the FCHO1 μHD indicated in orange type. (B) View of the tandem DFP motifs (stick representation with carbon mauve, nitrogen blue, and oxygen atoms red) bound to the μHD. ^E^F636 is shown in a dual conformation with μHD W833 also in dual conformation to permit flipping of ^E^F636. (C) ENDscript 2 (Robert and Gouet, 2014) computed phylogenetic surface conservation between muniscin μHDs using the TrEMBL (opisthokonta) database for sequence alignments. Conservation graded in shades from invariant (red) to unrelated (white) projected onto the solvent-accessible molecular surface of the Fcho1 μHD. The surface conservation reveals a patch (cyan oval) of invariant and highly conserved residues at the binding site on subdomain A, while there is a rather discombobulated patchwork pattern of conservation over the convex face. (D) The bound DPF tract showing the spatial arrangement of the aromatic Phe and Pro side chains packing into the μHD trough. (E) Pull-down assay utilizing HeLa cell lysate and 250 μg of GST, GST-FCHO1 (orange box), or GST-FCHO2 (pink box) μHD, or the indicated mutant. Stained gel and replicate blots were probed with the indicated antibodies. Position of bound partner protein (arrowheads) is indicated. (F) Pull-down assay with HeLa cell lysate and the indicated amount of GST, GST-FCHO1 μHD, or K877E + R879A mutant immobilized on glutathione-Sepharose. (G) Representative ITC experiments of wild-type FCHO1 or color-coded mutant μHDs binding to an EPS15 616–638 peptide.

Like other μ subunits, the Fcho1 μHD contains 18 β strands arranged as two intercalated β-sandwich subdomains, the fold being comparable with the orthologous *Saccharomyces cerevisiae* Syp1p μHD (Reider et al., 2009) and μ1–μ4 (Figures S2A–S2F). Yet the Fcho1 μHD also contains three additional α helices in subdomain A (Figures S2A–S2C), and the concave face of the Fcho1 μHD is considerably more curved than other μHDs. The EPS15 segment binds in a spiral trough that runs halfway around the long axis of subdomain A, with the Fcho μHD-unique α helices forming a considerable part of the trough (Figure 3A). The trough is lined with conserved largely hydrophobic residues, and significant contacts with the protein are only through the three DPF motif side chains of the sequence ^623^DPFVGSDPFKDDPF (Figures 3A–3D); the interaction buries 1,640 Å^2^ of solvent-accessible area. The electron density indicates well-ordered DPFs; the first two adopt type I tight turn conformations (Figures 3D and S2A) stabilized by intramolecular H bonding between the Asp residue side chains and the backbone amide of the +2-position Phe residues. In the last DPF, the side chain of ^E^D634 additionally forms a salt bridge with R1133. In this way, the ordered triple-DPF motif projects a stabilized contiguous hydrophobic surface into the trough with the Pro residues orienting the Phe side chains to closely match the complementary μHD interaction surface (Figure 3D). Thus the structure strongly suggests that the short tri- (VGS) or di- (KD) residue spacing between tandem DPFs is important to allow three to simultaneously dock onto a single Fcho1 subdomain A with a 1:1 stoichiometry. The weakest EPS15 peptide electron density is around G627, reflecting inherent mobility of non-chiral G residues and suggesting it may be a flexible pivot. Our interpretation is that, depending on the binding partner, the trough can be either fully or partially occupied. The site is therefore “plastic,” with partners evolving appropriate strength interactions by filling different portions of the extended trough. This contrasts with other interactions in vesicle coat formation whereby a small binding cavity on a folded protein accommodates a single small motif, and variations in affinity occur through different motif copy numbers. Below, we use the human FCHO1 μHD to confirm the binding mechanism (substantiating that the interaction surface in the structure is not an artifact of using the chimera); zebrafish and human Fcho1 μHDs are 56% identical and bind EPS15 with similar *K*_D_s (Figure 1), but FCHO1 μHD has better biochemical behavior. Residue numbers that follow are thus for the equivalent human μHD (Figure S2B).

Structure-guided mutation of FCHO1 μHD residues located near the N-terminal region of the docked EPS15 peptide verifies the role of the binding trough. A number of single substitutions in this general area each have only slightly decreased binding properties (Figures 3 and S3), indicating that multiple alterations to the extended contact interface upon the μHD are necessary to effectively disrupt binding. For compound double mutants in this part of subdomain A, the most detrimental is a K877E + R879A substitution at the outer rim of the trough. Here, association of all weak binding partners is abolished and the recovery of the strongest binding partner (native EPS15 dimer/tetramer) is reduced at least 25-fold (Figures 3E and 3F). ITC (Figure 3G) reveals binding to EPS15 peptide (616–638) is reduced to undetectable levels (*K*_D_ > 300 μM versus 2.7 μM for wild-type μHD). Simultaneous alteration of the nearby conserved residues N735A + R740A has a different and weaker inhibitory effect: durable EPS15/R binding still persists, but HRB and CALM associations are lost (Figure 3E).

Subdomain A mutations in the α-helical region located close to the second DPFX_2_DPF repeat again affect binding (Figures S3A and S3B). Besides a Y733A substitution, which is insoluble (all others were correctly folded judged by gel filtration and circular dichroism), a P627D + A629E switch is most detrimental to all cytosolic binding partners in pull-down assays. ITC using the EPS15 (616–638) peptide shows essentially no (*K*_D_ > 300 μM) binding for the A629E or P627D + A629E substitutions (Figure S3C). Other nearby substitutions, such as S732D and R872A + S874G, produce weaker inhibitory effects, most conspicuously on EPS15/R. In sum, the effects of the various structure-guided substitutions show that different partners occupy a common extended μHD surface but display different binding affinities, likely related to occupancy of different portions of the trough, and that Eps15 engagement involves higher-order decoding of adjacent DPF motifs filling the trough.

Several binding-trough amino acids differ in the Fcho2 and Sgip1 μHDs; the tripeptide ^633^TXY in strand β1 at the base of the trough is [TS]X[ST] in Fcho2 and Sgip1 orthologs (Figure S3D). Yet the noted selectivity between FCHO1 and FCHO2/Sgip1 for HRB and CALM binding (Figure S1E) cannot be solely attributed to this Y635T/S dichotomy. An FCHO1 Y635S alteration does not prevent binding of either HRB or CALM (Figure S3E). The adjacent T633Y substitution produces a modest gain-of-function effect for the weaker partners, HRB and CALM. These results illustrate that varied μHD partner motifs have evolved not an optimal but rather an acceptable fit, given the temporal and spatial requirements of these interactions in vivo.

The muniscin μHD, AP μ subunits, and δ-COP (Suckling et al., 2015) μHD all evolved from the TCUP subunit of the ancestral TSET/TPLATE sorting complex (Gadeyne et al., 2014, Hirst et al., 2014). Our work shows that in addition to conservation of the overall architectural fold, the general locale of the main peptide contact surfaces on the concave face of subdomain A is broadly similar but the molecular mechanism of peptide binding is different (Figure S2); the muniscin interaction is more expansive and is not of a β-augmentation type.

### μHD Interactions In Vivo

Unfortunately the functional role of the μHD cannot be explored simply in cells by direct deletion or mutation of full-length proteins, because forced overexpression of the Fcho1/2 EFC+ linker augments CME by promoting AP-2 opening (Hollopeter et al., 2014, Umasankar et al., 2014). The unstructured interdomain linkers of Fcho1, Fcho2, and Sgip1 all bind physically to the heterotetrameric AP-2 adaptor core, and this biochemical activity is correlated with improved AP-2-dependent cargo uptake (Hollopeter et al., 2014, Umasankar et al., 2014). When expressed ectopically in HeLa cells as a Tac-fusion protein, the FCHO1 linker alone improves transferrin internalization (Umasankar et al., 2014). Paradoxically, these results make it appear superficially as if the μHD is functionally unnecessary. However, of all the pioneers the muniscins are present as limiting components (Figure S1I); overexpression studies evade this level of regulation by copy number. As outlined below, we hypothesize that it is precisely this low abundance of FCHO1/2 that makes the concerted transactions between the μHD and the interdomain linker physiologically relevant.

Unlike the Tac-μHD (Figure S4A), a K877E + R879A mutation prohibits overexpressed Tac-μHD from abnormally clustering EPS15 in the juxtanuclear region in transfected HeLa cells; EPS15 localization is much like control Tac-transfected cells (Figures S4B and S4C). Of all the cytosolic proteins that bind to the μHD in pulldowns, only EPS15 and HRB prominently change intracellular placement in the presence of wild-type Tac-μHD, although some HRB surface puncta remain. Intersectin 1, DAB2, and CALM do not show pronounced changes in plasma membrane surface deposition compared with cells expressing Tac only (Figures S4A–S4F). This could reflect the densely redundant protein-protein and protein-lipid interaction networks these other μHD partner CLASPs display at surface CCSs (Figure S1I).

### Ectopic μHD Expression in FCHO1/2-Null Cells

**Figure 4:**
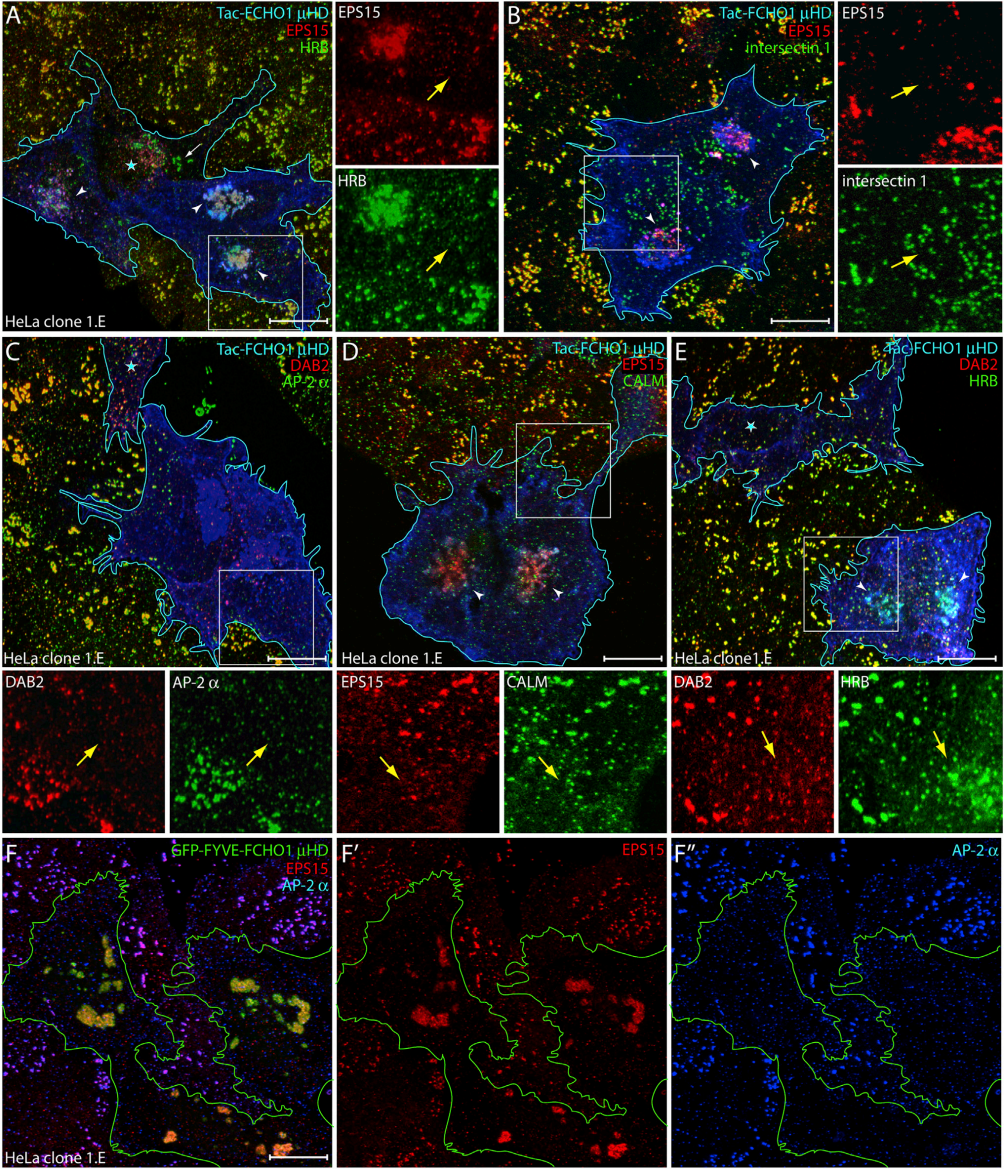
Altered CCSs in Tac-μHD-Transfected HeLa 1.E Cells (A–E) Deconvolved maximal projections of HeLa clone 1.E cells transfected with Tac-μHD and stained with the indicated antibody combinations. Transfected cells (blue outline), misrecruited juxtanuclear EPS15 and/or HRB (arrowheads), and altered pioneer/CLASP distributions (yellow arrows) are indicated. Sequestration is dose dependent; residual HRB-positive enlarged CCSs (white arrow) in a low Tac-μHD expresser (asterisk) are indicated in (A). Scale bar, 10 μm. (F–F″) Representative deconvolved maximal projection of GFP-FYVE-μHD transfected (green outline) HeLa 1.E cells stained with the indicated antibodies. Scale bar, 10 μm.

To examine this further, we overexpressed Tac-μHD in HeLa clone 1.E cells. This genome-edited clone is functionally FCHO1+2 null and displays clustered, enlarged surface CCSs at steady state (Umasankar et al., 2014). In 1.E cells, ectopic Tac-μHD again causes strong mislocalization of EPS15 and HRB (Figure 4A). Yet intersectin 1, DAB2, and CALM still do not mass prominently in the Golgi region when Tac-μHD is expressed (Figures 4B–4E). Regardless, the ectopic Tac-μHD strikingly alters endocytic CCS appearance in the muniscin-null cells; different to adjacent non-transfected 1.E cells, the Tac-μHD producers (identified by Tac staining and/or Golgi mislocalized EPS15 or HRB) exhibit small, dim, and generally dispersed AP-2-positive structures (Figure 4C). AP-2, intersectin 1, DAB2, CALM, and HRB, which co-populate most enlarged CCSs in parental 1.E cells, spatially segregate into non-overlapping spots when wild-type Tac-μHD is present (Figures 4B–4E). The scattered appearance of these coat components in the presence of membrane-anchored μHD resembles the population of smallest CCSs, increased in frequency in parental 1.E cells (Umasankar et al., 2014). Analogous alterations to AP-2-positive puncta occur when overexpressed FCHO1 μHD is instead targeted to endosomes by fusion to paired FYVE domains (Figure 4F). Again, massive intracellular EPS15 sequestration occurs and surface AP-2 puncta diminish in size and intensity. The μHD of Sgip1, which does not interact directly with either HRB or CALM (Figure S1E), also causes a reorganization of surface AP-2 when overexpressed in HeLa 1.E cells as a Tac-fusion protein (Figures S5A–S5F). Under our experimental conditions, we cannot completely exclude that the ectopic Tac-μHD we use to move EPS15 and EPS15R onto intracellular membranes does not alter subtly the precise location or dynamics of the cytosolic pools of other μHD-binding partners, including DAB2, HRB, and CALM. But even were this the case, it still attests to the fundamental importance of the μHD interaction surface we delineate here in coordinating protein-protein contacts at nascent bud sites. That EPS15 misrecruitment is not accompanied by large-scale intracellular deposition of AP-2 also reinforces that the different affinities that μHDs and AP-2 appendages display for DPFs also manifest in vivo, and makes plain the requirement for an appropriate acceptor membrane to promote AP-2 switching from the cytosolic closed state to the membrane-docked, cargo-binding-competent open state.

The phenotypic effect of combined siRNA silencing of *EPS15* and *EPS15R* transcripts in clone 1.E cells is also consistent with a direct role for EPS15/R in stabilizing nascent AP-2 assemblies at the cell surface; in cells with strongly diminished EPS15 there is a shift in the distribution of AP-2 puncta to the smallest-size populations (Figures S5G–S5L). Our results thus suggest that in the combined absence of muniscins and EPS15/R, a lack of coat consolidation/stabilization follows random clathrin-coat component encounters with the plasma membrane, accounting for the small dim puncta. Because neither intersectin, CALM, DAB2, nor AP-2 accumulates on concentrated intracellular Tac-μHD, this tactic to selectively mislocalize EPS15 (and HRB) in muniscin-null cells allows further functional dissection of the EPS15/R⋅μHD interaction in CME using transferrin uptake assays and total internal reflection fluorescence microscopy (TIRFM).

### Impact of μHD Interactions on CME

First, we assessed ligand uptake. On a 2-min pulse, fluorescently labeled transferrin clusters at, and is internalized from, abnormally enlarged CCSs in parental 1.E cells. Obviously different from this surrounding untransfected 1.E cell population, the Tac-μHD transfected 1.E cells, which display bright intracellular EPS15 staining and dispersed small faint plasma membrane dots of AP-2, accumulate transferrin diffusely over the plasma membrane, and uptake into endosomes is strongly slowed (Figures S6A–S6A″′), indicating defective CME. Even after 10 min of incubation with transferrin, Tac-μHD-producing cells with dim AP-2 spots internalize very little cargo relative to neighboring untransfected 1.E cells with typically large CCSs (Figures S6B–S6B″′). Thus the fine AP-2 puncta at the surface do not efficiently package the prototypical YXXØ-type signal cargo protein, the transferrin receptor when FCHO1/2 and EPS15 are limiting (i.e., depleted from the vicinity of the plasma membrane). These results are in stark contrast to our previous findings regarding the effect of transfecting the same FCHO1/2-null 1.E HeLa cells with Tac fused instead to the preceding FCHO1 interdomain linker (residues 265–609) (Umasankar et al., 2014). This disordered segment of FCHO1 between the folded EFC domain and μHD also alters the steady-state morphology of CCSs to small regular puncta, but in this case they are brighter (contain more AP-2) and transferrin uptake is actually stimulated (Figures S6C–S6C″′) as the linker on the plasma membrane can trigger AP-2 to adopt an open, cargo-binding conformation (Hollopeter et al., 2014, Umasankar et al., 2014). In fact, in *Caenorhabditis elegans*, numerous *fcho-1*-null bypass suppressor mutations in AP-2 subunits, which shift the equilibrium of AP-2 to the open state, completely negate any requirement for FCHO-1 in vivo (Hollopeter et al., 2014).

**Figure 5:**
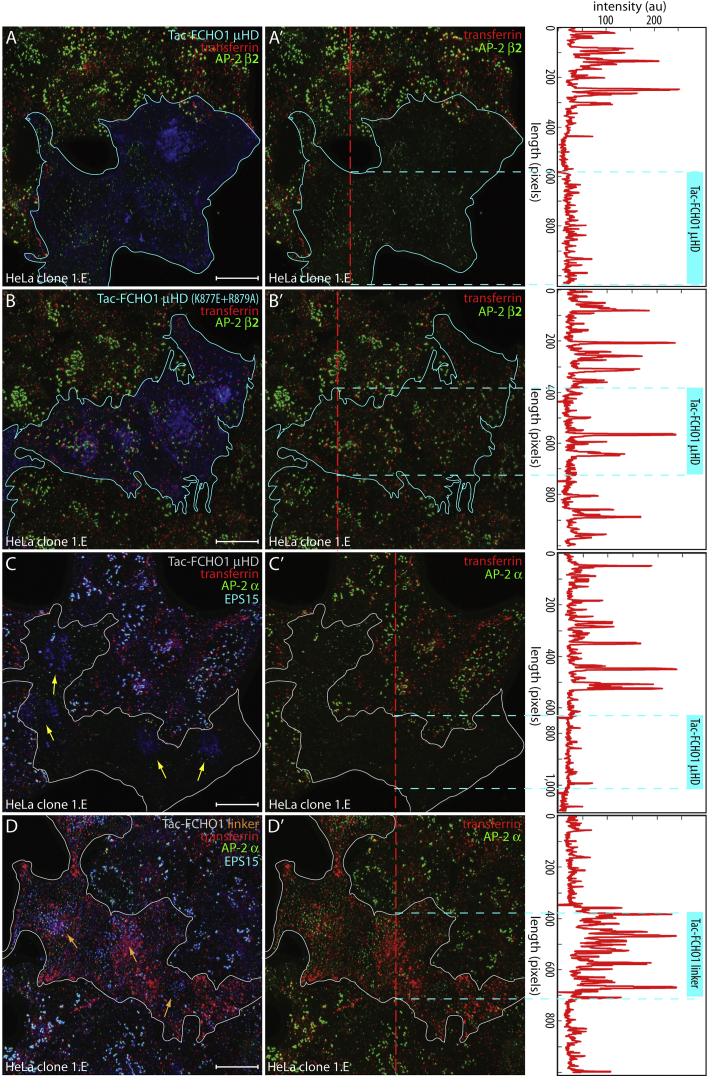
Defective CME in Tac-μHD-Expressing 1.E Cells (A–B′) HeLa clone 1.E cells transfected with either Tac-μHD (A) or the Tac-μHD (K877E + R879A) mutant (B) and pulsed with transferrin (red) for 2 min at 37°C, surface stripped on ice, and rewarmed to 37°C for another 2 min before fixation. Representative deconvolved maximal intensity z projection of permeabilized cells stained with antibodies against Tac and the AP-2 β2 subunit is indicated. Transfected cell groups are outlined, and superimposed transferrin and AP-2 shown in (A′) and (B′). Scale bar, 10 μm. Correlative linescan analysis at indicated locations in A′ and B′ (dashed vertical line) is shown on the right, with relative position of transfected cells indicated with a vertical cyan bar and horizontal broken cyan lines. (C–D′) Deconvolved z stack projections of 1.E cells transfected with either Tac-μHD (C) or Tac-linker (D) and similarly pulsed with transferrin as in (A) and (B). Fixed and permeabilized cells were stained with antibodies against the AP-2 α subunit and EPS15 as indicated. Tac-μHD-dependent intracellular clustering of EPS15 (yellow arrows) and diminished transferrin internalization contrasts the retention of EPS15 in regular AP-2-positive surface puncta (orange arrows) and enhanced endocytosis in presence of the Tac-linker. Scale bar, 10 μm. Correlative linescan analysis at indicated locations in (C′) and (D′) (red dashed line) is shown on the right, with relative position of transfected cells indicated as in (A) and (B).

Definitive evidence for defective CME comes from cells pulsed with transferrin at 37°C for 2 min, surface stripped on ice, and reheated to 37°C for an additional 2 min. The peripheral transferrin-positive early endosomes abundant in surrounding untransfected 1.E cells are diminished in Tac-μHD-producing 1.E cells that also exhibit altered AP-2 spots (Figures 5A and S6D). By comparison, 1.E cells expressing Tac-μHD (K877E + R879A) have transferrin and AP-2 staining roughly comparable with adjacent untransfected cells (Figure 5B). In transferrin-pulsed 1.E cells labeled for AP-2 and also for EPS15 to observe internal sequestration of this pioneer by Tac-μHD, transferrin uptake is sharply reduced (Figure 5C). By contrast, Tac-linker-positive cells have a characteristic changed AP-2 arrangement, and more numerous and medial transferrin-positive endosomes (Figures 5D and S6D). The observed AP-2 puncta in either Tac-μHD- or Tac-linker-producing 1.E cells are therefore functionally distinct, with the explanation for the opposite outcomes on transferrin internalization being that the Tac-μHD exerts a dominant sequestration effect on EPS15 at intracellular membranes while the Tac-linker works by gain of function on AP-2 at the plasma membrane (Umasankar et al., 2014).

**Figure 6:**
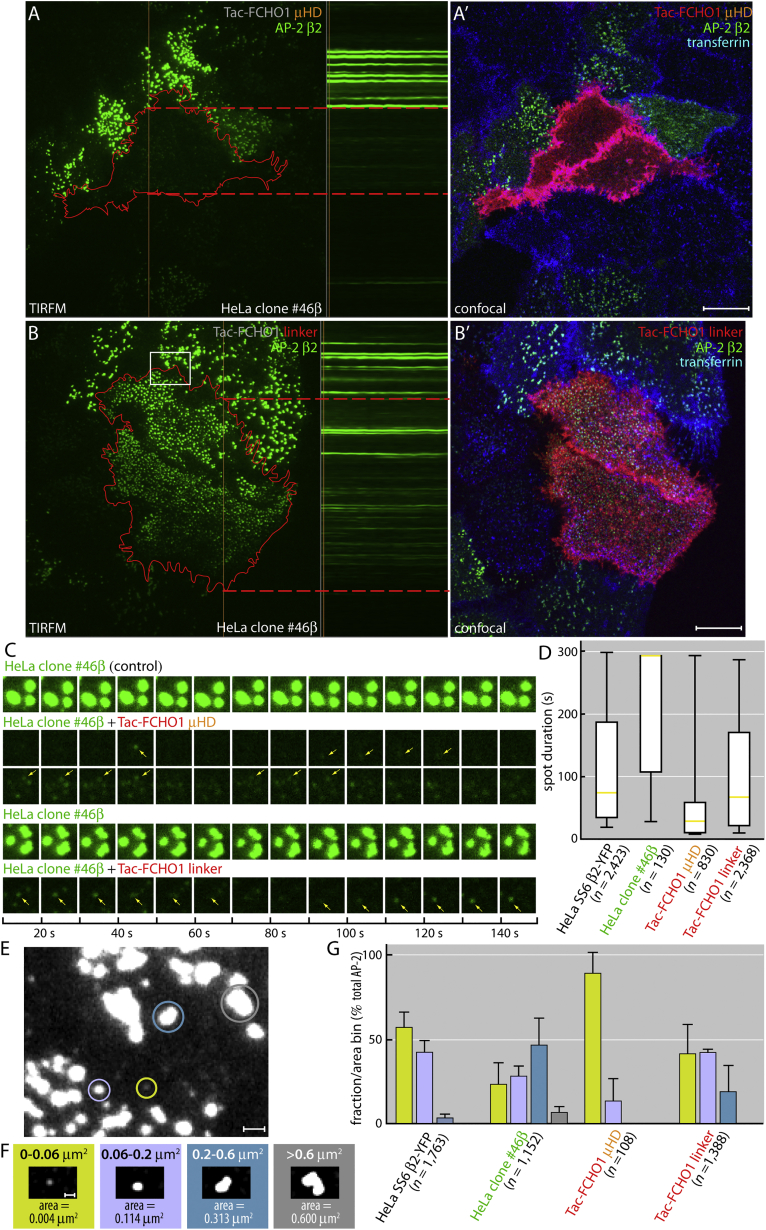
Uptake Defects in FCHO2 and EPS15 Compromised Cells (A–B′) TIRFM analysis of HeLa clone #46β cells transfected with either Tac-μHD (A) or Tac-linker (B). The initial frame of the kymograph series (left), the kymograph (center), and a final confocal image (A′, B′) of the same area after labeling with anti-Tac and fluorescent transferrin (right) are shown. A single pixel-width vertical line (orange) on the first TIRFM fame (left) indicates the coordinates for the kymograph (center panel), and the relative locations of the transfected cells in the kymographs are marked (dashed horizontal red lines). Scale bar, 10 μm. (C) Time-lapse examples of local β2-YFP puncta selected from the image series in (A) and (B) to detail the major differences between the long-lived large structures in the control clone 1.E cells compared with the transfected cells. Representative individual spot life times (arrows) are indicated. (D) Box-and-whisker (minimum-maximum) plot quantitation of computed spot durations from the kymographs in (A) and (B), and also compared with the parental HeLa SS6 β2-YFP cells. Median is indicated in yellow and the number of fluorescent puncta analyzed/condition is indicated. (E–G) Enlarged gray-scale view (E) of region boxed in (B) illustrating selected examples of the color-coded spot area bins (F) used in quantitation (G). The SD and number of spots analyzed for each condition are indicated. Scale bars, 1 μm (E) and 0.5 μm (F).

The AP-2 puncta in Tac-μHD-expressing 1.E cells appear similar to the small abortive CCSs that arise in cells expressing AP-2 lacking the α-subunit appendage (Aguet et al., 2013), which binds directly to single EPS15 DPFs among other ligands (Brett et al., 2002, Praefcke et al., 2004). However, marked surface transferrin accumulation shows that Tac-μHD-transfected HeLa 1.E cells do not display compensatory CME, like cells with endogenous Eps15 and Fcho1/2 (Aguet et al., 2013, Motley et al., 2006). Indeed, time-resolved imaging of β2-YFP-marked AP-2 puncta on the bottom of FCHO2-null HeLa cells (clone #46β, Figures S7A–S7E) transfected with Tac-μHD reveals highly dynamic, dim, short-lived (presumably unproductive or abortive) spots (Figures 6A and 6C), explaining the accumulating transferrin at the plasma membrane. For these live-cell studies we used the FCHO2 gene-disrupted HeLa clone #46β cells because FCHO1 protein levels are extremely low in HeLa cells (Hein et al., 2015, Umasankar et al., 2012) and the endocytic phenotype and behavior of FCHO2-null and FCHO1/2-null HeLa cells are indistinguishable (Umasankar et al., 2014). Similarly to HeLa and clone 1.E cells, ectopic Tac-μHD redirects endogenous EPS15 to a perinuclear location in the clone #46β cells (Figures S7F and S7G). The time-resolved imaging experiments show importantly that defective diminutive AP-2 puncta occur in living Tac-μHD-producing cells and that they display abnormal kinetic behavior (Figures 6C and 6D). In parental #46β cells, 75% of patch lifetimes are >108 s (median spot duration ∼294 s). When expressing the Tac-μHD, the median spot duration in these cells becomes ∼30 s compared with ∼74 s for cells expressing the Tac-linker (Figure 6D). The kinetic behavior (median duration ∼75 s) and area distributions of AP-2 puncta in Tac-linker expressing clone #46β cells closely resemble HeLa SS6 β2-YFP cells, from which the #46β cells were derived (Figures 6D–6G). Collectively these experiments show that, alone, the Fcho μHD or Fcho linker can change the morphology, kinetic signature, and cargo-sorting capacity of surface CCSs in muniscin-null cells. Since the interdomain linkers of Fcho2 and Sgip1 also affect AP-2 complex conformation (Hollopeter et al., 2014, Umasankar et al., 2014), our findings underscore the functional adaptation and importance of sequentially arranged regions of the low-abundance muniscins (Figure S1I). In cells, we propose that the linked muniscin domains act on AP-2 in concert by virtue of the protein-binding properties of the μHD; they bring together the necessary protein machinery to switch AP-2 from the basal closed conformational state to the open active form.

## Discussion

**Figure 7:**
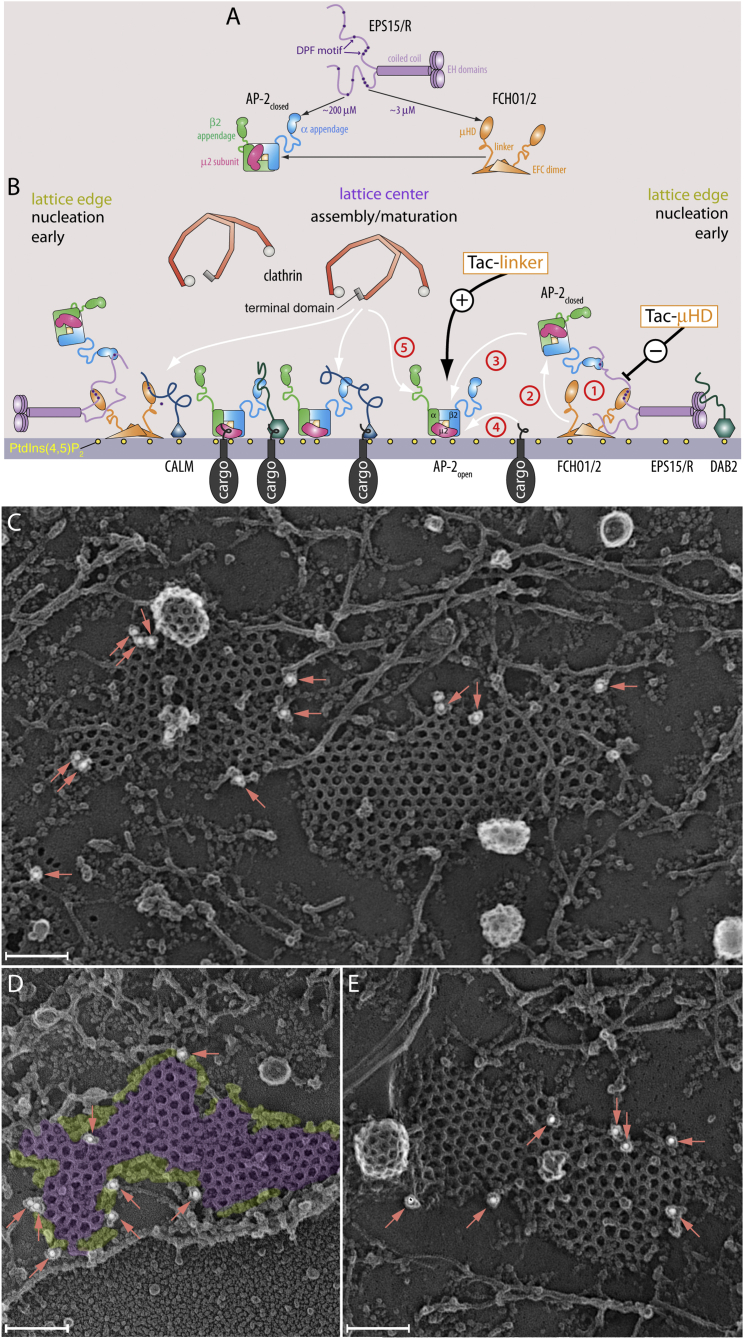
Transient Higher-Order Complexes to Load Active AP-2 at Growing Assembly Zones (A and B) Schematic models for edge effects on vectorial CCS initiation. The DPF code (A) will have consequences for the formation and stability of Eps15⋅Fcho1/2, Eps15⋅AP-2, and Eps15⋅Fcho1/2⋅AP-2 nanoclusters. Because of the higher apparent affinity of the Fcho μHD for the DPFs in Eps15/R, formation of Eps15⋅Fcho1/2 will be favored at inchoate sites of CCS assembly. (C–E) Representative deep-etch electron microscopic image views of adherent ventral cell membranes of HeLa SS6 cells immunogold labeled for FCHO2 with 18 nm colloidal gold (arrows). Proteins assembled around the perimeter (chartreuse) of characteristic polyhedrally assembled clathrin lattice (purple) are pseudo-colored (C). Scale bar, 100 nm.

This work explores how interactions between Fcho1/2, Eps15/R, and AP-2 contribute to CCS nucleation. While Eps15 engages Fcho1/2 and AP-2 in fundamentally different and noncompeting ways, both intriguingly depend on DPF triplet-based recognition (Brett et al., 2002, Praefcke et al., 2004). Within the Eps15 unstructured region, residues 600–650 contain seven DPF repeats that, with limiting spacing requirements, bind Fcho1/2 as a higher-level triple-DPF motif. AP-2 binding to distal Eps15 residues 650–740 (Benmerah et al., 1996, Iannolo et al., 1997) depends on only single DPF motifs (Brett et al., 2002, Praefcke et al., 2004). Relatively tight apparent binding is due to avidity phenomena: multiple α appendages from independent AP-2 heterotetramers bind to the array of single DPFs (Praefcke et al., 2004) on the Eps15 dimer/tetramer (Cupers et al., 1997), and is further enhanced by the AP-2 β2 appendage also binding Eps15, albeit through a different Phe-rich motif (Edeling et al., 2006, Schmid et al., 2006). While this synchronous binding of separate DPFs to individual AP-2 appendages is facilitated by unstructured intervening peptide stretches between DPFs, strict inter-motif intervals are unimportant. Consequently, spacing constraints have allowed Eps15/R to separately tune DPF-binding selectivities for μHDs and AP-2 appendages (Figure 7A). The two proteins bind independently of one another; docking of a triple-DPF motif onto a μHD does not affect the remaining unstructured Eps15 C terminus, leaving it free to engage one or more AP-2 adaptors. The tighter binding and slower off-rate of a triple-DPF⋅μHD interaction (*K*_D_ ≅ 3 μM) compared with a single DPF⋅α-appendage interaction (*K*_D_ ∼ 200 μM) (Olesen et al., 2008) favors initial Fcho1/2⋅Eps15/R heterodimer formation (Figure 7A). On the plasma membrane, at a forming CCS, where clustered AP-2 is bound with low micromolar affinity and oriented by PtdIns(4,5)P_2_, the high off-rate of a lone DPF from an appendage is less critical; fast rebinding would occur, and since multiple appendages can simultaneously be engaged by the distal Eps15/R C termini of Eps15 oligomers, Fcho1/2⋅Eps15/R⋅AP2 nanocluster formation is favored.

### An Endocytic CCS Commencement Model

In HeLa clone 1.E cells lacking muniscins, EPS15/R can direct the assembly of stable, enlarged, long-lived CCSs with delayed CME (Umasankar et al., 2014). Silencing of EPS15/R (Teckchandani et al., 2012) or reconstitution of knocked-down AP-2 cells with an α-appendageless AP-2 heterotetramer (Aguet et al., 2013) similarly causes enlarged, clustered, and functionally abnormal CCSs. We now show that without both FCHO1/2 and EPS15/R, CCSs are short-lived and CME is further disrupted. This information points to an interdependent three-way relationship between muniscins, Eps15/R, and AP-2. So how does the deciphered DPF code support productive CCS formation? Extinguishing clathrin expression in HeLa cells by RNAi blocks CME, but has little impact on deposition of a planar AP-2, EPS15, and CALM matrix on the plasma membrane (Hinrichsen et al., 2003, Meyerholz et al., 2005, Motley et al., 2003). This indicates that adaptors/CLASPs require clathrin, in all probability by clustering CLASPs to high local density, for proper membrane deformation, but that initial congregation of pioneers at emerging buds is largely clathrin independent (Hinrichsen et al., 2006). By contrast, AP-2 transcript silencing leads to diminutive CCSs (Miller et al., 2015, Motley et al., 2003). One interpretation of this is that AP-2 deposition at nascent assembly zones expands the developing coat by loading the interior of a pioneer-rich patch, with open AP-2 able to engage cargo (Figure 7B). How would this happen? AP-2, Eps15/R, and Fcho/2 differ considerably in projected copy number/cell (Hein et al., 2015) and, hence, attainable stoichiometry at individual assembly zones (Borner et al., 2012). When an EFC domain docks on the negatively charged plasma membrane (Umasankar et al., 2012), Fcho1/2⋅Eps15/R complexes (Figure 7B, step 1) could sequentially bind to (step 2) and then deposit AP-2 adaptors in the immediate vicinity, aided by interaction of the AP-2 α/β2-subunit trunks with plasma membrane PtdIns(4,5)P_2_. A local but temporary DPF-mediated three-component nanocluster would allow AP-2 to linger in the vicinity of Fcho1/2 to advance CCS assembly by encounters with the muniscin effector linker (step 3). This interaction leads to conformational rearrangement of AP-2 to an “open” or active form (Hollopeter et al., 2014, Umasankar et al., 2014) that facilitates cargo engagement (step 4) and release of the AP-2 β2-subunit hinge to promote clathrin polymerization (step 5). In this way, a large increase in AP-2 dwell time at the forming CCS results.

This scenario is grounded on the observations that despite retaining PtdIns(4,5)P_2_ binding, AP-2 without an α appendage is only weakly plasma membrane associated (Robinson, 1993); that overexpression of an N-terminally-deleted Eps15 mutant lacking EH domains 2 and 3 mislocalizes AP-2 and inhibits CME (Benmerah et al., 1999, Carbone et al., 1997); and that microinjected α appendage inhibits AP-2 deposition at CCSs (Hinrichsen et al., 2006). As the local concentration of AP-2 increases at the assembly zone, other incoming adaptors/CLASPs such as Dab2, ARH, epsin, and CALM, because they are more abundant (Figure S1I) (Hein et al., 2015), can outcompete Eps15/R for appendage binding since they all also bind PtdIns(4,5)P_2_, clathrin and cargo in addition to engaging AP-2 (Figure 7B). In this way, Eps15 and associated muniscins will be displaced from the center of the nascent CCS. Eps15/R and associated muniscins become positioned around the growing lattice edge while bulk AP-2 concentrates in the CCS center. Our experimental data support this hypothesis in that, for CCSs of at least moderate size in HeLa cells, FCHO2 is restricted to a band of globular proteinaceous material that we visualize surrounding assembled clathrin (Figures 7C–7E), similarly to Eps15 (Edeling et al., 2006, Henne et al., 2010, Tebar et al., 1996). Proteomic analysis also supports the model, showing that relatively little FCHO2 and EPS15/R is packaged into budded coated vesicles (Borner et al., 2012) despite their early appearance at beginning CCSs (Taylor et al., 2011). The outer rim of the forming CCS will thus delimit the perimeter and constrain productive coat polymerization to within the assembly zone. The edge effectively also conveys assembly information because remodeling of the clathrin patch, assembly of which is triggered by activation of AP-2, from an initial flat to hemispherical and then deeply invaginated profile at the center, requires polymeric clathrin (Avinoam et al., 2015, Hinrichsen et al., 2006, Miller et al., 2015, Wu et al., 2003). Finally, the proposed working model provides an explanation for the open question of how AP-2 can be massed at CCSs stoichiometrically exceeding other pioneer factors, yet depends on them in part for recruitment.

A remarkable capability of surface CCSs is to quickly produce cargo-packed transport vesicles, often in single turnover events, at variable locations on the plasma membrane. Our results and others (Mulkearns and Cooper, 2012, Teckchandani et al., 2012) reveal that although alterations in FCHO1/2:EPS15/R ratios change CCS morphology and distribution, CME does not halt under these conditions. AP-2, CALM, DAB2, and other appendage-binding CLASPs co-cluster at enlarged CCSs in FCHO1/2-null cells (Figure 4). Internalization is slowed but, given time, transmembrane cargo internalizes, substantiating that Fchos guide efficient cargo-laden vesicle production. The model we propose also provides a mechanistic rationale for the previously unexplained demonstration of defective AP-2-dependent cargo uptake in *fcho-1* mutant *C. elegans* complemented with a single-copy FCHO-1 lacking the μHD (Hollopeter et al., 2014).

In sum, it is now evident that the role of the multistep (Aguet et al., 2013) initial DPF-code-mediated Fcho1/2-Eps15-AP-2-Dab2-Hrb-CALM interactome, the details of which we illuminate here, is to first prime the endocytic process and then optimize cargo packaging. As this is the principal biological imperative for clathrin-coated vesicle production, the assembly of Fcho⋅Eps15⋅AP2 nanoclusters is at the core of efficient CME.

## Experimental Procedures

### Materials, Plasmids, Protein Expression, and Cell Culture

Standard techniques were used throughout. Detailed information about approaches and description of methods are presented in Supplemental Experimental Procedures. All experiments reported were repeated at least three times with similar results.

### Crystallization and Structure Determination

Crystals of the EPS15-Fcho1 μHD chimera grew in hanging drops against a reservoir of 100 mM Bis-Tris propane (pH 6.0), 200 mM sodium citrate, 22% polyethylene glycol 3350, and 10 mM DTT and were cryoprotected in 22% glycerol before freezing. A Xe derivative was used for phasing (Table S1).

### Biochemical Assays

Pull-down assays entail mixing glutathione-Sepharose-immobilized GST-fusion proteins with a cell extract or purified proteins, washing, and analysis of resulting supernatant and pellet fractions. For SDS-PAGE and blots, five times more of each pellet versus supernatant fraction was loaded. ITC involves repeated injection of EPS15 peptides into a temperature-controlled solution of μHD utilizing a Nano ITC instrument (TA Instruments).

### Endocytosis Assays

Prior to transferrin addition, HeLa cells were incubated for 60 min at 37°C in a starvation medium of DMEM supplemented with 25 mM HEPES and 0.5% BSA of to clear receptors of apotransferrin. Fluorescently labeled Alexa Fluor transferrin (25 μg/ml) was added in prewarmed (37°C) medium and internalization was for either 2 or 10 min at 37°C. For the transferrin pulse-chase uptake assays, starved cells were incubated with fluorescent transferrin at 37°C for 2 min and then immediately chilled on ice and washed with PBS as described by Reis et al. (2015). Surface-bound transferrin was then stripped by washing the adherent cells three times with cold 0.2 M acetic acid and 0.2 M NaCl (pH 2.5) on ice. Prewarmed DMEM containing 25 mM HEPES and 0.5% BSA was added to the stripped cells, which were then returned to a 37°C water bath for an additional 2 min before fixation.

### Microscopy

Confocal imaging was on an inverted Olympus FV1000 microscope equipped with a PlanApo N (60 ×/1.42 NA) oil objective. Excitation and emission wavelengths were preprogrammed by the instrument running the FV10-ASW software, and emission signals in the different channels were collected in the sequential scan mode. Exported TIFF file images were imported into Adobe Photoshop CS4 for minor adjustments to brightness and/or contrast. Quantitation of transferrin uptake in confocal image files was performed using the linescan application within MetaMorph (Molecular Devices). The extent of transferrin internalization in confocal image stacks that were collected following the pulse-chase format was analyzed using Fiji software (Schindelin et al., 2012). Images were corrected for the background. A 10.09 × 10.09-μm area was selected as a region of interest (ROI) in basal, middle, and upper sections of each cell in the z stack. Each optical section image was saved and processed separately. A mask for each image was created by applying a manual threshold. The mask was then used on the corresponding ROI image to measure the number and intensity of each spot in different cells. For cells in the different populations (control untransfected, Tac-μHD, or Tac-linker expressing), the intensity values of spots in each population set were pooled together and plotted in the histogram with the same total area (2,443.4 μm^2^) for comparison.

### Live-Cell Imaging

Starved cells in glass-bottomed MatTek dishes were imaged by TIRFM. Four separate positions were selected for each experiment and images were collected every 5 s for 5 min. The surface residence times of positively labeled structures were calculated using Imaris (Bitplane). The “spots” function was used to delineate AP-2 (β2-YFP)-positive structures in selected ROIs using local contrast thresholding of the TIRFM datasets. Spots were uniformly sized at slightly greater than diffraction limit (267 nm) for the 100 × 1.49 NA TIRFM objective and the wavelength of light collected. Spot persistence was tracked over time (5 s image separation) to determine duration of residency of individual structures at the cell surface. In addition, live-cell TIRFM images and immunofluorescence confocal images from fixed cells were analyzed by using Fiji and MetaMorph. Raw AP-2 channel images were background corrected. For AP-2 TIRFM datasets, 2D deconvolution was then applied to these images in MetaMorph to increase the signal-to-noise ratio. Further analysis was carried out using Fiji; individual cells were marked and saved separately. Deconvoluted images were smoothed and thresholded manually to create a mask, and masks used on the corresponding background-corrected images to calculate values for the size/area of the AP-2 puncta.

## Author Contributions

Conceptualization, L.M.T; Methodology, D.J.O. and L.M.T; Software, A.J.M; Validation, L.M., P.K.U., A.G.W., A.L., A.J., D.J.O., and L.M.T.; Formal Analysis, A.J.M., A.G.W., S.S.H., and S.C.W.; Investigation, L.M., P.K.U., A.G.W., A.L., A.J., A.J.M., D.J.O., S.S.H., T.P.-S., L.M.T., and S.C.W.; Resources, S.S.H., A.J., A.L., L.M., A.J.M., D.J.O., T.P.-S., L.M.T., P.K.U., S.C.W., and A.G.W.; Writing – Original Draft, D.J.O. and L.M.T.; Writing – Review & Editing, D.J.O., L.M.T., and P.K.U.; Visualization, D.J.O., L.M.T., P.K.U., and A.G.W.; Supervision, D.J.O. and L.M.T; Funding Acquisition, D.J.O. and L.M.T.

## Accession Numbers

The final coordinates and structure factors have been deposited in the PDB with accession code PDB: 5JP2.

## Supplemental Information

Document S1. Supplemental Experimental Procedures, Figures S1–S7, and Table S1

Document S2. Article plus Supplemental Information
