# Supplementary material for: Transient Fcho1/2⋅Eps15/R⋅AP-2 Nanoclusters Prime the AP-2 Clathrin Adaptor for Cargo Binding
Source: Dev Cell. 2016 Jun 6;37(5):428–43. doi: 10.1016/j.devcel.2016.05.003 (PMC4921775; doi:10.1016/j.devcel.2016.05.003)
Supplement: Document S1. Supplemental Experimental Procedures, Figures S1–S7, and Table S1 [file mmc1.pdf]

Developmental Cell, Volume *37*

Supplemental Information

Transient Fcho1/2·Eps15/R·AP-2 Nanoclusters

Prime the AP-2 Clathrin Adaptor for Cargo Binding

Li Ma, Perunthottathu K. Umasankar, Antoni G. Wrobel, Anastasia Lymar, Airlie J. McCoy, Sachin S. Holkar, Anupma Jha, Tirthadipa Pradhan-Sundd, Simon C. Watkins, David J. Owen, and Linton M. Traub

Inventory of Supplemental Materials:

Figure S1, related to Figure 1. The μHD protein–protein interaction web.

Figure S2, related to Figure 3. Fcho1 μHD co-crystal and evolutionary conservation of the concave μHD interaction surface upon subdomain A.

Figure S3, related to Figure 3. Additional characterization of structure-guided mutations in FCHO1 μHD subdomain A.

Figure S4, related to Figure 4. Tac-μHD lures only select endocytic pioneers/CLASPs onto intracellular compartments.

Figure S5, related to Figure 4 and 5. Remodeling of cell-surface AP-2 puncta in presence of ectopic Tac-Sgip1 μHD expression or after extinguishing EPS15 and EPS15R with transfected siRNA SMARTpools.

Figure S6, related to Figure 5. Defective CME in FCHO1/2-null HeLa 1.E cells expressing Tac-μHD.

Figure S7, related to Figure 6. Characterization of the HeLa SS6 β2-YFP clone #46β FCHO2-null line

Table S1, related to Figure 3. Data collection and refinement statistics.

Supplemental Experimental Procedures

Supplemental References

Figure S1

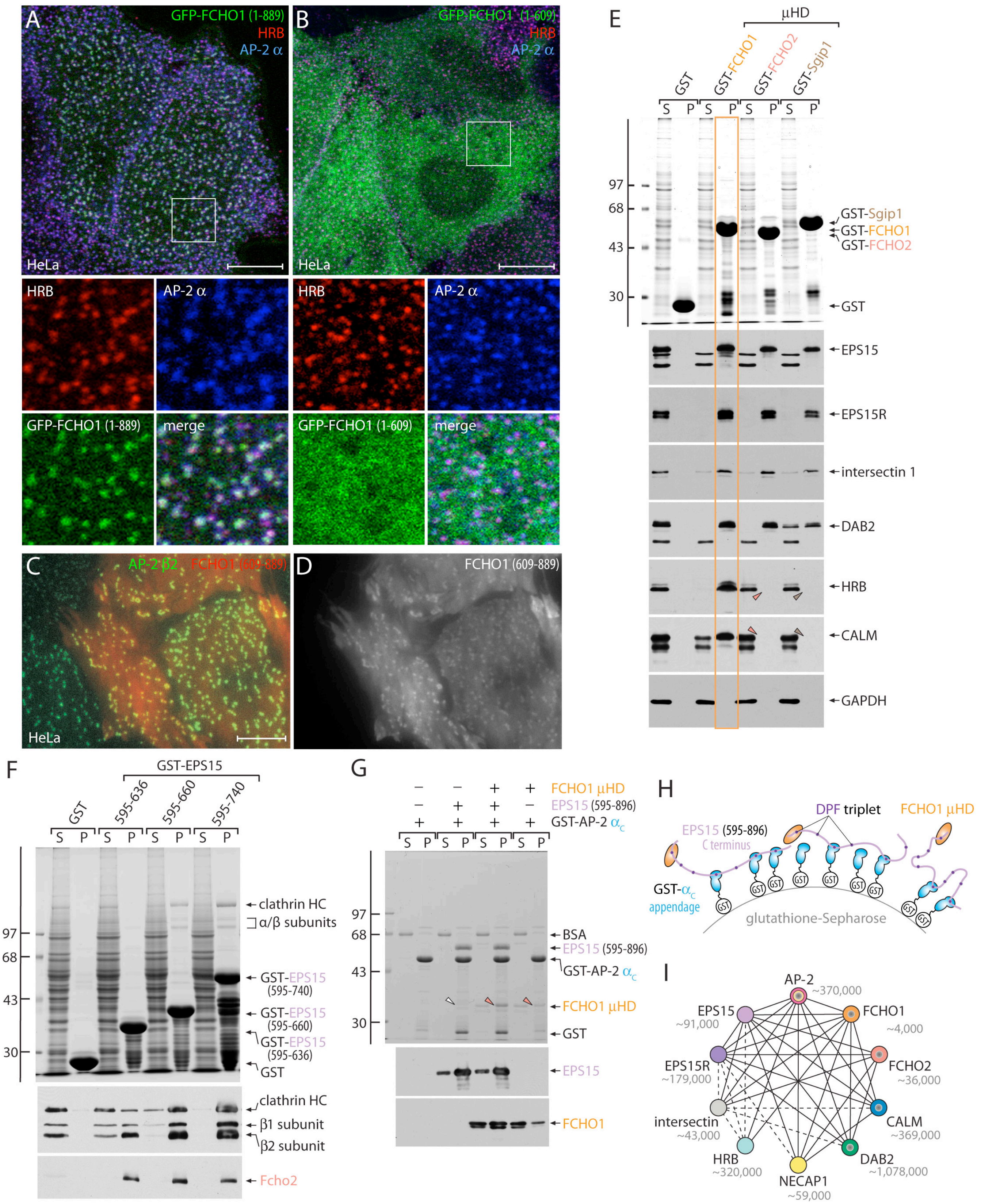

A
GFP-FCHO1 (1-889)
HRB
AP-2 α
HeLa
HRB
AP-2 α
GFP-FCHO1 (1-889)
merge
B
GFP-FCHO1 (1-609)
HRB
AP-2 α
HeLa
HRB
AP-2 α
GFP-FCHO1 (1-609)
merge
C
AP-2 β2
FCHO1 (609-889)
HeLa
D
FCHO1 (609-889)
E
µHD
GST
GST-FCHO1
GST-FCHO2
GST-Sgip1
S P S P S P S P
97
68
43
30
GST-Sgip1
GST-FCHO1
GST-FCHO2
GST
EPS15
EPS15R
intersectin 1
DAB2
HRB
CALM
GAPDH
F
GST-EPS15
GST
595-636
595-660
595-740
S P S P S P S P
97
68
43
30
clathrin HC
α/β subunits
GST-EPS15 (595-740)
GST-EPS15 (595-660)
GST-EPS15 (595-636)
GST
clathrin HC
β1 subunit
β2 subunit
Fcho2
G
FCHO1 µHD
EPS15 (595-896)
GST-AP-2 α_c
S P S P S P S P
97
68
43
30
BSA
EPS15 (595-896)
GST-AP-2 α_c
FCHO1 µHD
GST
EPS15
FCHO1
H
DPF triplet
FCHO1 µHD
EPS15 (595-896)
C terminus
GST-α_c
appendage
glutathione-Sepharose
I
AP-2
~370,000
EPS15
~91,000
FCHO1
~4,000
EPS15R
~179,000
FCHO2
~36,000
intersectin
~43,000
CALM
~369,000
HRB
~320,000
DAB2
~1,078,000
NECAP1
~59,000

Figure S1, linked to Figure 1. The μHD protein–protein interaction web

A-B. Confocal analysis of HeLa SS6 cells transfected with GFP-FCHO1 (1-889) (A) or GFP-FCHO1 (1-609) lacking the μHD (B). Fixed cells were probed with indicated antibodies against the AP-2 α subunit and HRB. Representative merged single confocal sections and enlarged color-separated regions corresponding to the boxed areas are shown. Scale bar = 10 μm.

C-D. Two-color total internal reflection fluorescence microscopy (TIRFM) of stable AP-2 β2-YFP expressing HeLa SS6 cells transfected with tdRFP-FCHO1 (609-889; μHD). A representative merged (C) and a μHD (red channel) image (D) is shown. Scale bar = 10 μm.

E. GST pull-down assays with HeLa cell lysate and 250 μg of either GST or the indicated GST-μHD fusion proteins. Aliquots of 2% of each supernantant (S) and 10% of each pellet (P) fraction were resolved by SDS-PAGE and either stained with Coomassie blue or transferred to nitrocellulose. Replicate blots were probed with the indicated antibodies. Pellet fraction showing solid phase interactions with the FCHO1 μHD is boxed (orange), while arrowheads indicate the lack of a similar interaction with the FCHO2 or Sgip1 μHD. Note that the large splice isoform of CALM binds preferentially to the FCHO1 μHD; this protein differs from the smaller isoform(s) by alternative splicing of a 50 amino-acid residue-encoding exon that contains a DPF tripeptide, supporting the direct role of DPF recognition in μHD associations.

F.GST pull-down assay with rat brain cytosol and ~250 μg of either GST or GST-EPS15 (595-636), (595-660) or (595-740) proteins immobilized on glutathione-Sepharose. Aliquots of each supernatant (2%) and pellet (10%) fraction were resolved by SDS-PAGE and either stained with Coomassie blue or transferred to nitrocellulose. Replicate blots were probed with a mixture of antibodies directed against the clathrin heavy chain (HC) and the β1/β2 subunit of AP-1/2 or an antibody against Fcho2 as indicated.

G. Two-stage pull-down assay with immobilized GST-α_C appendage incubated first with EPS15 (595-896) followed by addition of the FCHO1 μHD in presence of carrier BSA. A stained gel and corresponding blots probed with the indicated antibodies against the EPS15 C terminus and the FCHO1 μHD are shown. The migratory position of the monomeric μHD is indicated (arrowheads).

H. Schematic representation of the conceptual interpretation of the results in G.

I. Wiring diagram of the clathrin pioneer protein biophysical network. Edges show known direct interactions, broken lines denote probable direct physical interactions, and gray centers connote direct PtdIns(4,5)P_2 binding. Approximate copy number estimates in HeLa cells (Hein et al., 2015) are shown.

Figure S2

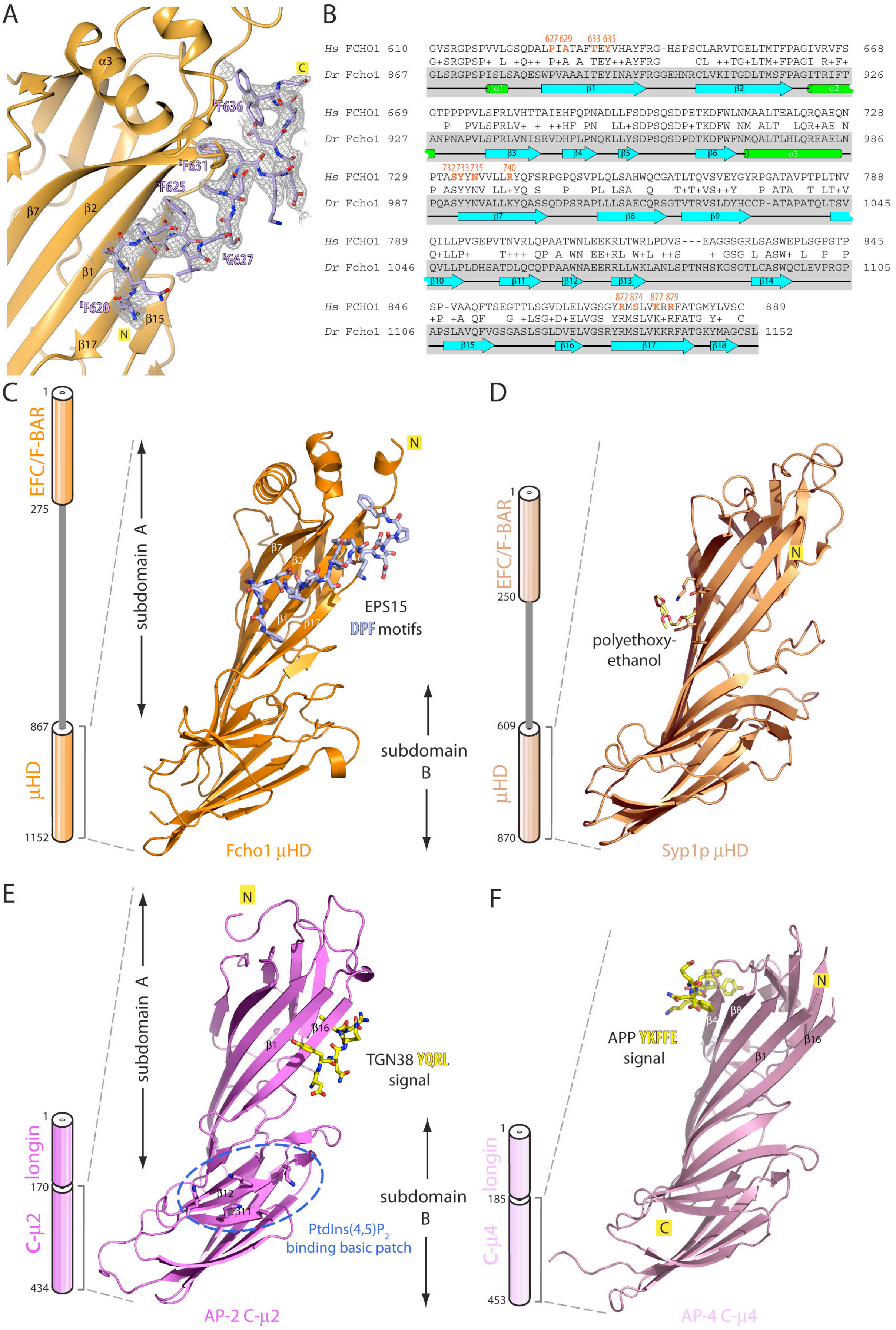
A
α3
C
^{E}F636
^{E}F631
^{E}F625
β7
β2
β1
^{E}G627
^{E}F620
β15
N
β17
B
627 629 633 635
Hs FCHO1 610 GVSRGPSPVVLGSQDALPIATAFTEYVHAYFRG-HSPSCLARVTGELTMTFPAGIVRVFS 668
G+SRGPSP+ L +Q++ P+A A TEY++AYFRG CL ++TG+LTM+FPAGI R+F+
Dr Fcho1 867 GLSRGPSPISLSAQESWPVAAAITEYINAYFRGGEHNRCLVKITGDLTMSFPAGITRIFT 926
α1 β1 β2 α2
Hs FCHO1 669 GTPPPPVLSFRLVHTTAIEHFQPNADLLFSDPSQSDPETKDFWLNMAALTEALQRQAEQN 728
P PVLSFRLV+ + ++HF PN LL+SDPSQSDP+TKDFW NM ALT LQR+AE N
Dr Fcho1 927 ANPNAPVLSFRLVNISRVDHFLPNQKLLYSDPSQSDPDTKDFWFNMQALTLHLQREAELN 986
β3 β4 β5 β6 α3
732 733 735 740
Hs FCHO1 729 PTASYYNVVLLRYQFSRPGPQSVPLQLSAHWQCGATLTQVSVEYGYRPGATAVPTPLTNV 788
P ASYYNV LL+YQ S P PL LSA Q T+T+VS++Y P ATA T LT+V
Dr Fcho1 987 PQASYYNVALLKYQASSQDPSRAPLLLSAECQRSGTVTRVSLDYHCCP-ATAPATQLTSV 1045
β7 β8 β9
Hs FCHO1 789 QILLPVGEPVTNVRLQPAATWNLEEKRLTWRLPDVS---EAGGSGRLSASWEPLSGPSTP 845
Q+LLP+ T+++ QP A WN EE+RL W+L ++S + GSG L ASW+ L P P
Dr Fcho1 1046 QVLLPLDHSATDLQCQPPAAWNAEERRLLWKLANLSPTNHSKGSGTLCASWQCLEVPRGP 1105
β10 β11 β12 β13 β14
872 874 877 879
Hs FCHO1 846 SP-VAAQFTSEGTTLSGVDLELVGSGYRMSLVKRRFATGMYLVSC 889
+P +A QF G +LSG+D+ELVGS YRMSLVK+RFATG Y+ C
Dr Fcho1 1106 APSLAVQFVGSGASLSGLDVELVGSRYRMSLVKKRFATGKYMAGCSL 1152
β15 β16 β17 β18
C
1
EFC/F-BAR
275
867
μHD
1152
subdomain A
N
β7
β2
β1
β17
EPS15
DPF motifs
subdomain B
Fcho1 μHD
D
1
EFC/F-BAR
250
609
μHD
870
N
polyethoxy-
ethanol
Syp1p μHD
E
N
1
longin
170
C-μ2
434
subdomain A
β16
β1
TGN38 YQRL
signal
β12
β11
subdomain B
PtdIns(4,5)P_2
binding basic patch
AP-2 C-μ2
F
N
APP YKFFE
signal
β4
β8
β1
β16
1
longin
185
C-μ4
453
C
AP-4 C-μ4

Figure S2, linked to Figure 3. Fcho1 μHD co-crystal and evolutionary conservation of the concave μHD interaction surface upon subdomain A

A. Electron density map of the EPS15 peptide bound to the zebrafish (*Danio rerio*) Fcho1 μHD. The final refined 2Fo-Fc electron density map contoured at 0.76σ is shown. The interacting EPS15 peptide (mauve) shown in stick representation is superimposed, with nitrogen colored blue and oxygen atoms red. Adjacent is a ribbon representation of a zoomed-in region of subdomain A (orange) from the co-crystal that defines the interaction interface.

B. Primary sequence alignment of the *H. sapiens* (*Hs*) and *D. rerio* (*Dr*) Fcho1 μHDs relative to the secondary structural elements (α-helix, green; β-strand, cyan) present in the *Dr* μHD structure (boxed in gray). The location of the corresponding residues mutated and tested in the *Hs* FCHO1 μHD are shown in orange type.

C. Schematic depiction of the overall domain organization of zebrafish Fcho1 (isoform X1, XM_009303910) alongside a ribbon representation of the backbone of entire Fcho1 μHD. The EPS15 peptide is colored as in A.

D. Domain organization and μHD (PDB ID 3G9H) ribbon diagram for *Saccharomuces cerevisiae* Syp1p. The location of a polyethoxyethanol additive that co-crystallizes with the Syp1p μHD is indicated in yellow sticks (Reider et al., 2009).

E. AP-2 μ2 subunit arrangement and C-μ2 structure (PDB ID 1BXX) ribbon diagram with the bound DYQRLN peptide from TGN38 shown in yellow stick representation (Owen and Evans, 1998). The location of the subdomain B patch with five basic side chains that coordinate a phosphatidylinositol 4,5-bisphosphate (PtdIns(4,5)P_2) head group is shown (blue broken oval).

F. AP-4 μ4 domain organization and C-μ4 (PDB ID 3L81) ribbon diagram, with bound YKFFE peptide from amyloid precursor protein shown in yellow stick representation (Burgos et al., 2010).

Figure S3

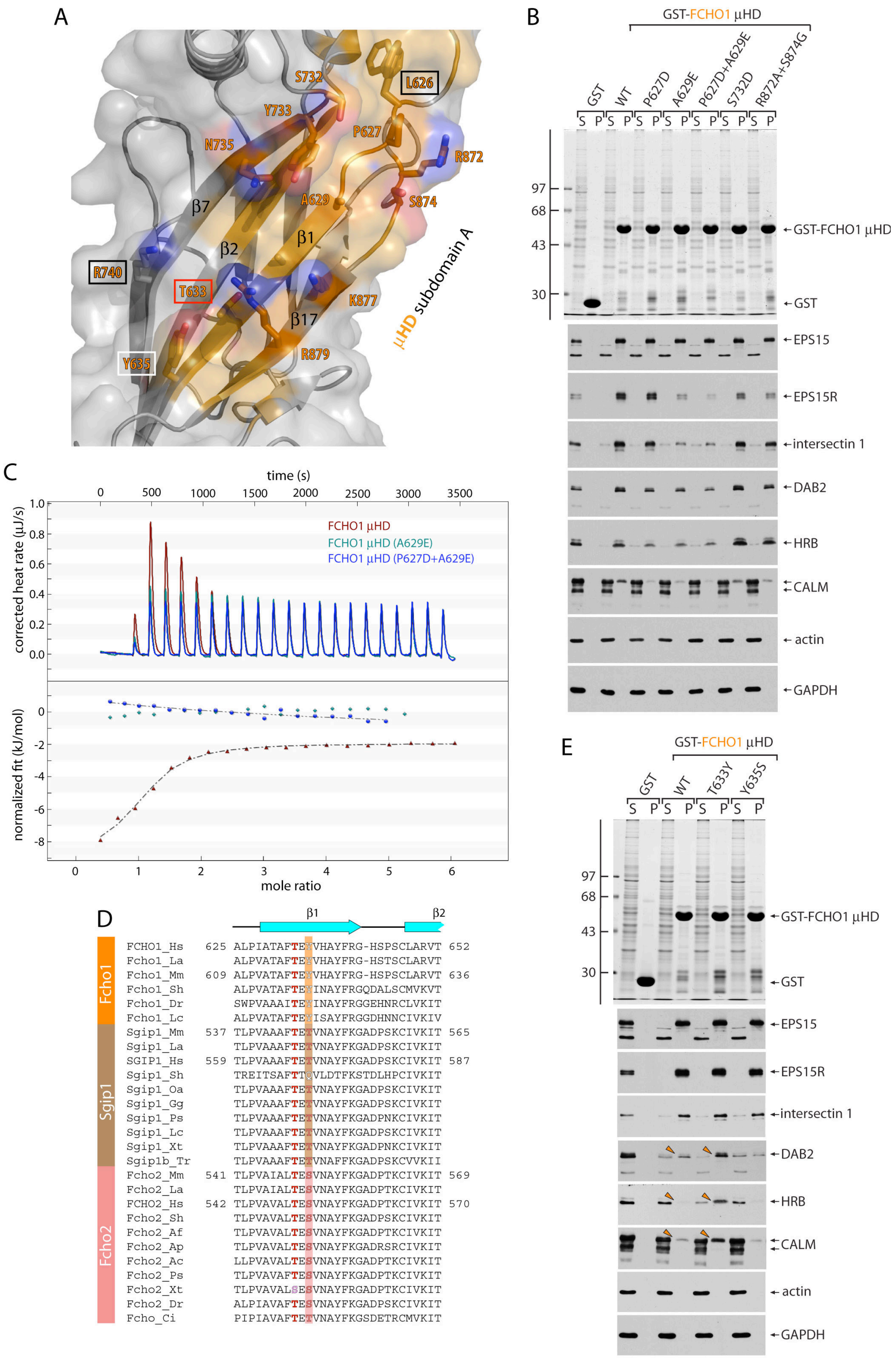
A
S732
L626
Y733
P627
N735
R872
A629
S874
β7
β1
β2
R740
T633
K877
β17
Y635
R879
μHD subdomain A
B
GST-FCHO1 μHD
GST
WT
P627D
A629E
P627D+A629E
S732D
R872A+S874G
S P
97
68
43
30
GST-FCHO1 μHD
GST
EPS15
EPS15R
intersectin 1
DAB2
HRB
CALM
actin
GAPDH
C
time (s)
0
500
1000
1500
2000
2500
3000
3500
corrected heat rate (μJ/s)
FCHO1 μHD
FCHO1 μHD (A629E)
FCHO1 μHD (P627D+A629E)
normalized fit (kJ/mol)
mole ratio
D
β1
β2
Fcho1
Sgip1
Fcho2
FCHO1_Hs 625 ALPIATAFTEYVHAYFRG-HSPSCLARVT 652
Fcho1_La ALPVATAFTEYVHAYFRG-HSTSCLARVT
Fcho1_Mm 609 ALPVATAFTEYVHAYFRG-HSPSCLARVT 636
Fcho1_Sh ALPVATAFTEYINAYFRGQDALSCMVKVT
Fcho1_Dr SWPVAAAITEYINAYFRGGEHNRCLVKIT
Fcho1_Lc ALPVATAFTEYISAYFRGGDHNNCIVKIV
Sgip1_Mm 537 TLPVAAAFTETVNAYFKGADPSKCIVKIT 565
Sgip1_La TLPVAAAFTETVNAYFKGADPSKCIVKIT
SGIP1_Hs 559 TLPVAAAFTETVNAYFKGADPSKCIVKIT 587
Sgip1_Sh TREITSAFTTGVLDTFKSTDLHPCIVKIT
Sgip1_Oa TLPVAAAFTETVNAYFKGADPSKCIVKIT
Sgip1_Gg TLPVAAAFTETVNAYFKGADPSKCIVKIT
Sgip1_Ps TLPVAAAFTETVNAYFKGADPNKCIVKIT
Sgip1_Lc TLPVAAAFTETVNAYFKGADPSKCIVKIT
Sgip1_Xt TLPVAAAFTETVNAYFKGADPNKCIVKIT
Sgip1b_Tr TLPVAAAFTETVNAFFKGADPSKCVVKII
Fcho2_Mm 541 TLPVAIALTESVNAYFKGADPTKCIVKIT 569
Fcho2_La TLPVAIALTESVNAYFKGADPTKCIVKIT
FCHO2_Hs 542 TLPVAVALTESVNAYFKGADPTKCIVKIT 570
Fcho2_Sh TLPVAVALTESVNAYFKGADPSKCIVKIT
Fcho2_Af TLPVAVALTESVNAYFKGADPTKCIVKIT
Fcho2_Ap TLPVAVALTESVNAYFKGADRSRCIVKIT
Fcho2_Ac LLPVAVALTESVNAYFKGADPTKCIVKIT
Fcho2_Ps TLPVAVAFTESVNAYFKGADPTKCIVKIT
Fcho2_Xt TLPVAVALSESVNAYFRGADPTKCIVKIT
Fcho2_Dr ALPIAVAFTESVNAYFKGADPSKCIVKIT
Fcho_Ci PIPIAVAFTETVNAYFKGSDETRCMVKIT
E
GST-FCHO1 μHD
GST
WT
T633Y
Y635S
S P
97
68
43
30
GST-FCHO1 μHD
GST
EPS15
EPS15R
intersectin 1
DAB2
HRB
CALM
actin
GAPDH

Figure S3, linked to Figure 3. Additional characterization of structure-guided mutations in FCHO1 μHD subdomain A

A. Combined ribbon and molecular surface representation of the *D. rerio* Fcho1 μHD with numbering of the corresponding *H. sapiens* FCHO1 μHD residues shown. Differing side chains are boxed in black, while the TXY residues in strand β1 at the base of the binding trough are boxed either in red (conserved in muniscins) or white (not conserved in Fcho2 or Sgip1), respectively. The location of side chains examined by mutagenesis is shown in stick representation with nitrogen colored blue and oxygen red.

B. GST pull-down assays with HeLa cell lysate and 125 μg of GST or various GST-FCHO1 μHD fusions as indicated. Separated supernatant (2%) and pellet (10%) fractions were either stained or replicates immunoblotted with the indicated antibodies. Notice that for this set of mutations, EPS15, EPS15R, intersectin 1, DAB2 and HRB are not recovered in the supernatant fractions, as is the case with the K877E+R879A mutation (Figure 3E-F). This indicates that these mutations to the helical region of subdomain A primarily affect the off-rate of the interactions with the μHD.

C. Representative ITC experiments with the wild-type *Hs* FCHO1 μHD or (A629E)- or (P627D+A629E)-substituted FCHO1 μHDs titrated with the EPS15 616-638 peptide. Titrations were performed at 4°C due to thermal instability and aggregation of these mutants upon prolonged stirring at 12°C.

D. Alignment of stand β1 amino acid regions from muniscin family members of selected organisms (genus/species abbreviations: Hs, *Homo sapiens*; La, *Loxodonta africana* (African bush elephant); Mm, *Mus musculus*; Sh; *Sarcophilus harrisii* (Tasmanian devil); Dr, *Danio rerio*; Lc, *Latimeria chalumnae* (coelacanth); Oa, *Ornithorhynchus anatinus* (duck-billed platypus); Gg, *Gallus gallus*; Ps, *Pelodiscus sinensis* (Chinese softshell turtle); Xt, *Xenopus tropicalis*; Tr, *Takifugu rubripes* (Japanese pufferfish); Af, *Aptenodytes forsteri* (emperor penguin); Ap, *Anas platyrhynchos* (domestic duck); Ac, *Anolis carolinensis* (green anole lizard); Ci, *Ciona intestinalis* (sea squirt)) are shown with appropriate residues numbers indicated. Identical residues are highlighted in red, highly similar residues in pale pink.

E. Pull-down assay with HeLa cell lysate and the indicated amount of GST, GST-FCHO1 μHD mutants immobilized on glutathione-Sepharose. Separated supernatant (2%) and pellet (10%) fractions were either stained or replicates immunoblotted with the indicated antibodies. The apparent gain-of-function effect of the T633Y substitution on the weaker binding partners is highlighted in comparison with the wild-type FCHO1 μHD (arrowheads).

Figure S4

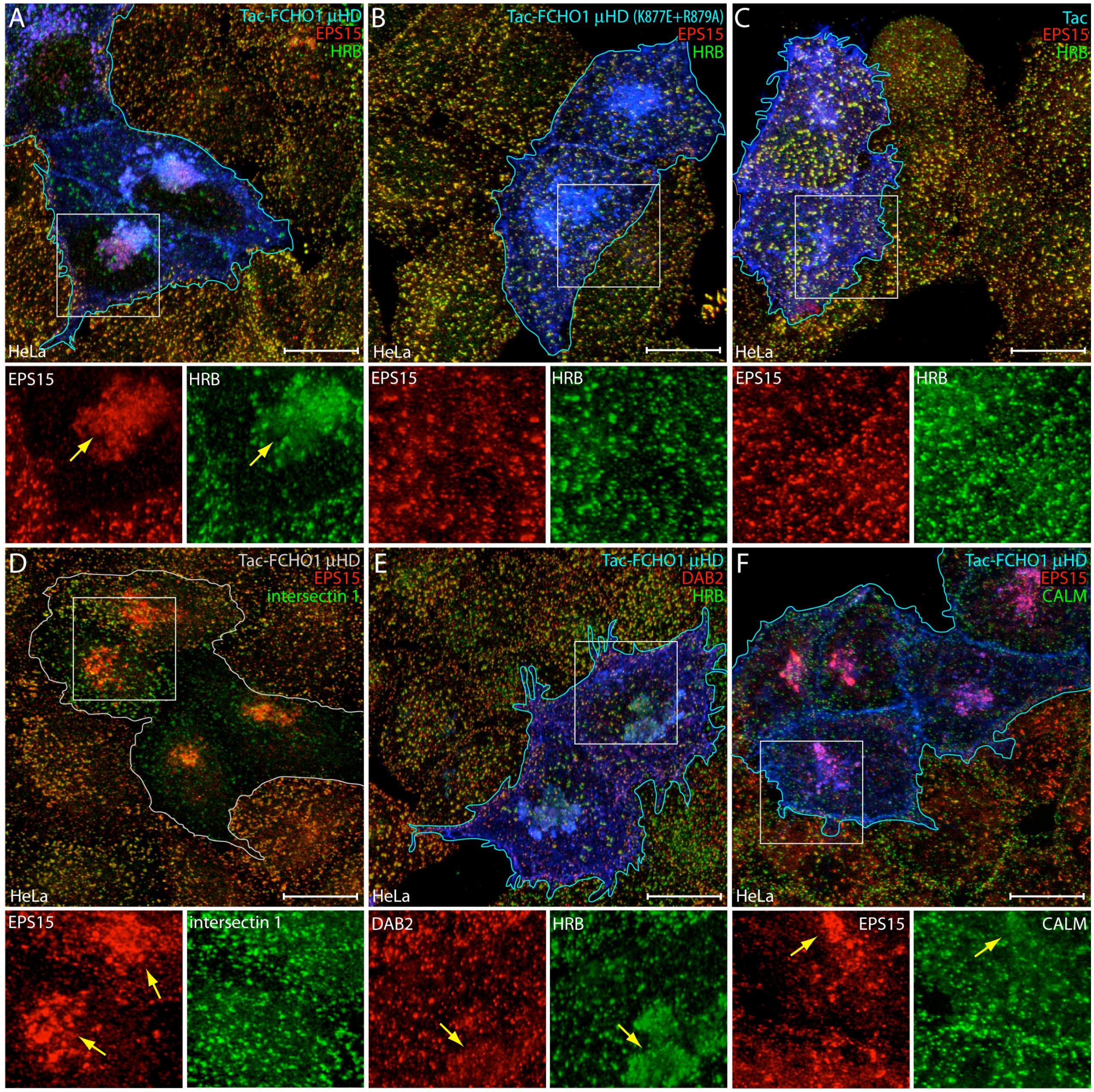

A
Tac-FCHO1 μHD
EPS15
HRB
HeLa
EPS15
HRB
B
Tac-FCHO1 μHD (K877E+R879A)
EPS15
HRB
HeLa
EPS15
HRB
C
Tac
EPS15
HRB
HeLa
EPS15
HRB
D
Tac-FCHO1 μHD
EPS15
intersectin 1
HeLa
EPS15
intersectin 1
E
Tac-FCHO1 μHD
DAB2
HRB
HeLa
DAB2
HRB
F
Tac-FCHO1 μHD
EPS15
CALM
HeLa
EPS15
CALM

Figure S4, linked to Figure 4. Tac-μHD lures only select endocytic pioneers/CLASPs onto intracellular compartments

A-C. Typical maximal intensity projections of confocal image *z* stacks from fixed HeLa cells transiently expressing Tac (E) or Tac-μHD (K877E+R819A) (F) and stained with antibodies against EPS15, HRB and Tac (transfected cell groups outlined in blue) as indicated. Enlarged, color-separated channels of the boxed regions are shown below. Unlike the Tac-μHD, little intracellular relocation of EPS15 or HRB (arrows) is apparent on overexpression of either Tac or the Tac-μHD (K877E+R879A) mutant.

D-F. Representative maximal intensity projections of deconvolved confocal image *z* stacks of HeLa SS6 cells transiently transfected with Tac-FCHO1 μHD. Fixed cells were stained with anti-EPS15, anti-intersectin 1, anti-Dab2, anti-HRB, anti-CALM and anti-Tac antibodies as indicated, Pronounced movement of EPS15 away from the surface (arrows) on expression of the wild-type μHD fused to the Tac C-terminus is not accompanied by equivalent repositioning of all μHD partners. Note that intersectin 1 resists prominent relocation onto intracellular membranes in parallel to EPS15, which identifies the Tac-μHD-transfected cells (gray outline) in absence of anti-Tac staining. Sale bar = 10 mm.

Figure S5

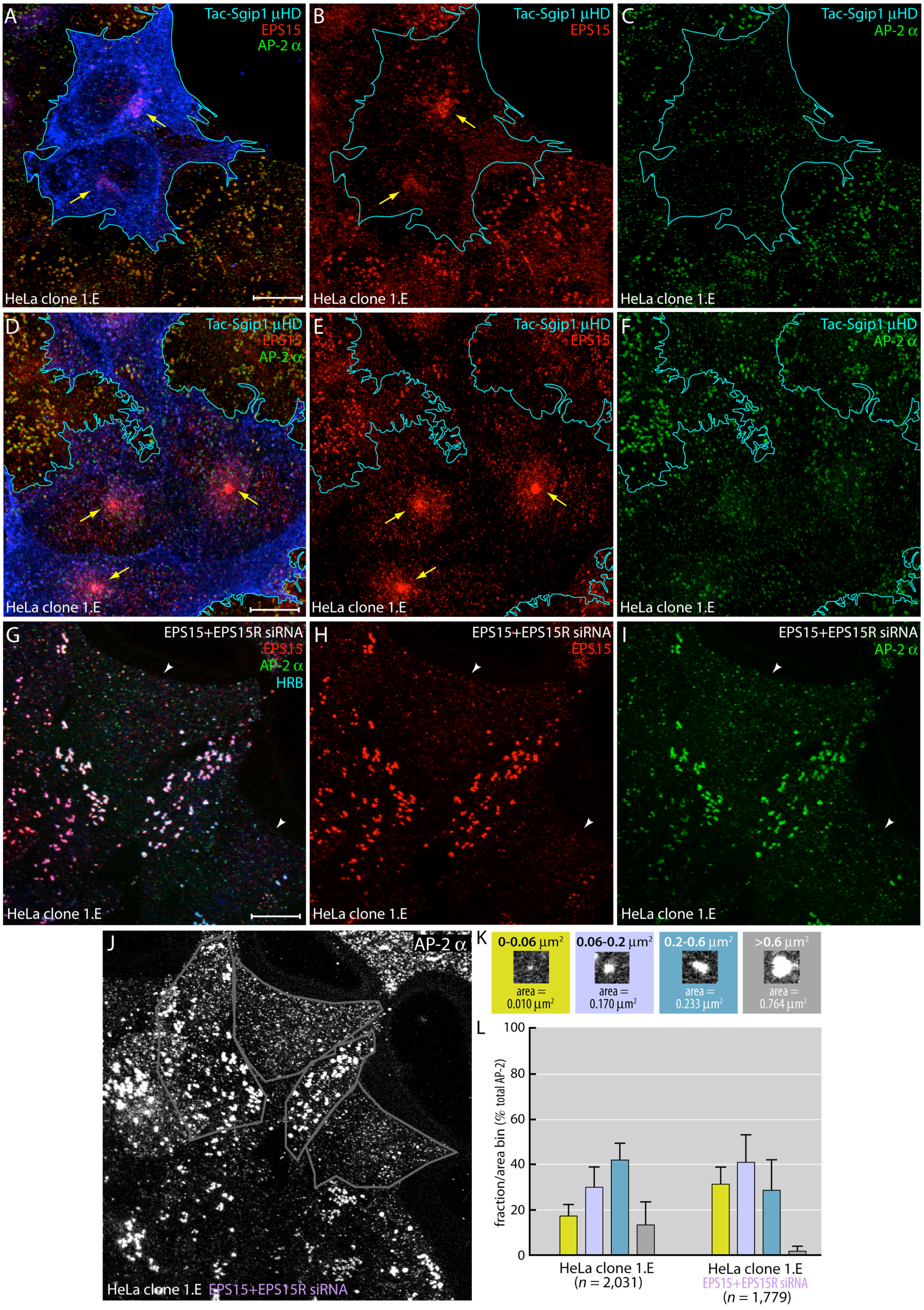
A
Tac-Sgip1 µHD
EPS15
AP-2 α
HeLa clone 1.E
B
Tac-Sgip1 µHD
EPS15
HeLa clone 1.E
C
Tac-Sgip1 µHD
AP-2 α
HeLa clone 1.E
D
Tac-Sgip1 µHD
EPS15
AP-2 α
HeLa clone 1.E
E
Tac-Sgip1 µHD
EPS15
HeLa clone 1.E
F
Tac-Sgip1 µHD
AP-2 α
HeLa clone 1.E
G
EPS15+EPS15R siRNA
EPS15
AP-2 α
HRB
HeLa clone 1.E
H
EPS15+EPS15R siRNA
EPS15
HeLa clone 1.E
I
EPS15+EPS15R siRNA
AP-2 α
HeLa clone 1.E
J
AP-2 α
HeLa clone 1.E EPS15+EPS15R siRNA
K
0-0.06 µm²
area = 0.010 µm²
0.06-0.2 µm²
area = 0.170 µm²
0.2-0.6 µm²
area = 0.233 µm²
>0.6 µm²
area = 0.764 µm²
L
fraction/area bin (% total AP-2)
100
80
60
40
20
0
HeLa clone 1.E
(n = 2,031)
HeLa clone 1.E
EPS15+EPS15R siRNA
(n = 1,779)

Figure S5, linked to Figures 4 and 5. Remodeling of cell-surface AP-2 puncta in presence of ectopic Tac-Sgip1 μHD expression or after extinguishing EPS15 and EPS15R with transfected siRNA SMARTpools

A-F. Two selected but representative maximal-projection images of deconvoluted confocal *z* stacks from HeLa clone 1.E cells transiently transfected a Tac-Sgip1 μHD plasmid. Fixed and permeabilized cells were stained with antibodies against EPS15, the α subunit of AP-2 and Tac (transfected cell groups outlined in blue), as indicated. Separated color-channels (B, C and E, F) are shown to highlight the change in AP-2 puncta when EPS15 is redirected to the cell interior with the Tac-Sgip1 μHD chimera. Scale bar = 10 μm.

G-I. HeLa clone 1.E cells were transfected twice sequentially with SMARTpools of siRNA oligonucleotides targeting both the *EPS15* and *EPS15L1* (EPS15R) transcripts. Fixed and permeabilized cells were labeled with antibodies raised against EPS15, the α subunit of AP-2 and HRB. A maximal projection of deconvolved *z* stacks is shown, with cells displaying minimal EPS15 signal and a corresponding alteration in the AP-2 distribution indicated with arrowheads. The changed AP-2 morphology is consistent with the phenotype observed with Tac-μHD expression in these cells. Note that in these experiments, we unfortunately cannot determine the expression level of EPS15R in individual cells due to a lack of a suitable antibody for immunofluorescence analysis. Scale bar = 10 μm.

J-L. Quantitative analysis of area distributions of AP-2 assemblies in EPS15 + EPS15R-silenced HeLa clone 1.E cells. A representative gray scale image of AP-2 α-subunit staining (J), examples of AP-2-positive puncta within the color-coded area bins selected (K), and puncta area distributions and standard deviations in EPS15 positive (clone 1.E) and siRNA-silenced EPS15-deficient cells (L) are shown.

Figure S6

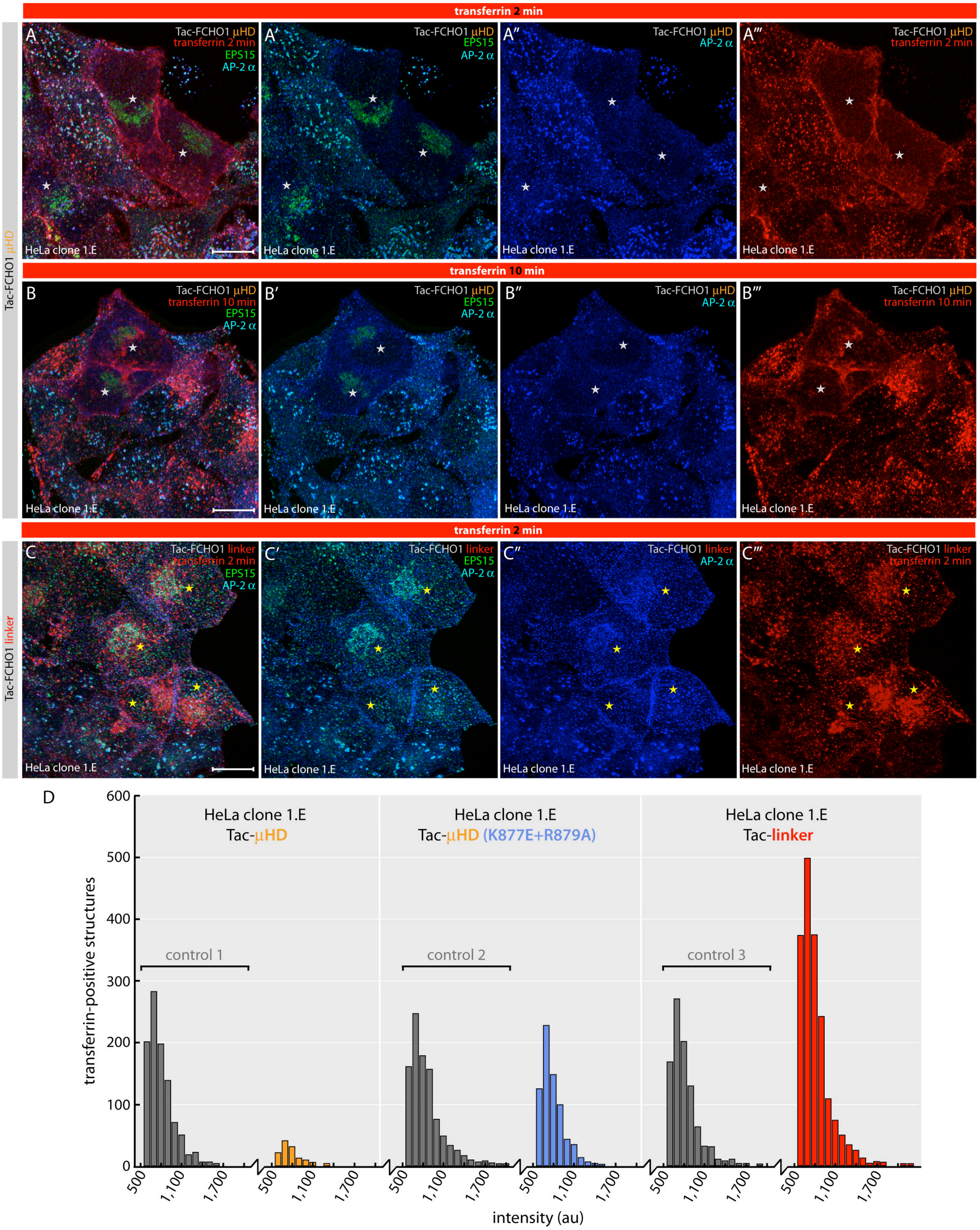

transferrin 2 min
transferrin 10 min
Tac-FCHO1 µHD
Tac-FCHO1 linker
A
A′
A″
A‴
B
B′
B″
B‴
C
C′
C″
C‴
Tac-FCHO1 µHD
transferrin 2 min
transferrin 10 min
Tac-FCHO1 linker
EPS15
AP-2 α
HeLa clone 1.E
D
HeLa clone 1.E
Tac-µHD
Tac-µHD (K877E+R879A)
Tac-linker
control 1
control 2
control 3
transferrin-positive structures
intensity (au)
0
100
200
300
400
500
600
500
1,100
1,700

Figure S6, linked to Figure 5. Defective CME in FCHO1/2-null HeLa 1.E cells expressing Tac-μHD

A-C. Selected but representative maximal intensity projection images of deconvoluted confocal *z* stacks from HeLa clone 1.E cells transiently transfected with either the Tac-FCHO1 μHD (A-B''') or the Tac-FCHO1 linker (C-C''') plasmid. Serum-starved cells were pulsed with Alexa Fluor 546 transferrin for 2 (A and C) or 10 (B) min before fixation. Permeabilized cells were stained with antibodies directed against EPS15 and the AP-2 α subunit as indicated. Composite images (A-C), dual color EPS15 and AP-2 α subunit (A'-C') as well as individual AP-2 α subunit (A''-C'') or transferrin (A'''-C''') color channels are shown. Tac-μHD-expressing cells are identified by prominent accumulation of EPS15 in the Golgi region (white asterisks) and by loss of the phenotypical enlarged CCSs on the ventral plasma membrane surface. These attributes correlate with the changed AP-2 distribution in the cells. By contrast, Tac-FCHO1 linker expressing 1.E cells have a different alteration to the steady-state distribution of AP-2, which is correlated with increased transferrin internalization (yellow asterisks). Scale bar = 10 μm.

D. Histograms of quantified data from transferrin pulse-chase uptake experiments performed as described in Figure 5. The total number of transferrin-positive internalized puncta (after a two minute chase at 37°C) counted from three or four separate confocal data sets are: HeLa clone 1.E cell control 1 = 980, Tac-μHD-expressing cells = 113; HeLa clone 1.E cell control 2 = 953, Tac-μHD (K877E+R879A)-expressing cells = 690; HeLa clone 1.E cell control 3 = 914, Tac-linker-expressing cells = 1,785, and the same aggregate total area (2,443.4 μm^2) was analyzed for each population.

Figure S7

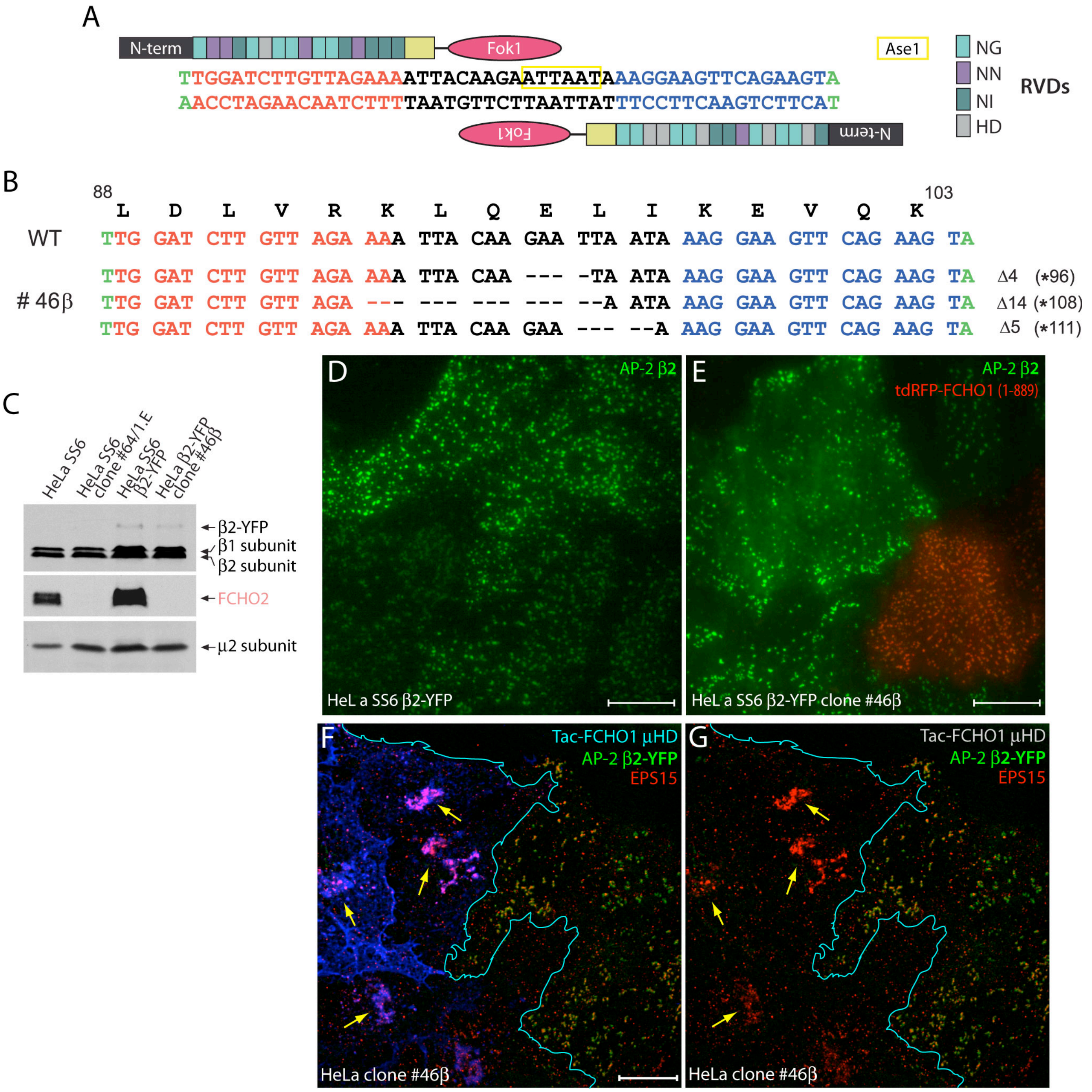
A
N-term
Fok1
Ase1
TTGGATCTTGTTAGAAAATTACAAGAATTAATAAAGGAAGTTCAGAAGTA
AACCTAGAACAATCTTTTAATGTTCTTAATTATTTCCTTCAAGTCTTCAT
NG
NN
NI
HD
RVDs
B
88
103
L D L V R K L Q E L I K E V Q K
WT
TTG GAT CTT GTT AGA AAA TTA CAA GAA TTA ATA AAG GAA GTT CAG AAG TA
46β
TTG GAT CTT GTT AGA AAA TTA CAA --- -TA ATA AAG GAA GTT CAG AAG TA Δ4 (*96)
TTG GAT CTT GTT AGA --- --- --- --- --A ATA AAG GAA GTT CAG AAG TA Δ14 (*108)
TTG GAT CTT GTT AGA AAA TTA CAA GAA --- --A AAG GAA GTT CAG AAG TA Δ5 (*111)
C
HeLa SS6
HeLa SS6 clone #64/1.E
HeLa SS6 β2-YFP
HeLa β2-YFP clone #46β
β2-YFP
β1 subunit
β2 subunit
FCHO2
μ2 subunit
D
AP-2 β2
HeL a SS6 β2-YFP
E
AP-2 β2
tdRFP-FCHO1 (1-889)
HeL a SS6 β2-YFP clone #46β
F
Tac-FCHO1 μHD
AP-2 β2-YFP
EPS15
HeLa clone #46β
G
Tac-FCHO1 μHD
AP-2 β2-YFP
EPS15
HeLa clone #46β

Figure S7, linked to Figure 6. Characterization of the HeLa SS6 β2-YFP clone #46β FCHO2-null line

A. Design strategy for an exon 4-targeting TALEN pair mediated disruption of the *FCHO2* locus on chromosome 5 (Umasankar et al., 2014). The different repeat variable dipeptides (RVD) are color coded as indicated. The endogenous internal AseI restriction site used for screening is boxed (yellow).

B. Genotype of clone #46β cells compared with the wild-type (WT) sequence and encoded amino acid sequence above. The * symbol represents an in-frame stop codon at the indicated residue position. Unlike the parental HeLa SS6 cells (Umasankar et al., 2014), the β2-YFP stable line derived therefrom is clearly aneuploid, with three copies of chromosome 5. Two other independent clones (#5β and #52β) similarly have three (differently) disrupted alleles of the *FCHO2* locus.

C. Immunoblot comparison of HeLa SS6 parental cells, the clone#64/1.E FCHO1+FCHO2-null cells, the HeLa SS6 β2-YFP stable cell line, and the clone #46β FCHO2 null β2-YFP expressing line. Note β2-YFP expression levels in these cells are substoichiometric with the endogenous β1 and β2 adaptor subunits.

D. Selected but representative TIRFM images of the adherent ventral surface of HeLa SS6 β2-YFP (D) and clone #46β (E) cells. Note the altered morphology of β2-YFP-positive CCSs in the clone #46β cells that closely resembles that seen in HeLa SS6 clone #64/1.E cells, and that is corrected by transient expression of tdRFP-FCHO1 (1-889). Scale bar = 10 μm.

F. Redistribution of endogenous EPS15 in the HeLa clone #46β FCHO2-null β2-YFP expressing line after ectopic expression of Tac-FCHO1 μHD. A typical maximal intensity projection of deconvolved *z* stacks from fixed and permeabilized HeLa clone #46β cells stained with antibodies directed against EPS15 and Tac (transfected cell groups outlined in blue) as indicated. The conspicupous juxtanuclear sequestration of EPS15 (arrows) away from the enlarged clathrin-coated structures at the cell surface is indicated. Scale bar = 10 μm.

Table S1, linked to Figure 3. Data collection and refinement statistics

	**Native EPS15-Fcho1 μHD chimera**	**Xe derivative EPS15-Fcho1 μHD chimera**
**Data Collection**		
Beamline	Diamond IO4-1	Diamond IO2
Space group	H3	H3
Wavelength (Å)	0.92 Å	1.6 Å
**Cell dimensions (Å)**		
a, b, c (Å)	239.8, 239.8, 44.1	236.5, 236.5, 44.0
α, β, γ (°)	90, 90, 120	90, 90, 120
Resolution range (Å)[a]	45.31-2.40 (2.49-2.40)	118.27-2.79 (2.96-2.79)
R_{merge}[ab]	0.80 (1.236)	0.253 (3.80)
R_{meas}[ac]	0.112 (1.745)	0.274 (4.301)
$CC_{1/2}$[ad]	0.996 (0.246)	0.996 (0.243)
Mean $I/\sigma I$[a]	8.3 (1.0)	10.4 (0.8)
Completeness (%)[a]	96.0 (99.1)	99.4 (96.0)
Multiplicity[a]	2.9 (2.9)	13.3 (8.8)
Wilson <B> (Å^2)		
Anomolous completeness (%)[a]	83.7 (85.8)	99.3 (95.7)
$CC_{1/2}$ on Δ_{anom} (inner bin)[a]	-0.078 (-0.109)	0.041 (0.257)
**Phasing (autosol)**		
Number of sites		14
Mean figure of merit		0.229
BAYES-CC		38.4
R-factor after density modification		0.29
Map skew after density modification		0.11
**Refinement**		
Resolution range (Å)	43.3-2.4	
No. of reflections	35451	
R_{work}/R_{free}	0.199/0.234	
Number of atoms	4596	
Protein	4335	
Ligand/ion	1	
Waters	40	
B-factors (Å^2)	58.4	
**Rmsd from ideal values**		
Bond lengths (Å)	0.01	
Bond angles (°)	0.92	
**Ramachandran Plot**		
Favoured region (%)	98.8	
Allowed (%)	1.2	
Outliers (%)	0	
Rotamer outliers (%)	2	
C-beta outliers	2.89	

[a] Values in parentheses are for the highest resolution shell. [b] $R_{merge} = \Sigma(I_{hl} - <I_h>)/\Sigma(I_{hl})$ where $<I_h>$ is the mean intensity of unique reflection *h*, summed over all reflections for each observed intensity I_{hl}. [c] $R_{meas} = \Sigma(n/n - 1)^{1/2} (I_{hl} - <I_h>)/\Sigma(I_{hl})$ where *n* is the number of observations for unique reflection *h* with mean intensity $<I_h>$, summed over all reflections for each observed intensity I_{hl}. [d] $CC_{1/2}$ is the correlation coefficient on $<I>$ between random halves of the dataset. Δ_{anom}, anomalous difference $I^+ - I^-$.

Supplemental Experimental Procedures

Materials

Fetal calf serum (FCS) was obtained from Atlanta Biologicals and paraformaldehyde was from Electron Microscopy Sciences. Frozen unstripped rat brain tissue was from PelFreez. Tissue culture medium, trypsin and additives were purchased from Lonza or Mediatech. Alexa Fluor 488, -546 and -647 conjugated transferrin (Tf) was from Invitrogen. Antibiotics, IPTG and DTT were obtained from Gold Biotechnology. Acrylamide, benzamidine, bis-acrylamide, Coomassie blue R250, Tris, Hepes, imidazole, EDTA, EGTA, ammonium persulfate, TEMED, B-PER and Y-PER, LB medium, 2 × YT medium, Lipofectamine 2000, Oligofectamine and a 10 × concentrated PBS stock from Thermo Fisher, benzonase, lysozyme, fatty-acid free BSA, PMSF, reduced glutathione, fish skin gelatin, saponin, Triton X-100 and Ponceau S from Sigma, and cOmplete protease inhibitor tablets from Roche Applied Science. Ni-NTA-agarose was purchased from McLab, Bradford reagent and SDS was from Biorad. Protein molecular mass standards, glutathione-Sepharose and nitrocellulose were purchased from GE Healthcare, and synthetic EPS15 peptides were obtained from Genscript. All DNA primers were synthesized by Integrated DNA Technologies (IDT) and all dideoxynucleotide sequencing was performed by Genewiz using commercial sequencing primers.

Antibodies

Antigen	Species	Designation/ clone	Catalogue number	Supplier/source
β-actin	mouse	mAb C4	sc-47778	Santa Cruz Biotechnology
AP-2 α subunit	mouse	mAb C-8	sc-17771	Santa Cruz Biotechnology
AP-2 α subunit	mouse	mAb AP.6	—	Frances Brodsky
AP-1/2 β1/β2 subunit	rabbit	antigen affinity purified pAb GD/1	—	Traub laboratory
AP-1/2 β1/β2 subunit	mouse	mAb 100/1	—	Ernst Ungewickell
AP-2 μ2 subunit	rabbit	R11-29 serum	—	Juan Bonifacino
CALM	goat	affinity purified pAb C-18	sc-6433	Santa Cruz Biotechnology
CALM/PICALM	rabbit	mAb ab172962	EPR12177	AbCAM
clathrin heavy chain	mouse	mAb TD.1	—	Frances Brodsky
Dab2	rabbit	antigen affinity purified pAb	—	Traub laboratory
Dab2	rabbit	mAb D709T	12906	Cell Signaling Technology
Disabled-2/p96	mouse	mAb 52/p96	610464	BD Transduction Laboratories
Eps15	rabbit	affinity purified pAb C-20	sc-534	Santa Cruz Biotechnology
Eps15	rabbit	antigen affinity purified pAb	—	Ernst Ungewickell
Eps15	rabbit	mAb D3K8R	12460	Cell Signaling Technology
Eps15R	rabbit	mAb ab53006	EP1147Y	AbCAM
Epsin 1	rabbit	antigen affinity purified pAb	—	Traub laboratory
FCHO1	rabbit	antigen affinity	—	Traub laboratory

		purified pAb		
FCHO1	mouse	mAb G-7	sc-365043	Santa Cruz Biotechnology
FCHO2	rabbit	antigen affinity purified pAb	NBP2-32694	Novus Biologicals
GAPDH	mouse	mAb	Y1041	UBP Bio
GFP	rabbit	antigen affinity purified pAb B5	—	Phyllis Hanson
GPP130	rabbit	serum	—	Adam Linstedt
Hrb/RIP/Rab	goat	affinity purified pAb C-19	sc-1424	Santa Cruz Biotechnology
Hrb/AGFG1	mouse	mAb H-2	sc-166651	Santa Cruz Biotechnology
Intersectin 1	mouse	mAb clone 29	611574	BD Transduction Laboratories
PKCα	mouse	mAb MC5	sc-80	Santa Cruz Biotechnology
SHIP2	mouse	mAb E-2	sc-166641	Santa Cruz Biotechnology
Tac (CD25)	mouse	mAb 7G7B6	174-020	Ancell
Tac (CD25)	goat	antigen affinity-purified pAb	AF-223-NA	R & D Systems

Plasmids, DNA manipulations and RNAi oligonucleotides

We used *Homo sapiens* (*Hs*) FCHO1 μHD, *Danio rerio* (*Dr*) Fcho1, *Hs* FCHO2 μHD, *Hs* EPS15 and *Mus musculus* (*Mm*) Sgip1 μHD cDNAs (Reider et al., 2009; Umasankar et al., 2014; Umasankar et al., 2012). GST was produced from the pGEX-4T-1 plasmid and all GST-fusion proteins were constructed in this parental vector. GST-AP-2 α_C appendage (GST-α_C) (Traub et al., 1999) and GST-FCHO1 μHD, GST-FCHO2 μHD, GST-Sgip1 μHD, GST-EPS15 (595-740), (595-660), (595-636), (595-620) and GST-EPS15 (595-896)-His_{12} have been described previously (Umasankar et al., 2014; Umasankar et al., 2012). *Hs* EPS15 residues 615-636 were excised from GST-EPS15 (595-636) plasmid using an internal EcoRI restriction site and re-cloned into the same vector to create GST-EPS15 (615-636). Similarly, GST-EPS15 (626-639) was obtained by annealing appropriate oligonucleotide pairs between EcoRI and XhoI sites in the parent vector. Addition of synthetic GAGA linker in-frame of *Dr* Fcho1 μHD toward the N-terminus yielded GST-GAGA-*Dr* Fcho1 μHD. Subsequent insertion of a *Hs* EPS15 (615-637) amplicon, further upstream, between BamHI and EcoRI sites in the same vector created GST-EPS15-μHD. Construction of GFP-FCHO1 (1-889), GFP-FCHO1 (1-609), Tac-FCHO1 μHD, and Tac-FCHO1 linker have been explained elsewhere (Umasankar et al., 2014; Umasankar et al., 2012). Full-length *Hs* FCHO1 (1-889), *Hs* FCHO1 μHD (609-889) and *Hs* EPS15 (1-896) were PCR amplified and cloned in frame following tdRFP in the ptdTomato-C1 vector. The GFP-FYVE-FCHO1 μHD was cloned into the GFP-(2 × Hrs)FYVE-UbΔGG plasmid from Pietro De Camilli (Chen and De Camilli, 2005) by replacing the C-terminal ubiquitin with an EcoRI-NotI digested FCHO1 μHD PCR product. Routine PCR amplification typically used PfuTurbo DNA polymerase (Agilent Technologies). Two methods for DNA modification were used. Site-directed mutagenesis most often was with the QuikChange system (Invitrogen), utilizing primer pairs (Table S3) that incorporated the necessary mutations mentioned in the text. The Phusion HT (Thermo Fisher) PCR procedure was used to remove or add additional sequences to existing plasmid templates using the appropriate 5'-phosphorylated primer sets. Detailed information on the plasmids, cDNA inserts and cloning primers are provided below:

Gene product/ Protein	Plasmid	Oligo sequence	Restriction sites	Notes
GST-FCHO1 μHD	pGEX-4T-1	5' ATA GAA TTC CAC GGA GTC TCC CGG G 3'<br>5' GCG TCG ACT CAG CAG CTC ACC AGG TAC 3'	EcoRI/SalI	Directional cloning
GST-FCHO2 μHD	pGEX-4T-1	5' GCG GAT CCG GTG TGT CAC GGG GTC CCA GC 3'<br>5' ATG CGG CCG CTC AAC AAT CCG CCA GGT ATC G 3'	EcoRI/NotI	Directional cloning
GST-*Mm* Sgip1 μHD	pGEX-4T-1	5' TCG GAT CCA CTC CCA CAG TTG GTT CC 3'<br>5' AAG CGG CCG CTT AGT TAT CTG CCA AGT AC 3'	BamHI/NotI	Directional cloning
GST-EPS15 (595-740)	pGEX-4T-1	5' GAA GAT CCT TTT CGT TGA GCC ACA TCG AGC TCT G 3'<br>5' CAG AGC TCG ATG TGG CTC AAC GAA AAG GAT CTT C 3'		QuikChange mutagenesis
GST-EPS15 (595-660)	pGEX-4T-1	5' GAC TGT TTC TTC AGG TAA TCT ACT GAT CCT TTT GCC 3'<br>5' GGC AAA AGG ATC AGT AGA TTA CCT GAA GAA ACA GTC 3'		QuikChange mutagenesis
GST-EPS15 (595-636)	pGEX-4T-1	5' CTT TCA AGG ATG ATC CTT TTT GAA AAA TCG ATC CAT TTG GTG G 3'<br>5' CCA CCA AAT GGA TCG ATT TTT CAA AAA GGA TCA TCC TTG AAA G 3'		QuikChange mutagenesis
GST-EPS15 (595-620)	pGEX-4T-1	5' GAT ACA AAC TTG GAT TTT TTC TAG TCT GAT CCT TTT GTT GGC AG 3'<br>5' CTG CCA ACA AAA GGA TCA GAC TAG AAA AAA TCC AAG TTT GTA TC 3'		QuikChange mutagenesis
GST-EPS15 (626-639)	pGEX-4T-1	5' AAT TCG TTG GCA GTG ATC CTT TCA AGG ATG ATC CTT TTG GAA AAA TCC 3'<br>5' TCG AGG ATT TTT CCA AAA GGA TCA TCC TTG AAA GGA TCA CTG CCA ACG 3'	EcoRI/XhoI	Directional cloning
GST-EPS15 (615-636)	pGEX-4T-1	5' GCT GAC AGG TCC AGT TGC AGA ATT CAC AAA CTT GGA TTT TTT CCA G 3'<br>5' CTG GAA AAA ATC CAA GTT TGT GAA TTC TGC AAC TGG ACC TGT CAG C 3'		QuikChange mutagenesis
GST-EPS15 (615-636) ^{623}DPF→APA	pGEX-4T-1	5' GGA TTT TTT CCA GTC TGC TCC TGC TGT TGG CAG TGA TCC TTT C 3'<br>5' GAA AGG ATC ACT GCC AAC AGC AGG AGC AGA CTG GAA AAA ATC C 3'		QuikChange mutagenesis
GST-*Dr* Fcho1 μHD	pGEX-4T-1	5' GTG AAT TCG GTC TGT CTC GAG GAC CCA G 3'<br>5' ATG CGG CCG CTC ATA AAG AAC AGC CGG CCA TG 3'	EcoRI/NotI	Directional cloning
GST-*Hs* EPS15-*Dr* Fcho1 μHD	pGEX-4T-1	5' TAT GGA TCC ACA AAC TTG GAT TTT TTC CAG TC 3'<br>5' CGT GAA TTC TCC AAA AGG ATC ATC CTT G 3'	BamHI/EcoRI	Directional cloning
GST-*Hs* EPS15-GAGA-*Dr* Fcho1 μHD	pGEX-4T-1	5' TTC AAG GAT GAT CCT TTT GGA GGC GCT GGA GCA GGT CTG TCT CGA GGA CCC AG 3'<br>5' CTG GGT CCT CGA GAC AGA CCT GCT CCA GCG CCT CCA AAA GGA TCA TCC TTG AA 3'		QuikChange mutagenesis
GST-ΔEPS15-μHD	pGEX-4T-1	5'-/5Phos/GTT GGC AGT GAT CCT TTC AAG GAT GAT C 3'<br>5'-/5Phos/GGA TCC ACG CGG AAC CAG ATC 3'		Phusion PCR
GST-EPS15 [APA]-μHD	pGEX-4T-1	5' GGA TTT TTT CCA GTC TGC TCC TGC TGT TGG CAG TGA TCC TTT C 3'<br>5' GAA AGG ATC ACT GCC AAC AGC AGG AGC AGA CTG GAA AAA ATC C 3'		QuikChange mutagenesis
GST-FCHO1 μHD (K877E)	pGEX-4T-1	5' CAT GTC GCT GGT GGA GAG GAG GTT TGC C 3'<br>5' GGC AAA CCT CCT CTC CAC CAG CGA CAT G 3'		QuikChange mutagenesis
GST-FCHO1 μHD (K877E + R879A)	pGEX-4T-1	5' CGC ATG TCG CTG GTG GAG AGG GCG TTT GCC ACA GGG ATG TAC CTG 3'<br>5' CAG GTA CAT CCC TGT GGC AAA CGC CCT CTC CAC CAG CGA CAT GCG 3'		QuikChange mutagenesis
GST-FCHO2 μHD (K798E)	pGEX-4T-1	5' ATA GGC TTT CCT TAA TAG AGA AGC GGT TTG CTA CTG G 3'		QuikChange [illegible]

GST-FCHO2 μHD (K798E)	pGEX-4T-1	5' ATA GGC TTT CCT TAA TAG AGA AGC GGT TTG CTA CTG G 3'<br>5' CCA GTA GCA AAC CGC TTC TCT ATT AAG GAA AGC CTA T 3'		QuikChange mutagenesis
GST-FCHO1 μHD (Y733A)	pGEX-4T-1	5' AAC CCC ACT GCC TCC GCC TAC AAC GTG GTG C 3'<br>5' GCA CCA CGT TGT AGG CGG AGG CAG TGG GGT T 3'		QuikChange mutagenesis
GST-FCHO1 μHD (R872A)	pGEX-4T-1	5' GTG GGC AGC GGT TAC GCC ATG TCG CTG GTG 3'<br>5' CAC CAG CGA CAT GGC GTA ACC GCT GCC CAC 3'		QuikChange mutagenesis
GST-FCHO1 μHD (S874G)	pGEX-4T-1	5' AGC GGT TAC CGC ATG GGG CTG GTG AAG AGG AG 3'<br>5' CTC CTC TTC ACC AGC CCC ATG CGG TAA CCG CT 3'		QuikChange mutagenesis
GST-FCHO1 μHD (R872A+S874G)	pGEX-4T-1	5' GTG GGC AGC GGT TAC GCC ATG GGG CTG GTG AAG AGG AG 3'<br>5' CTC CTC TTC ACC AGC CCC ATG GCG TAA CCG CTG CCC AC 3'		QuikChange mutagenesis
GST-FCHO1 μHD (R872M)	pGEX-4T-1	5' CTG GTG GGC AGC GGT TAC ATG ATG TCG CTG GTG AAG AG 3'<br>5' CTC TTC ACC AGC GAC ATC ATG TAA CCG CTG CCC ACC AG 3'		QuikChange mutagenesis
GST-FCHO1 μHD (S732D)	pGEX-4T-1	5' CAG AAC CCC ACT GCC GAC TAC TAC AAC GTG GTG CTG 3'<br>5' CAG CAC CAC GTT GTA GTA GTC GGC AGT GGG GTT CTG 3'		QuikChange mutagenesis
GST-FCHO1 μHD (N735A + R740A)	pGEX-4T-1	5' GCC TCC TAC TAC GCC GTG GTG CTG CTG GCA TAC CAG TTC TCC CGC 3'<br>5' GCG GGA GAA CTG GTA TGC CAG CAG CAC CAC GGC GTA GTA GGA GGC 3'		QuikChange mutagenesis
GST-FCHO1 μHD (T633Y)	pGEX-4T-1	5' GCC ACA GCC TTC TAT GAG TAT GTC CAC GCC TAC TTC CG 3'<br>5' CGG AAG TAG GCG TGG ACA TAC TCA TAG AAG GCT GTG GC 3'		QuikChange mutagenesis
GST-FCHO1 μHD (Y635S)	pGEX-4T-1	5' GCC TTC ACG GAG TCT GTC CAC GCC TAC TTC CG 3'<br>5' CGG AAG TAG GCG TGG ACA GAC TCC GTG AAG GC 3'		QuikChange mutagenesis
GST-FCHO1 μHD (P627D)	pGEX-4T-1	5′ GGC TCC CAG GAT GCC CTG GAC ATA GCC ACA GCC TTC 3′<br>5′ GAA GGC TGT GGC TAT GTC CAG GGC ATC CTG GGA GCC 3′		QuikChange mutagenesis
GST-FCHO1 μHD (A629E)	pGEX-4T-1	5′ CAG GAT GCC CTG CCC ATA GAG ACA GCC TTC ACG GAG 3′<br>5′ CTC CGT GAA GGC TGT CTC TAT GGG CAG GGC ATC CTG 3′		QuikChange mutagenesis
GST-FCHO1 μHD (P627D + A629E)	pGEX-4T-1	5′ GCC TCC CAG GAT GCC CTG GAC ATA GAG ACA GCC TCC ACG GAG TAT G 3′<br>5′ CAT ACT CCG TGA AGG CTG TCT CTA TGT CCA GGG CAT CCT GGG AGC C 3′		QuikChange mutagenesis
GST-FCHO1 μHD (R872E)	pGEX-4T-1	5′ CTG GTG GGC AGC GGT TAC GAG ATG TCG CTG GTG AAG AGG 3′<br>5′ CCT CTT CAC CAG CGA CAT CTC GTA ACC GCT GCC CAC CAG 3′		QuikChange mutagenesis
GFP-FCHO1 (1-889)	pEGFP-C1	5' ATT GAA TTC TAT GTC GTA TTT TGG CGA GC 3'<br>5' GCG TCG ACT CAG CAG CTC ACC AGG TAC 3'	EcoRI/SalI	Directional cloning
GFP-FCHO1 (1-609)	pEGFP-C1	5' CAG ACA GGA CAC TGA GTC TCC CGG G 3'<br>5' CCC GGG AGA CTC AGT GTC CTG TCT G 3'		QuikChange mutagenesis
tdRFP-FCHO1 (1-889)	ptdTomato-C1	5' ATT GAA TTC TAT GTC GTA TTT TGG CGA GC 3'<br>5' GCG TCG ACT CAG CAG CTC ACC AGG TAC 3'	EcoRI/SalI	Directional cloning
tdRFP-FCHO1 (609-889/μHD)	ptdTomato-C1	5' ATA GAA TTC ACA CGG AGT CTC CCG GG 3'<br>5' GCG TCG ACT CAG CAG CTC ACC AGG TAC 3'	EcoRI/SalI	Directional cloning
tdRFP-EPS15(1-896)	ptdTomato-	5' TTC TCG AGG AAT GGC TGC GGC AGC CC 3'	XhoI/BamHI	Directional

tdRFP-EPS15 (1-896/ Δ618-636)	ptdTomato-C1	5'-/5Phos/CAA GTT TGT ATC TGC AAC TGC ATC TG 3'<br>5'-/5Phos/GGA AAA ATT GAT CCA TTT GGT G 3'		Phusion PCR
tdRFP-EPS15 (1-636)	ptdTomato-C1	5' CCT TTC AAG GAT GAT CCT TTT TGA AAA ATT GAT CCA TTT GGT GG 3'<br>5' CCA CCA AAT GGA TCA ATT TTT CAA AAA GGA TCA TCC TTG AAA GG 3'		QuikChange mutagenesis
tdRFP-EPS15 (1-614)	ptdTomato-C1	5' GAC AGA TGC AGT TGC AGA TTA AAA CTT GGA TTT TTT CCA GTC TGA 3'<br>5' TCA GAC TGG AAA AAA TCC AAG TTT TAA TCT GCA ACT GCA TCT GTC 3'		QuikChange mutagenesis
Tac-FCHO1 μHD	pcDNA3.1	5' ATG ATA TCC ACG GAG TCT CCC GGG G 3'<br>5' TAG CGG CCG CTC AGC AGC TCA CCA GGT ACA TCC 3'	EcoRV/NotI	Directional cloning
Tac-FCHO1 μHD (K877E+R879A)	pcDNA3.1	5' CGC ATG TCG CTG GTG GAG AGG GCG TTT GCC ACA GGG ATG TAC CTG 3'<br>5' CAG GTA CAT CCC TGT GGC AAA CGC CCT CTC CAC CAG CGA CAT GCG 3'		QuikChange mutagenesis
Tac-FCHO1 linker	pcDNA3.1	5' CAG ACA GGA CAC TGA GTC TCC CGG G 3'<br>5' CCC GGG AGA CTC AGT GTC CTG TCT G 3'		QuikChange mutagenesis
GFP-FYVE-FCHO1 μHD	pEGFP-C1	5' ATA GAA TTC CAC GGA GTC TCC CGG G 3'<br>5' TAG CGG CCG CTC AGC AGC TCA CCA GGT ACA TCC 3'	EcoRI/NotI	Directional cloning
Tac-Sgip1 μHD	pcDNA3.1	5' TCG ATA TCG CTG AAA GCA CTT CTT C 3'<br>5' AAG CGG CCG CTT AGT TAT CTG CAA AGT AC 3'	EcoRV/NotI	Directional cloning

For siRNA-mediated silencing of the EPS15- and EPS15R-encoding transcripts, siGENOME human EPS15 (2060; 5'-CCACCAAGAUUUCAUGAUA-3', 5'-GAUCGGAACUCCAACAAGA-3', 5'-AAACGGAGCUACAGAUUAU-3', and 5'-CCACACAGCAUUCUUGUAA-3') and human EPS15L1 (58513; 5'-GAAGUUACCUUGAGCAAUC-3', 5'-GGACUUGGCCGAUCCAGAA-3', 5'-GCACUUGGAUCGAGAUGAG-3', and 5'-CAAAGACCAAUUCGCGUUA-3') SMARTpools were purchased from (Dharmacon/GE Heatlthcare) and were used as described (Teckchandani et al., 2012).

Cell culture and transfections

HeLa SS6 (Elbashir et al., 2001), genome edited FCHO1+FCHO2 functionally-null HeLa SS6 clone #64/1.E (1.E cells) (Umasankar et al., 2014), HeLa SS6 cells stably transfected (Keyel et al., 2008) with a plasmid encoding a YFP-tagged β2 subunit of AP-2 (Sorkina et al., 2002), and a HeLa β2-YFP FCHO2-null gene-edited line designated clone #46β were cultured at 37°C in a 5% CO_2 humidified atmosphere in DMEM supplemented with 10% FCS and 2 mM L-glutamine. The β2-YFP-expressing stable cell medium also contained 0.5 mg/ml G418. The HeLa SS6 clone #46β cells were generated using the parental β2-YFP HeLa SS6 cells and the same TALEN pair (Figure S7A) we used previously to create the HeLa SS6 clone #64 FCHO2-null line. The overall method was identical to that used previously (Umasankar et al., 2014) and the genotype and phenotype of the clone #46β cells is reported in Figure S7B-E.

Preparative batches of HeLa cell lysate were made from HeLa SS6 cells grown to confluence in at least six 150-mm Petri dishes. Cells were detached using Cellstripper (Cellgro/Corning) and washed by centrifugation

in PBS prior to solubilization. To seed cells for transfection, trypsinized cells were plated into 35-mm Petri dishes containing 12-mm #1 round glass cover slips, while sterile 35-mm glass-bottomed, poly-D-lysine-coated MatTek dishes were used for time-resolved live-cell imaging studies. Transfections generally used Lipofectamine 2000 (Thermo Fisher) and endotoxin-free DNA prepared with PureYield midiprep kits (Promega).

For siRNA silencing experiments, adherent HeLa clone 1.E cells were transfected twice with sets of four RNA oligonucleotides targeting either EPS15 or EPS15R transcripts together (200 pmol of each pool) in conjunction with Oligofectamine (ThermoFisher Scientific). After the first-round transfections, cells were trypsinized and replated onto round glass cover slips in 35-mm Petri dishes and analyzed ~40 h after the second-round transfections (Umasankar et al., 2012). Duplicate 35-mm dishes were seeded for preparation of lysates and immunoblotting. Under these conditions, this silencing procedure produces an approximately 50% reduction in steady-state EPS15 levels and approximately 75-80% reduction in EPS15R levels in the whole population.

Protein purification

For pull-down assays, control GST and the required GST-fusion proteins were purified from *E. coli* BL21 codon plus RIL cell pellets by one of two procedures. For the majority of proteins, the frozen pellets were resuspended in a buffer of 50 mM Tris-HCl, pH 7.2, 300 mM sodium chloride, 10 mM β-mercaptoethanol and 0.2% (v/v) Triton X-100 on ice, 1 mM PMSF added, and then subject to three cycles of microprobe sonication on ice with a 1-2 min cooling period between each burst of sonication. Alternatively, bacterial pellets were resuspended at room temperature in a 3:1 mixture of B-PER and Y-PER with 200 μg/ml lysozyme, 0.05 U/ml benzonaze and 1 mM PMSF added. In both cases, the extracts were centrifuged at 24,000 *g* for 20 min and the resulting clarified lysate supernatant transferred to a new tube and gluthathione-Sepharose beads added. After incubation at 4°C for ~2 hours with continuous up-and-down mixing, the beads were recovered by centrifugation at 500 *g* for 5 min and the unbound material discarded. Following extensive washing of the beads with PBS and centrifugation, the GST proteins were eluted with a buffer of 50 mM reduced glutathione and 10 mM DTT. Protein concentrations were determined with the Bradford assay using BSA as a standard. Thrombin-cleaved FCHO1 μHD was prepared from the GST-μHD fusion by overnight incubation with thrombin at room temperature. For the monomeric EPS15 (595-896), a C-terminally His_{12}-tagged GST-fusion form was first collected on glutathione-Sepharose, washed and treated with thrombin overnight. The thrombin-released EPS15 was then purified over a Ni-NTA-agarose column and eluted with 50 mM Tris-HCl, pH 8.0, 300 mM sodium chloride and 500 mM imidazole.

Proteins used in structure determination and ITC were similarly expressed as GST fusion proteins in the *E. coli* BL21(DE3) plysS strain at 22°C for 16 hours. Cells were lysed and insoluble material removed by centrifugation. GST fusion proteins were bound to glutathione-Sepharose in buffer A (20 mM Tris-HCl, pH 7.4, 500 mM sodium chloride) with 0.1 mM ABESF, washed extensively with 100 volumes of buffer A and the

proteins cleaved from GST by overnight incubation at 22°C with thrombin. Proteins were concentrated to ~15 mg/ml and further purified over a HiLoad 26/600 Superdex S200 gel filtration column (GE Healthcare) equilibrated in buffer A with 0.1 mM ABESF and 2 mM DTT. Any remaining GST or uncleaved fusion proteins were removed by passage through a 1 ml glutathione-Sepharose column. For crystallization, protein was gel filtered into in buffer A with ABESF and for ITC into 100 mM Tris-HCl pH 7.5, 200 mM sodium chloride and 0.25 mM TCEP. After first attempting crystallization of μHDs from human, *Xenopus* or zebrafish (*D. rerio*) Fcho1/2, both alone and together with synthetic EPS15 peptides, we switched to in-frame chimeras of human EPS15 (615-637) with either the human FCHO1 or *D. rerio* Fcho1 μHD, only the latter of which crystallized. The best crystals of the EPS15-μHD chimeric protein (15 mg/ml) grew over a period of 2-3 weeks by hanging drop vapor diffusion against a reservoir containing 100 mM Bis-Tris propane pH, 6.0, 200 mM sodium citrate, 22% (v/v) PEG 3350 and 10 mM DTT. For native and xenon derivatives, crystals were cryoprotected in mother liquor fortified with 22% (v/v) glycerol and flash frozen. Xe derivatives were made by pressurizing crystals to between 10 and 12 atmospheres of pressure in a homemade Xe chamber. Crystals were of the space group H3 with two molecules in the asymmetric unit (a=b=239.78 c=44.09, $\alpha=\beta=90.00$ $\gamma=120.00$) and diffracted at best to 2.4 Å at Diamond Light Source synchrotron. Data were collected using an inverse beam strategy with 5° wedges. The structure was solved by single-wavelength anomalous dispersion (SAD) using AutoSol (Terwilliger et al., 2009). Initial building was accomplished with ten cycles of Buccaneer (Cowtan, 2006) and refined using alternating cycles of Phenix.refine (Adams et al., 2010; Afonine et al., 2012) and manual rebuilding in COOT (Emsley et al., 2010). The final model contains 594 residues, 40 water molecules and one Zn^{2+} atom, and was refined to final R/Rfree of 0.199/0.234 (Table S1). The concave face of the Fcho1 μHD is overall considerably more curved than in all other μHDs that were used as models to attempt to solve the structure by molecular replacement, explaining the lack of success using this technique. In addition, the two molecules in the asymmetric unit interact with a metal ion (Zn^{2+} on the basis of weak anomalous scattering) coordinated by H1031 and C1033 in each μHD. These residues are not conserved in Fchos of other species, so we do not consider dimerization biologically relevant.

Rat brain cytosol was prepared in a homogenization buffer of 25 mM Hepes-KOH, pH 7.2, 250 mM sucrose, 2 mM EDTA and 2 mM EGTA. Rapidly thawed frozen brain tissue was homogenized in a Waring Blendor at 4°C in the presence of homogenization buffer with 2 mM PMSF, 5 mM benzamidine and cOmplete protease inhibitor tablets. The resulting thick homogenate was centrifuged at 15,000 *g* for 20 min and the postnuclear supernatant fraction recentrifuged at 17,500 *g* for 20 min. The supernatant was then centrifuged at 105,000 *g* for 60 min. The resulting high-speed supernatant (cytosol) was stored in small frozen aliquots at -80°C. Before use in pull-down assays, the brain cytosol was adjusted to 1 × assay buffer using a 10 × stock solution on ice. HeLa cell Triton X-100 lysates were prepared by first collecting confluent cells from Petri dishes using Cellstripper and centrifuging at 500 *g* for 5 min. The cell pellet was resuspended in cold 25 mM Hepes-KOH, pH 7.2, 125 mM potassium acetate, 5 mM magnesium acetate, 2 mM EDTA, 2 mM EGTA, 2 mM DTT and 1% Triton X-100 and incubated on ice for 30 min with occasional mixing. After centrifugation at 24,000 *g* for 20 min to remove insoluble material, the supernatant (lysate) was stored in frozen aliquots at -

80°C. Thawed cytosol/lysate samples for assays were centrifuged at 125,000 *g* for 20 min at 4°C immediately before use.

GST pull-down assays

The standard format for biochemical assays entails first immobilizing a measured amount of GST or GST-fusion protein onto a standard volume of glutathione-Sepharose beads. In microfuge tubes, the appropriate protein mass was diluted into PBS on ice up to a final volume of 750 μl and then 60 μl of a cold 50% slurry of glutathione-Sepharose beads in PBS added to each tube. After incubation at 4°C with continuous up-and-down mixing, the Sepharose beads were recovered by centrifugation (10,000 *g*, 1 min) and the supernatants aspirated and discarded. Each bead pellet was washed twice with cold assay buffer composed of 25 mM Hepes-KOH, pH 7.2, 125 mM potassium acetate, 5 mM magnesium acetate, 2 mM EDTA, 2 mM EGTA and 2 mM DTT. After the second spin, the majority of the buffer was carefully aspirated, leaving a final equivalent volume of ~50 μl in each tube. Aliquots of rat brain cytosol or HeLa cell Triton X-100 lysate were thawed from -80°C and centrifuged at 125,000 *g* for 20 minutes at 4°C to remove insoluble particulate matter before addition to the tubes of immobilized GST or GST-fusion protein. Typically 200-250 μl of cytosol/lysate was added, the beads resuspended, and then incubated at 4°C for 60 min with continual up-and-down mixing. Binding assays were terminated by centrifugation (10,000 *g*, 1 min, 4°C) and an aliquot (typically 60 μl) removed and transferred to a new microfuge tube. At this stage, the incubations are still at steady state, so comparison of the differences in binding partner protein recovery in the various supernatant fractions presented in the figures *versus* the control GST can be used to evaluate the extent of binding to the relevant GST-fusion protein being tested. The pellets were then washed by centrifugation four times with ~1.5 ml/wash ice-cold PBS and the supernatants aspirated and discarded. The Sepharose beads were resuspended in reducing SDS sample buffer, and the volumes adjusted manually so that all tubes were equivalent. Importantly, during washing, the reactions are no longer at steady state due to repeated dilution, and so comparison of the pellets may give the impression that a given protein bound differentially to various immobilized GST-fused mutants. This is why we present both supernatant and pellet fractions; it allows assessment of whether a particular mutation, or set of mutations, alters either the on-rate (k_{on}), the off-rate (k_{off}), or both. For example in comparing the GST-*Hs* FCHO1 μHD mutants, the N735A+R740A protein is less compromised than a K877E+R879A μHD because less EPS15 and intersectin 1 and no EPS15R remain in the supernatant fractions, and a bound pool of these proteins is evident after washing. After boiling the pull-down samples and centrifugation (12,000 *g*, 1 min), aliquots were fractionated by SDS-PAGE. In all of our pull-down experiments, because of the differences in volumes, we routinely load onto each gel 5 × more of each pellet fraction than of the corresponding supernatant. This accounts for the intensity of the pellet signal often being substantially higher than the control (GST) supernatant fraction.

Isothermal titration microcalorimetry (ITC)

Purified μHDs were gel filtered into 100 mM Tris-HCl pH 7.5, 200 mM sodium chloride and 0.25 mM TCEP. Synthetic peptides (EPS15 residues 615-637 and truncations of thereof) (Genscript) were dissolved in the

same buffer. Experiments were performed using a Nano ITC from TA Instruments. The *Hs* FCHO1 μHD at 0.11 mM was placed in the cell at 12°C and peptides at concentrations between 1.6 and 1.7 μM depending on peptide were titrated in with 19 injections of 2 μl with injections separated by 5 minutes. Experiments with the zebrafish Fcho1 μHD were carried out with 0.3 μM protein in the cell and were carried out at 4°C due to the lower thermal stability of this protein. In addition, the *Hs* FCHO1 μHD mutants in the area of the α-helical portion of subdomain A were labile and prone to aggregation when assayed at 12°C. Therefore, the P627D, A629E and P627D+A629E mutants were similarly tested with the reaction cell equilibrated to 4°C. An appropriate buffer into protein blank was subtracted from all data runs and for constructs which displayed measurable binding, a minimum of four independent runs that showed clear saturation of binding were used to calculate the mean K_D of the reaction, its stoichiometry (n) and their corresponding SEM values. Analysis of results and final figure preparation were carried out using the NanoAnalyze™ software.

SDS-PAGE and immunoblotting

Discontinuous polyacrylamide gels were cast manually using an acrylamide-bis-acrylamide stock solution that differs from the typical 30:0.8 ratio by having half the bis-acrylamide (30:0.4). The utility of this altered ratio of crosslinker is that it markedly improves the separation of closely-spaced bands. The standard denaturing Laemmli buffer system was used with reducing sample buffer. After electrophoresis using constant voltage, gels were either stained in Coomassie Blue in 40% methanol, 10% acetic acid or transferred to nitrocellulose. For blotting, the gels were first equilibrated for ~5-10 min in ice-cold transfer buffer of 15.6 mM Tris, 120 mM glycine to reduce the amount of SDS, and then the gel assembled upon a sheet of wet nitrocellulose on moistened filter paper and transferred in ice-cold transfer buffer at a constant 110 V for 75 min. Proteins were visualized using 0.2% Ponceau S in acetic acid, the position of the molecular mass standards marked in pencil, cut into appropriate numbered sections horizontally with a scalpel blade, and then the nitrocellulose destained completely in transfer buffer at room temperature. Non-specific binding sites on the blots were quenched at room temperature with 5% non-fat dried milk in a blotting buffer of 10 mM Tris-HCl, pH 7.8, 150 mM sodium chloride and 0.1% Tween 20. The relevant portions of the blots were then incubated for 1-2 hours at room temperature with primary antibodies diluted in 1% milk in the blotting buffer, washed in blotting buffer and then incubated in 1:5,000 dilutions of anti-mouse/rabbit/goat IgG coupled to horseradish peroxidase for 1 hour at room temperature. Following extensive washing in blotting buffer, the location of bound antibodies was detected with an enhanced chemiluminescence-type reagent (HyGlo, Denville Scientific) and standard X-ray film.

Immunofluorescence staining

We used two different protocols for labeling cells attached to glass coverslips with appropriate antibodies. The first is a standard procedure using paraformadehyde fixation, Triton X-100 permeabilization and quenching in a buffer with 5% normal goat serum in PBS (Keyel et al., 2006; Umasankar et al., 2012). The modified method generally provides superior results with many, but not all, antibodies. In this procedure, cells were first washed twice with PBS and then fixed for 30 min at room temperature in 4% freshly-prepared

paraformaldehyde in PBS adjusted to pH 8.0. The fixed cells were then washed 2 × 5 min with PBS and subsequently incubated in a quenching solution of 75 mM ammonium chloride and 20 mM glycine dissolved in the pH 8.0 PBS. Next, the cells were permeabilized with 0.1% Triton X-100 in PBS, pH 8.0 at room temperature for 30 min. After washing the cells 3 x 5 min with PBS, the cells were blocked for 10 min at 37°C with a mixture of 5% (v/v) normal goat serum in 0.66% fish skin gelatin, 0.05% saponin in pH 8.0 PBS (buffer IF). Primary antibodies were added after dilution in buffer IF and centrifugation at >12,000 *g* for 5 min to remove aggregated material. Incubation was at 37°C for 60 min, followed by three quick rinses with buffer IF and then 3 × 5 min washes using the same buffer. Secondary antibodies were prepared and used in the same manner as the primary antibodies. The cells were rinsed quickly 3 × with buffer IF followed by 3 × 5 min washes in buffer IF, and then 3 x 5 min washes with PBS. A solution of 0.1% Triton X-100 in PBS was added next, for 5 min, then a 5 min wash in PBS and then a post-fixation step with 4% paraformaldehyde in 100 mM sodium cacodylate, pH 7.4 at room temperature for 30 min. The samples were again incubated in quenching solution for 10 min, washed 2 × 5 min with PBS, pH 8.0, and finally mounted on glass slides in Cytoseal.

Microscopy

Confocal imaging of fixed and fluorescently stained samples was performed on an inverted Olympus FV1000 microscope equipped with a PlanApo N (60 × /1.42 numerical aperture) oil objective. Appropriate excitation and emission wavelengths were preprogrammed by the instrument running the FV10-ASW software, and emission signals in the different channels were always collected in the sequential scan mode. Images were exported in TIFF file format and imported into Adobe Photoshop CS4 for minor adjustments to brightness and/or contrast. Quantitation of transferrin uptake in confocal image files was performed using the linescan application within Metamorph (Molecular Devices). The extent of transferrin internalization in confocal image stacks that were collected following the pulse-chase format (as in Fig. ure 5) was analyzed by using Fiji software (Schindelin et al., 2012) . Images were corrected for the background. A 10.09 μm × 10.09 μm area was selected as a region of interest (ROI) in basal, middle and upper sections of each cell in the *z* stack. Each optical section was saved as a separate image and processed separately. A mask for each image was created by applying a manual threshold. The mask was then used on the corresponding ROI image to measure the number and intensity of each spot in different cells. For cells in the different populations (control untransfected, Tac-μHD or Tac-linker expressing), the intensity values of spots in each population set were pooled together and plotted in the histogram. For the different populations, the same total area, 2,443.4 μm^2, was used for the comparative data presented in Figure S6.

TIRFM image acquisition

Cultured HeLa clone #46β cells in glass-bottomed MatTek dishes were examined using TIRFM optics at 37°C with a Nikon TI microscope equipped with both confocal (A1R spectral), wide field, and through-the-objective type TIRFM capabilities. The camera used for all TIRFM and widefield imaging experiments on the Nikon TI microscope was an Andor Zyla 5.5 at full frame with no binning, the laser launch is a 4 color (405

nm, 488 nm, 561 nm and 647 nm lines). All images were collected using NIS Elements software. The appropriately treated HeLa cell clones in MatTek dishes were mounted in a temperature and CO_2 controlled humidified chamber (Tokai Hit, Tokyo Japan) in a Mad City Labs Piezo controlled *z* stage. Using a drift correction device (Perfect Focus, Nikon Instruments) cells were brought into focus and the *z* position locked. Individual fields were selected blindly and then sequential images collected using conditions that caused minimal signal loss due to bleaching with the illuminating laser. Laser conditions varied with experiments, either YFP alone (488 nm excitation 525/40 nm emission filter) or with the 561 nm (600/50 emission filter) or 647 nm (700/75 emission filter). To ensure perfect image registration between colors, a single 4 color TIRFM filter cube (Chroma tech, Brattleboro VT) was used with a high speed (20 ms change time) filter wheel (FLI, NY) to block bleed through between channels.

Rapid-freeze deep-etch electron microscopy

Cells were cultured on small oriented pieced of glass coverslip and ruptured with a brief burst of sonication at room temperature. The formaldehyde fixed adherent cell membrane preparations were immunogold labeled with rabbit anti-FCHO2 antibodies followed by colloidal-gold labeled anti-rabbit IgG, as we have described previously (Edeling et al., 2006; Hawryluk et al., 2006). A detailed discussion of the technique, procedures and possible pitfalls of generating immunogold-labeled platinum replicas of cell cortices is available (Heuser, 2000).

Supplemental References

Adams, P.D., Afonine, P.V., Bunkoczi, G., Chen, V.B., Davis, I.W., Echols, N., Headd, J.J., Hung, L.W., Kapral, G.J., Grosse-Kunstleve, R.W.*, et al.* (2010). PHENIX: a comprehensive Python-based system for macromolecular structure solution. Acta Crystallogr D Biol Crystallogr *66*, 213-221.

Afonine, P.V., Grosse-Kunstleve, R.W., Echols, N., Headd, J.J., Moriarty, N.W., Mustyakimov, M., Terwilliger, T.C., Urzhumtsev, A., Zwart, P.H., and Adams, P.D. (2012). Towards automated crystallographic structure refinement with phenix.refine. Acta Crystallogr D Biol Crystallogr *68*, 352-367.

Burgos, P.V., Mardones, G.A., Rojas, A.L., daSilva, L.L., Prabhu, Y., Hurley, J.H., and Bonifacino, J.S. (2010). Sorting of the Alzheimer's disease amyloid precursor protein mediated by the AP-4 complex. Dev Cell *18*, 425-436.

Chen, H., and De Camilli, P. (2005). The association of epsin with ubiquitinated cargo along the endocytic pathway is negatively regulated by its interaction with clathrin. Proc Natl Acad Sci U S A *102*, 2766-2771.

Cowtan, K. (2006). The Buccaneer software for automated model building. 1. Tracing protein chains. Acta Crystallogr D Biol Crystallogr *62*, 1002-1011.

Edeling, M.A., Mishra, S.K., Keyel, P.A., Steinhauser, A.L., Collins, B.M., Roth, R., Heuser, J.E., Owen, D.J., and Traub, L.M. (2006). Molecular switches involving the AP-2 β2 appendage regulate endocytic cargo selection and clathrin coat assembly. Dev Cell *10*, 329-342.

Elbashir, S.M., Harborth, J., Lendeckel, W., Yalcin, A., Weber, K., and Tuschl, T. (2001). Duplexes of 21-nucleotide RNAs mediate RNA interference in cultured mammalian cells. Nature *411*, 494-498.

Emsley, P., Lohkamp, B., Scott, W.G., and Cowtan, K. (2010). Features and development of Coot. Acta Crystallogr D Biol Crystallogr *66*, 486-501.

Hawryluk, M.J., Keyel, P.A., Mishra, S.K., Watkins, S.C., Heuser, J.E., and Traub, L.M. (2006). Epsin 1 is a polyubiquitin-selective clathrin-associated sorting protein. Traffic *7*, 262-281.

Hein, M.Y., Hubner, N.C., Poser, I., Cox, J., Nagaraj, N., Toyoda, Y., Gak, I., Weisswange, I., Mansfeld, J., Buchholz, F.*, et al.* (2015). A human interactome in three quantitative dimensions organized by stoichiometries and abundances. Cell *163*, 712–723.

Heuser, J. (2000). The production of 'cell cortices' for light and electron microscopy. Traffic *1*, 545-552.

Keyel, P.A., Mishra, S.K., Roth, R., Heuser, J.E., Watkins, S.C., and Traub, L.M. (2006). A single common portal for clathrin-mediated endocytosis of distinct cargo governed by cargo-selective adaptors. Mol Biol Cell *17*, 4300-4317.

Keyel, P.A., Thieman, J.R., Roth, R., Erkan, E., Everett, E.T., Watkins, S.C., and Traub, L.M. (2008). The AP-2 adaptor β2 appendage scaffolds alternate cargo endocytosis. Mol Biol Cell *19*, 5309-5326.

Owen, D.J., and Evans, P.R. (1998). A structural explanation for the recognition of tyrosine-based endocytotic signals. Science *282*, 1327-1332.

Reider, A., Barker, S.L., Mishra, S.K., Im, Y.J., Maldonado-Baez, L., Hurley, J.H., Traub, L.M., and Wendland, B. (2009). Syp1 is a conserved endocytic adaptor that contains domains involved in cargo selection and membrane tubulation. EMBO J *28*, 3103-3016.

Schindelin, J., Arganda-Carreras, I., Frise, E., Kaynig, V., Longair, M., Pietzsch, T., Preibisch, S., Rueden, C., Saalfeld, S., Schmid, B.*, et al.* (2012). Fiji: an open-source platform for biological-image analysis. Nat Methods *9*, 676-682.

Sorkina, T., Huang, F., Beguinot, L., and Sorkin, A. (2002). Effect of tyrosine kinase inhibitors on clathrin-coated pit recruitment and internalization of epidermal growth factor receptor. J Biol Chem *277*, 27433-27441.

Teckchandani, A., Mulkearns, E.E., Randolph, T.W., Toida, N., and Cooper, J.A. (2012). The clathrin adaptor Dab2 recruits EH domain scaffold proteins to regulate integrin b1 endocytosis. Mol Biol Cell *23*, 2905-2916.

Terwilliger, T.C., Adams, P.D., Read, R.J., McCoy, A.J., Moriarty, N.W., Grosse-Kunstleve, R.W., Afonine, P.V., Zwart, P.H., and Hung, L.W. (2009). Decision-making in structure solution using Bayesian estimates of map quality: the PHENIX AutoSol wizard. Acta Crystallogr D Biol Crystallogr *65*, 582-601.

Traub, L.M., Downs, M.A., Westrich, J.L., and Fremont, D.H. (1999). Crystal structure of the α appendage of AP-2 reveals a recruitment platform for clathrin-coat assembly. Proc Natl Acad Sci USA *96*, 8907-8912.

Umasankar, P.K., Ma, L., Thieman, J.R., Jha, A., Doray, B., Watkins, S.C., and Traub, L.M. (2014). A clathrin coat assembly role for the muniscin protein central linker revealed by TALEN-mediated gene editing. eLife *3*, e04137.

Umasankar, P.K., Sanker, S., Thieman, J.R., Chakraborty, S., Wendland, B., Tsang, M., and Traub, L.M. (2012). Distinct and separable activities of the endocytic clathrin-coat components Fcho1/2 and AP-2 in developmental patterning. Nat Cell Biol *14*, 488-501.
