# Supplementary material for: Transient Fcho1/2⋅Eps15/R⋅AP-2 Nanoclusters Prime the AP-2 Clathrin Adaptor for Cargo Binding
Source: Dev Cell. 2016 Jun 6;37(5):428–43. doi: 10.1016/j.devcel.2016.05.003 (PMC4921775; doi:10.1016/j.devcel.2016.05.003)
Supplement: Document S2. Article plus Supplemental Information [file mmc2.pdf]

Developmental Cell

Article

Transient Fcho1/2·Eps15/R·AP-2 Nanoclusters Prime the AP-2 Clathrin Adaptor for Cargo Binding

Graphical Abstract

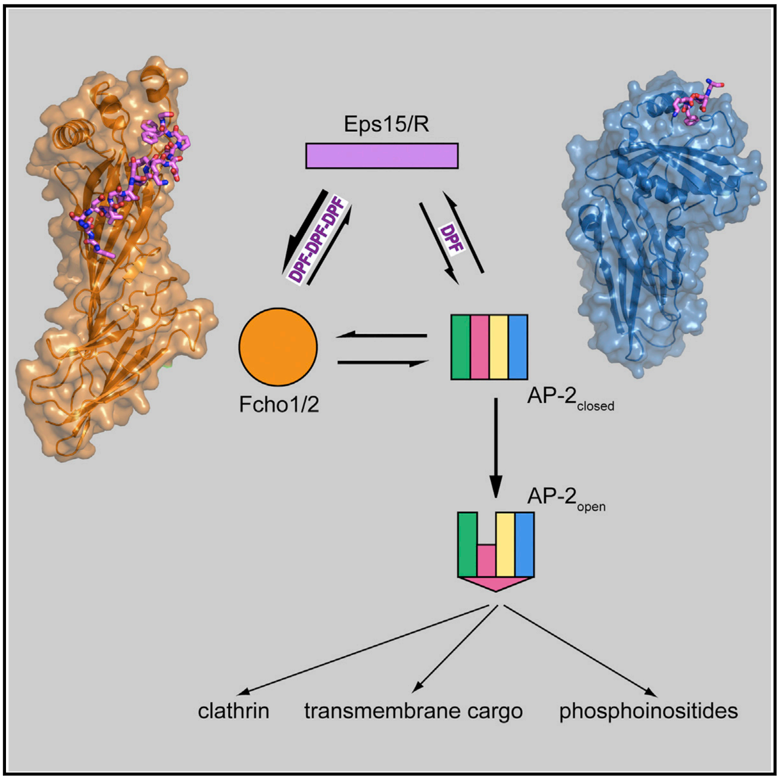

Authors

Li Ma, Perunthottathu K. Umasankar, Antoni G. Wrobel, ..., Simon C. Watkins, David J. Owen, Linton M. Traub

Correspondence

traub@pitt.edu

In Brief

How clathrin-coated vesicles initiate at the plasma membrane is uncertain. Ma et al. show through structural and biochemical analyses that a three-way interaction between early-arriving endocytic factors Eps15/R and Fcho1/2, and the adaptor AP-2, promotes membrane deposition of AP-2 in open conformation for sustained clathrin-coat assembly and cargo loading.

Highlights

- The endocytic pioneer protein Eps15 engages AP-2 and Fcho1/2 noncompetitively
- Structural analysis shows arrayed DPF motif triad in Eps15 for Fcho1/2 μHD binding
- DPF-based codes direct transient Fcho1/2·Eps15/R·AP-2 ternary complex formation
- In ternary complex, Fcho1 interdomain linker primes AP-2 for cargo capture

Accession Numbers

5JP2

Ma et al., 2016, Developmental Cell *37*, 428–443
June 6, 2016 © 2016 Elsevier Inc.
http://dx.doi.org/10.1016/j.devcel.2016.05.003

Developmental Cell
Article

Transient Fcho1/2·Eps15/R·AP-2 Nanoclusters Prime the AP-2 Clathrin Adaptor for Cargo Binding

Li Ma,[1,3] Perunthottathu K. Umasankar,[1,3] Antoni G. Wrobel,[2,3] Anastasia Lymar,[1] Airlie J. McCoy,[2] Sachin S. Holkar,[1] Anupma Jha,[1] Tirthadipa Pradhan-Sundd,[1] Simon C. Watkins,[1] David J. Owen,[2] and Linton M. Traub[1,*]
[1]Department of Cell Biology, University of Pittsburgh School of Medicine, 3500 Terrace Street, S312 BST, Pittsburgh, PA 15261, USA
[2]Cambridge Institute for Medical Research, University of Cambridge, Cambridge CB2 0XY, UK
[3]Co-first author
*Correspondence: traub@pitt.edu
http://dx.doi.org/10.1016/j.devcel.2016.05.003

SUMMARY

Clathrin-coated vesicles form by rapid assembly of discrete coat constituents into a cargo-sorting lattice. How the sequential phases of coat construction are choreographed is unclear, but transient protein-protein interactions mediated by short interaction motifs are pivotal. We show that arrayed Asp-Pro-Phe (DPF) motifs within the early-arriving endocytic pioneers Eps15/R are differentially decoded by other endocytic pioneers Fcho1/2 and AP-2. The structure of an Eps15/R·Fcho1 μ-homology domain complex reveals a spacing-dependent DPF triad, bound in a mechanistically distinct way from the mode of single DPF binding to AP-2. Using cells lacking FCHO1/2 and with Eps15 sequestered from the plasma membrane, we establish that without these two endocytic pioneers, AP-2 assemblies are fleeting and endocytosis stalls. Thus, distinct DPF-based codes within the unstructured Eps15/R C terminus direct the assembly of temporary Fcho1/2·Eps15/R·AP-2 ternary complexes to facilitate conformational activation of AP-2 by the Fcho1/2 interdomain linker to promote AP-2 cargo engagement.

INTRODUCTION

The minute-to-minute protein composition of the eukaryotic plasma membrane is managed by clathrin-mediated endocytosis (CME) (Bitsikas et al., 2014). Rapid internalization occurs at specialized zones called clathrin-coated structures (CCSs), where signal-dependent cargo packaging is handled by the heterotetrameric AP-2 adaptor complex with an attendant set of clathrin-associated sorting proteins (CLASPs) (Reider and Wendland, 2011; Traub, 2009). As new CCSs initiate, a subset of coat machinery congregates first, priming the plasma membrane for effective transport vesicle production. These early-arriving—pioneer—proteins include AP-2 and clathrin, the principal structural units of surface-derived CCSs, as well as Eps15/R (epidermal growth factor pathway substrate 15/Eps15 related) and Fcho1/2 (Fer/CIP4 homology domain only protein 1 or 2) (Taylor et al., 2011). The pioneer module of endocytic proteins is densely interconnected; deposition and residence of these factors depends on physical connections with the organizing plasma membrane lipid phosphatidylinositol 4,5-bisphosphate (PtdIns(4,5)P_2) (Antonescu et al., 2011; Schifferer et al., 2015; Zoncu et al., 2007) and on direct connections among one another, typically mediated by short linear peptide interaction motifs (McMahon and Boucrot, 2011; Traub, 2009).

The lack of a tightly coupled energy input suggests that arrival of AP-2, pioneers, and CLASPs at the plasma membrane is a stochastic phenomenon (Ehrlich et al., 2004; Godlee and Kaksonen, 2013; Larson et al., 2014), in part underpinning the variability in the precise location of CCS initiation. Given the multiplicity of possible protein-protein interactions at nucleation, it is uncertain how timely forward progression is achieved, and how promiscuous or off-pathway associations are avoided. This issue is critical, as the autoinhibited soluble AP-2 adaptor requires allosteric activation at CCSs (Jackson et al., 2010; Kelly et al., 2014).

Fcho1 and Fcho2 likely play a key role in local AP-2 restructuring, as they are among the earliest proteins to mark a nascent CCS (Henne et al., 2010; Taylor et al., 2011) and affect AP-2 conformation directly (Hollopeter et al., 2014; Umasankar et al., 2014). The two paralogs contain an N-terminal crescent-shaped, membrane-binding EFC (extended FCH) domain also called an F-BAR domain (Henne et al., 2007). The leading EFC domain is followed by an evolutionarily least conserved and intrinsically disordered segment of ~200 residues that associates with the AP-2 adaptor physically (Hollopeter et al., 2014; Umasankar et al., 2012, 2014). But what makes Fchos unusual is that the C-terminal SH3 domain typical of most EFC-domain proteins is replaced by a μ-homology domain (μHD), distantly related in primary sequence to cargo-selective μ subunits of the heterotetrameric clathrin adaptors such as AP-2 (Reider et al., 2009). This combination of an EFC domain with a μHD is exclusive and phylogenetically conserved in opisthokonts. The thus misnamed Fcho1 and Fcho2 share overall domain architecture (Katoh, 2004), while the neuron-enriched Sgip1 (SH3-domain GRB2-like [endophilin] interacting protein 1) protein has a μHD but lacks the folded EFC domain (Uezu et al., 2007). The μHD of all three (designated the muniscins) binds directly to the pioneer protein Eps15 (Henne et al., 2010; Reider et al., 2009; Uezu et al., 2007; Umasankar et al., 2012). To better comprehend the functional consequence of inaugural protein encounters at a clathrin assembly zone, we report three sequential stages of inquiry. First, we delineate the minimal sequence

 © 2016 Elsevier Inc.

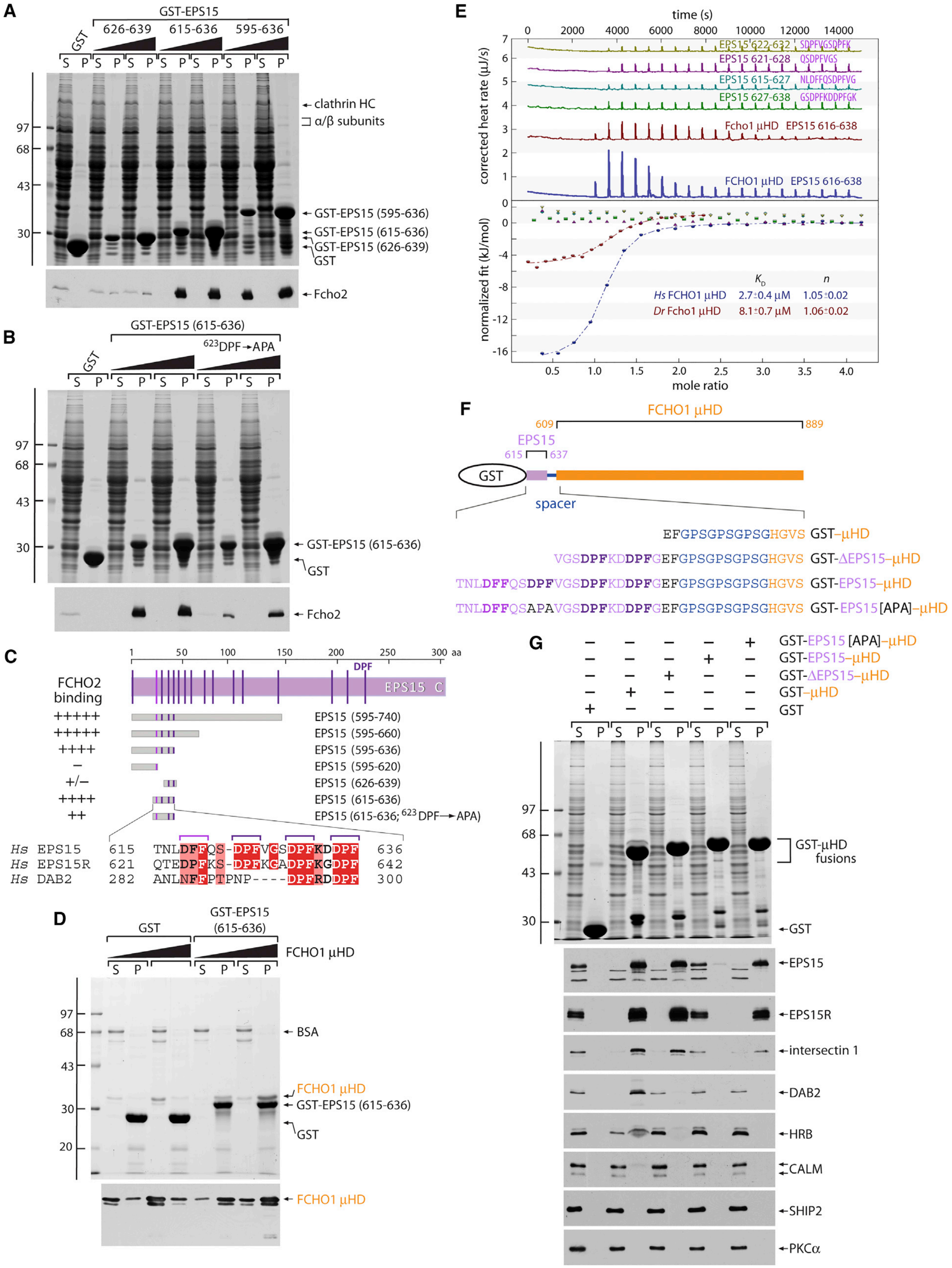

(legend on next page)

tract in Eps15/R necessary to bind the μHD. Next, a 2.4-Å-resolution structure of these two interaction partners provides an atomic-level description of the binding mechanism. Lastly, delineating the structurally distinct manners in which the μHD and AP-2 appendages bind to Asp-Pro-Phe (DPF) triplets within Eps15/R allows us to formulate and test in vivo a concept for pioneer-coordinated activation of AP-2 at plasma membrane bud sites by Eps15 bringing conformationally closed AP-2 into proximity with the Fcho interdomain linker.

RESULTS

A μHD Interaction Network

Fcho1 and Fcho2 have numerous binding partners (Henne et al., 2010; Mulkearns and Cooper, 2012; Reider et al., 2009; Uezu et al., 2007; Umasankar et al., 2012). The chief interaction surface is the globular C-terminal μHD. This is seen as apparent loss of coincidence of an expressed GFP-FCHO1 lacking the μHD with endogenous AP-2- and HRB-positive surface puncta in transfected HeLa cells (Figures S1A and S1B). Conversely, a tandem dimer red fluorescent protein (tdRFP)-tagged FCHO1 μHD alone (residues 609–889) localizes to AP-2-marked CCSs when transiently expressed (Figures S1C and S1D). The FCHO1 μHD binds directly to HRB as well as to EPS15, EPS15R, intersectin 1, DAB2, and CALM (Figure S1E) (Henne et al., 2010; Reider et al., 2009; Umasankar et al., 2012). Amino acid divergence between the FCHO1 and structurally related FCHO2 (53% identity) and Sgip1 (49% identity) μHDs is paralleled by differences in partner protein selectivity; FCHO2 and Sgip1 contact a subset of FCHO1 partners (Mulkearns and Cooper, 2012; Uezu et al., 2011; Umasankar et al., 2012) including EPS15 (Figure S1E), but only the FCHO1 μHD binds to HRB and CALM in our assays.

The association of muniscin μHDs with EPS15 requires the unstructured C terminus (residues 595–896) of the protein (Cupers et al., 1997), rich in tripeptide DPF repeats. The AP-2 α appendage binds to this general segment of EPS15 as well, but the mode of engagement is clearly different; AP-2 binding increases linearly with additional EPS15 DPFs (Benmerah et al., 1996; Iannolo et al., 1997) but Fcho2 binding does not (Figure S1F). When EPS15 (595–896) is immobilized on excess glutathione S-transferase (GST)-AP-2 α appendage, the FCHO1 μHD can simultaneously also bind EPS15 (Figures S1G and S1H). This suggests non-overlapping DPF targets, as ternary complexes can form by synchronous binding to different regions of the EPS15 C terminus. Further truncation/deletion analysis defines a minimum region (^{615}TNLDFFQSDPFVGSDPFKDDPF) of EPS15 sufficient for engaging Fcho1/2 (Figures 1A–1C). FCHO1 μHD binding to EPS15 (615–636) is dose dependent and direct (Figure 1D). This EPS15 tract contains three closely spaced DPF repeats (and a leading DFF triplet) highly conserved in chordates, and is additionally present in EPS15R (Figure 1C). The distal region (^{629}DPFKDDPF) of the EPS15 μHD-binding element is nearly identical to a small portion of the CLASP DAB2 (Figures S1 and 1C) that is needed for a direct association with Fcho2 (Henne et al., 2010; Mulkearns and Cooper, 2012; Umasankar et al., 2012). Yet EPS15 and EPS15R bind the μHD with higher apparent affinity than DAB2 (see Figure 3F), suggesting the participation of the first of the three tandem DPFs, which is not found in DAB2 (Figure 1C) (Mulkearns and Cooper, 2012).

Dissecting the DPF-Based EPS15 μHD-Binding Sequence

The role of the proximal half of the mapped DPF tract on μHD engagement is highlighted by the substantially reduced apparent affinity of cytosolic Fcho2 for the GST-EPS15 fusion protein when either residues 615–625 (TNLDFFQSDPF) are removed (Figure 1A) or a ^{623}DPF→APA mutation is introduced into the longer 615–636 form (Figure 1B). No binding of four short peptides encompassing only one or two DPFs between EPS15 residues 616 and 632 is detectable by isothermal titration calorimetry (ITC) (Figure 1E). In agreement, artificially fusing portions of EPS15 directly to the N terminus of the FCHO1 μHD (Figure 1F) affects free partner binding, presumably through intramolecular competition. Fusing EPS15 residues 626–637 (ΔEPS15; containing only DPFX_2DPF), while not interfering effectively with EPS15/R or intersectin 1 binding, prohibits the engagement of the weaker-binding proteins, including DAB2 (Figure 1G). By contrast, linking EPS15 residues 615–637 (with DFF and DPFX_3DPFX_2DPF) essentially eliminates all partner binding. A ^{623}DPF→APA substitution in the full (residues 615–637) tandem fusion diminishes EPS15/R and intersectin binding more efficiently than does the short fusion (residues 626–637). This again implicates the proximal section of the EPS15 sequence in higher-affinity binding. Neither HRB nor CALM have related DPFX_2DPF motifs within the unstructured polypeptide regions, but are Phe rich, suggesting that the weaker affinity of these FCHO1 μHD partners likely reflects use of variant but related interaction motif(s).

An ectopic yet experimentally tractable manifestation of μHD partner engagement occurs in cultured cells in the context of a customized membrane-anchored Tac-FCHO1 μHD fusion

Figure 1. A Minimal EPS15 Interaction Motif

(A and B) GST pull-down assays with rat brain cytosol and 250 μg of GST and either 50 or 250 μg of the indicated GST-EPS15 fusion proteins. SDS-PAGE-separated supernatant (S, 2%) and pellet (P, 10%) fractions were stained or immunoblotted with antibodies against Fcho2.

(C) Location of DPF triplets in the EPS15 C-terminal domain, with the relative location of the various fragments tested below. Alignment of *Homo sapiens* (*Hs*) EPS15, EPS15R, and DAB2 sequences corresponding to the minimal motif, boxed with red for identity and pink for similarity.

(D) GST pull-down assay with purified FCHO1 μHD and ~50 μg of either GST or GST-μHD fusion protein in the presence of carrier BSA. SDS-PAGE-separated supernatant and pellet fractions were stained or immunoblotted with an anti-FCHO1 monoclonal antibody.

(E) Representative ITC experiments with the *Danio rerio* (*Dr*) Fcho1 μHD and EPS15 616–638 peptide or the *Hs* FCHO1 μHD titrated with the 616–638 or indicated EPS15 peptides. K_D, reaction stoichiometry (*n*), and errors were calculated from a minimum of four runs.

(F) Cartoon representation of GST-EPS15-FCHO1 μHD chimeras, with the specific EPS15 sequence for each variant preceding the GlyProSer spacer shown below.

(G) GST pull-down assay using HeLa cell lysate and 250 μg of either GST or GST-μHD fusion proteins as indicated. Separated supernatant and pellet fractions were stained or replicates immunoblotted with the indicated antibodies as in (A).

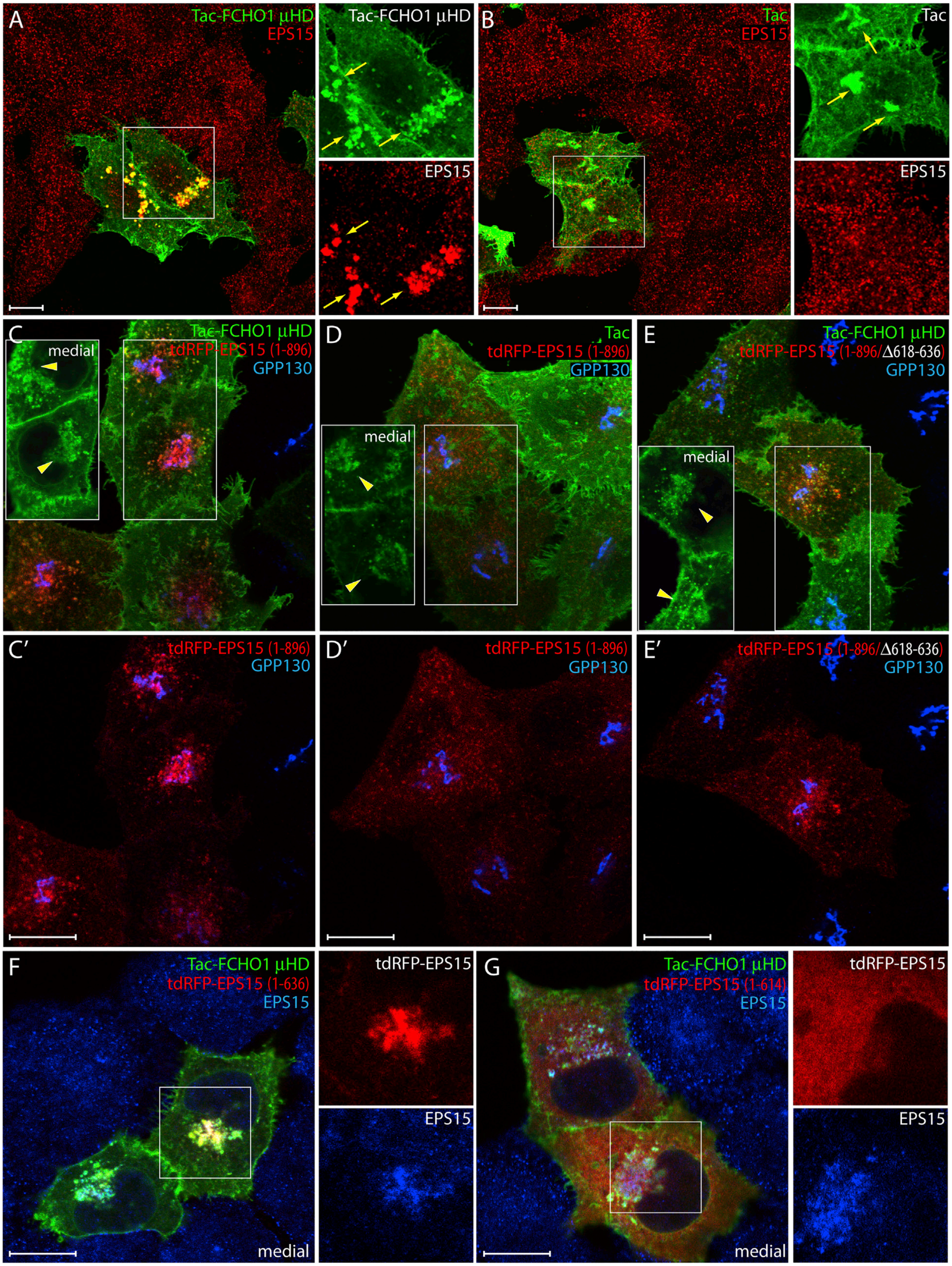

(legend on next page)

protein (Umasankar et al., 2014). Transmembrane Tac, the α chain of the interleukin-2 receptor also termed CD25, normally progresses through the biosynthetic pathway before residing at the plasma membrane (Humphrey et al., 1993). When overexpressed, a newly synthesized pool of Tac is typically seen in the Golgi en route to the surface. In Tac-μHD-, but not Tac-expressing cells, endogenous EPS15 relocates prominently to the juxtanuclear Golgi region where the cytosol-oriented μHD is highly concentrated (Figures 2A and 2B). This Tac-μHD can act as a near-quantitative sink for endogenous EPS15 in highly expressing cells, depleting EPS15 from CCSs (Figures 2A and 2C). The short DPF tract between EPS15 residues 615 and 636 promotes most effective translocation of a transfected tdRFP-full-length EPS15 onto Tac-μHD-rich membranes in the Golgi region (Figures 2C–2E). Similar ectopic concentration of the RFP-tagged full-length EPS15 does not occur upon co-transfection with Tac alone (although a pool of Tac also concentrates in the Golgi area) and is considerably reduced upon internal deletion of residues 615–636 within the tdRFP-EPS15 reporter (Figures 2D and 2E). Comparing the intracellular positioning of transfected tdRFP-EPS15 that is truncated after residue 636 with the endogenous EPS15 shows the atypical deposition of both proteins at Tac-μHD-positive organelles within transfected cells (Figure 2F). Further C-terminal truncation of tdRFP-tagged EPS15 at residue 615 produces little clustering in the juxtanuclear area, despite redistribution of endogenous EPS15 by ectopic Tac-μHD in the same cells (Figure 2G). Altogether, the results emphasize the key contribution of the mapped EPS15 DPF stretch to μHD engagement intracellularly, and suggest that these interactions play a key role in clustering these EFC-domain proteins at clathrin buds.

Molecular Architecture of the μHD Interaction Interface

To locate the molecular surface of the μHD that physically contacts Eps15, we attempted crystallization of μHDs from several species, both alone and in complex with EPS15 peptides. When all efforts failed, the tailored chimeras (Figure 1F) were evaluated. A fusion of EPS15 (designated ^{E}T615-^{E}G637) to the zebrafish Fcho1 μHD (residues 867–1,152) with a GAGA spacer crystallized and diffracted to 2.4 Å. The structure was solved by single-wavelength anomalous dispersion using an Xe derivative (Table S1). In the final structure, excellent-quality electron density is visible for residues ^{E}F620-^{E}G637 of EPS15 (Figure S2A) and P874-L1152 of the μHD. There is no evidence of the spacer residues separating the EPS15 and μHD sections, but distance constraints make it highly likely that the interaction is intramolecular.

Like other μ subunits, the Fcho1 μHD contains 18 β strands arranged as two intercalated β-sandwich subdomains, the fold being comparable with the orthologous *Saccharomyces cerevisiae* Syp1p μHD (Reider et al., 2009) and μ1–μ4 (Figures S2A–S2F). Yet the Fcho1 μHD also contains three additional α helices in subdomain A (Figures S2A–S2C), and the concave face of the Fcho1 μHD is considerably more curved than other μHDs. The EPS15 segment binds in a spiral trough that runs halfway around the long axis of subdomain A, with the Fcho μHD-unique α helices forming a considerable part of the trough (Figure 3A). The trough is lined with conserved largely hydrophobic residues, and significant contacts with the protein are only through the three DPF motif side chains of the sequence ^{623}DPFVGSDPFKDDPF (Figures 3A–3D); the interaction buries 1,640 Å^2 of solvent-accessible area. The electron density indicates well-ordered DPFs; the first two adopt type I tight turn conformations (Figures 3D and S2A) stabilized by intramolecular H bonding between the Asp residue side chains and the backbone amide of the +2-position Phe residues. In the last DPF, the side chain of ^{E}D634 additionally forms a salt bridge with R1133. In this way, the ordered triple-DPF motif projects a stabilized contiguous hydrophobic surface into the trough with the Pro residues orienting the Phe side chains to closely match the complementary μHD interaction surface (Figure 3D). Thus the structure strongly suggests that the short tri- (VGS) or di- (KD) residue spacing between tandem DPFs is important to allow three to simultaneously dock onto a single Fcho1 subdomain A with a 1:1 stoichiometry. The weakest EPS15 peptide electron density is around G627, reflecting inherent mobility of non-chiral G residues and suggesting it may be a flexible pivot. Our interpretation is that, depending on the binding partner, the trough can be either fully or partially occupied. The site is therefore "plastic," with partners evolving appropriate strength interactions by filling different portions of the extended trough. This contrasts with other interactions in vesicle coat formation whereby a small binding cavity on a folded protein accommodates a single small motif, and variations in affinity occur through different motif copy numbers. Below, we use the human FCHO1 μHD to confirm the binding mechanism (substantiating that the interaction surface in the structure is not an artifact of using the chimera); zebrafish and human Fcho1 μHDs are 56% identical and bind EPS15 with similar K_Ds (Figure 1), but FCHO1 μHD has better biochemical behavior. Residue numbers that follow are thus for the equivalent human μHD (Figure S2B).

Structure-guided mutation of FCHO1 μHD residues located near the N-terminal region of the docked EPS15 peptide verifies the role of the binding trough. A number of single substitutions in this general area each have only slightly decreased binding properties (Figures 3 and S3), indicating that multiple alterations to the extended contact interface upon the μHD are necessary to

Figure 2. The EPS15 μHD Interaction Motif In Vivo

(A and B) Representative single confocal optical sections of basal surface of HeLa SS6 cells transfected with either Tac-FCHO1 μHD (A) or Tac (B) as indicated. Fixed cells were stained with anti-Tac or anti-EPS15 antibodies. Enlarged color-separated regions corresponding to the boxed areas are shown and Golgi-localized Tac and EPS15 (arrows) are indicated. Scale bar, 10 μm.

(C–E′) Typical basal confocal sections of fixed HeLa cells double transfected with the indicated Tac and tdRFP-EPS15 plasmids and stained with antibodies against Tac and GPP130 as indicated (C′–E′). Corresponding medial optical sections of the boxed regions are shown (C–E) indicating the Golgi pool (arrowheads) of Tac protein. Scale bar, 10 μm.

(F and G) Representative medial confocal optical sections of HeLa cells transiently transfected with Tac-FCHO1 μHD and either tdRFP-EPS15 (1–636) or (1–614) as indicated after fixation and staining with anti-Tac and anti-Eps15 antibodies. Enlargements of boxes as in (A). Note that the anti-Eps15 is a peptide-specific antibody raised against the extreme C terminus of the protein, which is truncated in the two tagged exogenous proteins. Scale bar, 10 μm.

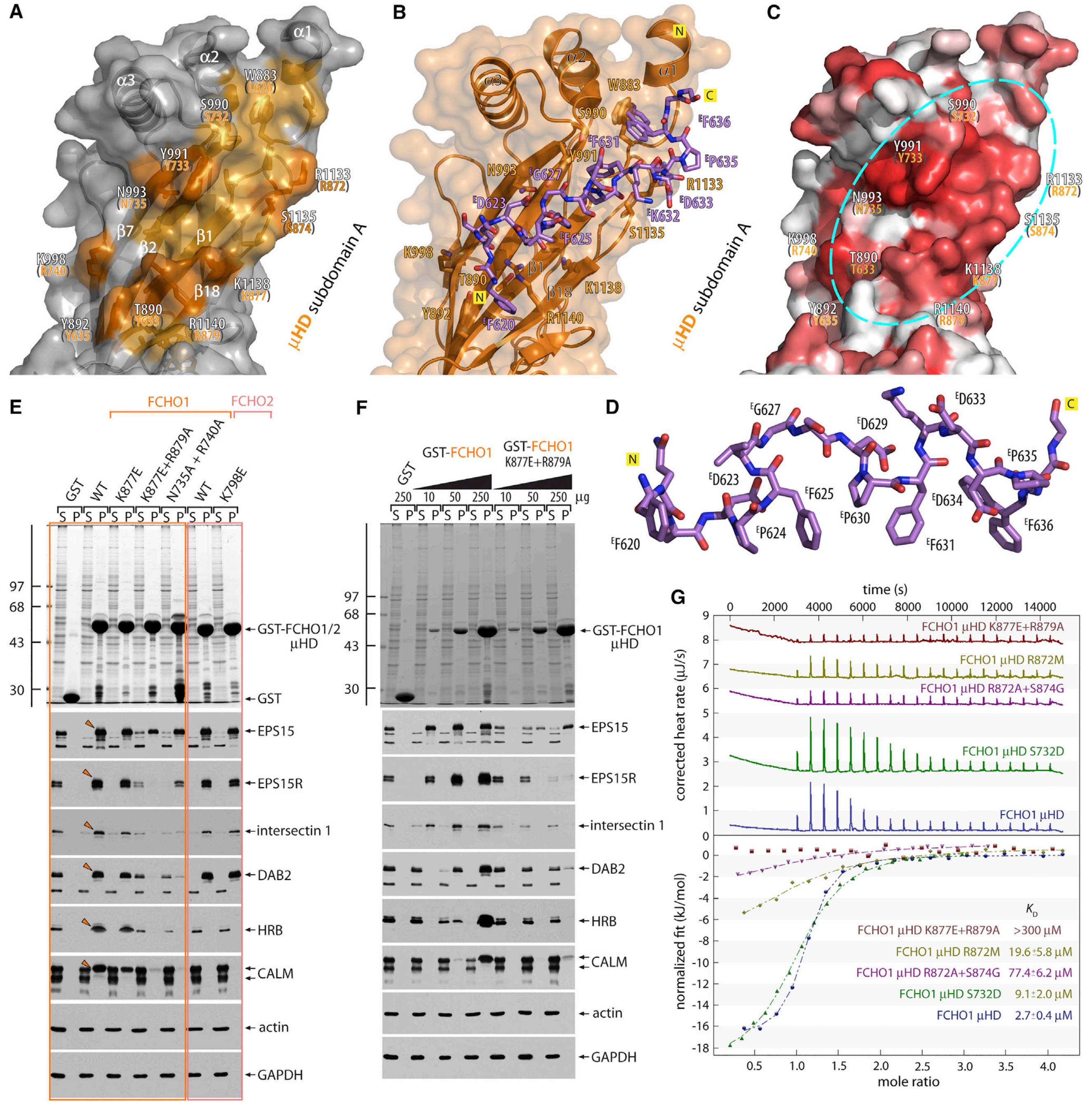

Figure 3. A Major Interaction Surface on Fcho1 μHD Subdomain A

(A–C) Combination ribbon and molecular surface representation of the concave face of zebrafish Fcho1 μHD subdomain A showing the EPS15 binding trough. (A) The β strands involved and projecting μHD contact residues (light orange), determined by the PISA server. Selected side chains mutated are indicated (dark orange) with the corresponding residue number in the FCHO1 μHD indicated in orange type. (B) View of the tandem DFP motifs (stick representation with carbon mauve, nitrogen blue, and oxygen atoms red) bound to the μHD. ^{E}F636 is shown in a dual conformation with μHD W833 also in dual conformation to permit flipping of ^{E}F636. (C) ENDscript 2 (Robert and Gouet, 2014) computed phylogenetic surface conservation between muniscin μHDs using the TrEMBL (opisthokonta) database for sequence alignments. Conservation graded in shades from invariant (red) to unrelated (white) projected onto the solvent-accessible molecular surface of the Fcho1 μHD. The surface conservation reveals a patch (cyan oval) of invariant and highly conserved residues at the binding site on subdomain A, while there is a rather discombobulated patchwork pattern of conservation over the convex face.

(D) The bound DPF tract showing the spatial arrangement of the aromatic Phe and Pro side chains packing into the μHD trough.

(E) Pull-down assay utilizing HeLa cell lysate and 250 μg of GST, GST-FCHO1 (orange box), or GST-FCHO2 (pink box) μHD, or the indicated mutant. Stained gel and replicate blots were probed with the indicated antibodies. Position of bound partner protein (arrowheads) is indicated.

(F) Pull-down assay with HeLa cell lysate and the indicated amount of GST, GST-FCHO1 μHD, or K877E + R879A mutant immobilized on glutathione-Sepharose.

(G) Representative ITC experiments of wild-type FCHO1 or color-coded mutant μHDs binding to an EPS15 616–638 peptide.

effectively disrupt binding. For compound double mutants in this part of subdomain A, the most detrimental is a K877E + R879A substitution at the outer rim of the trough. Here, association of all weak binding partners is abolished and the recovery of the strongest binding partner (native EPS15 dimer/tetramer) is reduced at least 25-fold (Figures 3E and 3F). ITC (Figure 3G) reveals binding to EPS15 peptide (616–638) is reduced to undetectable levels (K_D > 300 μM versus 2.7 μM for wild-type μHD). Simultaneous alteration of the nearby conserved residues N735A + R740A has a different and weaker inhibitory effect: durable EPS15/R binding still persists, but HRB and CALM associations are lost (Figure 3E).

Subdomain A mutations in the α-helical region located close to the second $DPFX_2DPF$ repeat again affect binding (Figures S3A and S3B). Besides a Y733A substitution, which is insoluble (all others were correctly folded judged by gel filtration and circular dichroism), a P627D + A629E switch is most detrimental to all cytosolic binding partners in pull-down assays. ITC using the EPS15 (616–638) peptide shows essentially no (K_D > 300 μM) binding for the A629E or P627D + A629E substitutions (Figure S3C). Other nearby substitutions, such as S732D and R872A + S874G, produce weaker inhibitory effects, most conspicuously on EPS15/R. In sum, the effects of the various structure-guided substitutions show that different partners occupy a common extended μHD surface but display different binding affinities, likely related to occupancy of different portions of the trough, and that Eps15 engagement involves higher-order decoding of adjacent DPF motifs filling the trough.

Several binding-trough amino acids differ in the Fcho2 and Sgip1 μHDs; the tripeptide ^{633}TXY in strand β1 at the base of the trough is [TS]X[ST] in Fcho2 and Sgip1 orthologs (Figure S3D). Yet the noted selectivity between FCHO1 and FCHO2/Sgip1 for HRB and CALM binding (Figure S1E) cannot be solely attributed to this Y635T/S dichotomy. An FCHO1 Y635S alteration does not prevent binding of either HRB or CALM (Figure S3E). The adjacent T633Y substitution produces a modest gain-of-function effect for the weaker partners, HRB and CALM. These results illustrate that varied μHD partner motifs have evolved not an optimal but rather an acceptable fit, given the temporal and spatial requirements of these interactions in vivo.

The muniscin μHD, AP μ subunits, and δ-COP (Suckling et al., 2015) μHD all evolved from the TCUP subunit of the ancestral TSET/TPLATE sorting complex (Gadeyne et al., 2014; Hirst et al., 2014). Our work shows that in addition to conservation of the overall architectural fold, the general locale of the main peptide contact surfaces on the concave face of subdomain A is broadly similar but the molecular mechanism of peptide binding is different (Figure S2); the muniscin interaction is more expansive and is not of a β-augmentation type.

μHD Interactions In Vivo

Unfortunately the functional role of the μHD cannot be explored simply in cells by direct deletion or mutation of full-length proteins, because forced overexpression of the Fcho1/2 EFC+ linker augments CME by promoting AP-2 opening (Hollopeter et al., 2014; Umasankar et al., 2014). The unstructured interdomain linkers of Fcho1, Fcho2, and Sgip1 all bind physically to the heterotetrameric AP-2 adaptor core, and this biochemical activity is correlated with improved AP-2-dependent cargo uptake (Hollopeter et al., 2014; Umasankar et al., 2014). When expressed ectopically in HeLa cells as a Tac-fusion protein, the FCHO1 linker alone improves transferrin internalization (Umasankar et al., 2014). Paradoxically, these results make it appear superficially as if the μHD is functionally unnecessary. However, of all the pioneers the muniscins are present as limiting components (Figure S1I); overexpression studies evade this level of regulation by copy number. As outlined below, we hypothesize that it is precisely this low abundance of FCHO1/2 that makes the concerted transactions between the μHD and the interdomain linker physiologically relevant.

Unlike the Tac-μHD (Figure S4A), a K877E + R879A mutation prohibits overexpressed Tac-μHD from abnormally clustering EPS15 in the juxtanuclear region in transfected HeLa cells; EPS15 localization is much like control Tac-transfected cells (Figures S4B and S4C). Of all the cytosolic proteins that bind to the μHD in pulldowns, only EPS15 and HRB prominently change intracellular placement in the presence of wild-type Tac-μHD, although some HRB surface puncta remain. Intersectin 1, DAB2, and CALM do not show pronounced changes in plasma membrane surface deposition compared with cells expressing Tac only (Figures S4A–S4F). This could reflect the densely redundant protein-protein and protein-lipid interaction networks these other μHD partner CLASPs display at surface CCSs (Figure S1I).

Ectopic μHD Expression in FCHO1/2-Null Cells

To examine this further, we overexpressed Tac-μHD in HeLa clone 1.E cells. This genome-edited clone is functionally FCHO1+2 null and displays clustered, enlarged surface CCSs at steady state (Umasankar et al., 2014). In 1.E cells, ectopic Tac-μHD again causes strong mislocalization of EPS15 and HRB (Figure 4A). Yet intersectin 1, DAB2, and CALM still do not mass prominently in the Golgi region when Tac-μHD is expressed (Figures 4B–4E). Regardless, the ectopic Tac-μHD strikingly alters endocytic CCS appearance in the muniscin-null cells; different to adjacent non-transfected 1.E cells, the Tac-μHD producers (identified by Tac staining and/or Golgi mislocalized EPS15 or HRB) exhibit small, dim, and generally dispersed AP-2-positive structures (Figure 4C). AP-2, intersectin 1, DAB2, CALM, and HRB, which co-populate most enlarged CCSs in parental 1.E cells, spatially segregate into non-overlapping spots when wild-type Tac-μHD is present (Figures 4B–4E). The scattered appearance of these coat components in the presence of membrane-anchored μHD resembles the population of smallest CCSs, increased in frequency in parental 1.E cells (Umasankar et al., 2014). Analogous alterations to AP-2-positive puncta occur when overexpressed FCHO1 μHD is instead targeted to endosomes by fusion to paired FYVE domains (Figure 4F). Again, massive intracellular EPS15 sequestration occurs and surface AP-2 puncta diminish in size and intensity. The μHD of Sgip1, which does not interact directly with either HRB or CALM (Figure S1E), also causes a reorganization of surface AP-2 when overexpressed in HeLa 1.E cells as a Tac-fusion protein (Figures S5A–S5F). Under our experimental conditions, we cannot completely exclude that the ectopic Tac-μHD we use to move EPS15 and EPS15R onto intracellular membranes does not alter subtly the precise location or dynamics of the cytosolic pools of

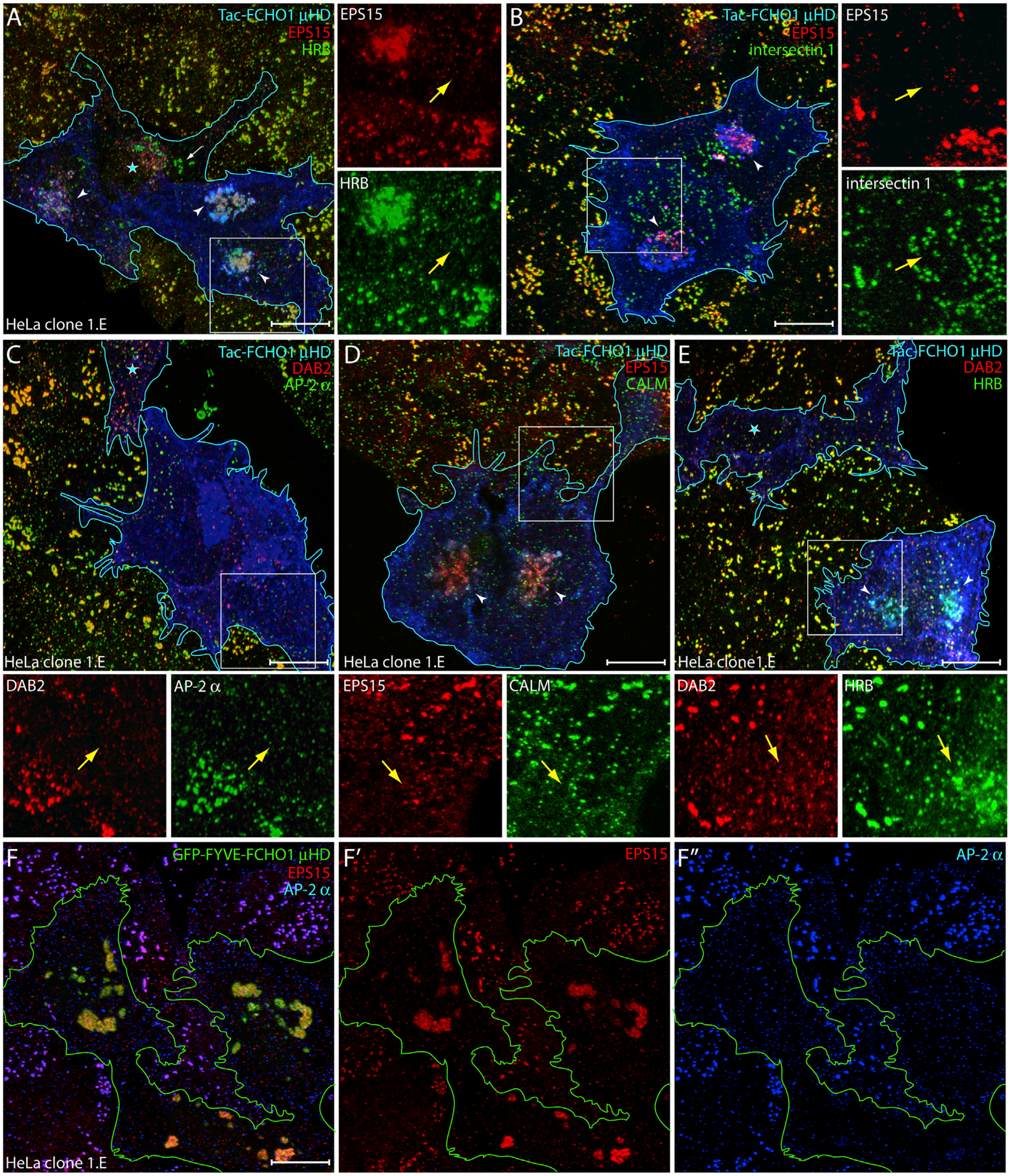

Figure 4. Altered CCSs in Tac-μHD-Transfected HeLa 1.E Cells

(A–E) Deconvolved maximal projections of HeLa clone 1.E cells transfected with Tac-μHD and stained with the indicated antibody combinations. Transfected cells (blue outline), misrecruited juxtanuclear EPS15 and/or HRB (arrowheads), and altered pioneer/CLASP distributions (yellow arrows) are indicated. Sequestration is dose dependent; residual HRB-positive enlarged CCSs (white arrow) in a low Tac-μHD expresser (asterisk) are indicated in (A). Scale bar, 10 μm.

(F–F″) Representative deconvolved maximal projection of GFP-FYVE-μHD transfected (green outline) HeLa 1.E cells stained with the indicated antibodies. Scale bar, 10 μm.

other μHD-binding partners, including DAB2, HRB, and CALM. But even were this the case, it still attests to the fundamental importance of the μHD interaction surface we delineate here in coordinating protein-protein contacts at nascent bud sites. That EPS15 misrecruitment is not accompanied by large-scale intracellular deposition of AP-2 also reinforces that the different affinities that μHDs and AP-2 appendages display for DPFs also manifest in vivo, and makes plain the requirement for an appropriate acceptor membrane to promote AP-2 switching from the cytosolic closed state to the membrane-docked, cargo-binding-competent open state.

The phenotypic effect of combined siRNA silencing of *EPS15* and *EPS15R* transcripts in clone 1.E cells is also consistent with a direct role for EPS15/R in stabilizing nascent AP-2 assemblies at the cell surface; in cells with strongly diminished EPS15 there is a shift in the distribution of AP-2 puncta to the smallest-size populations (Figures S5G–S5L). Our results thus suggest that in the combined absence of muniscins and EPS15/R, a lack of coat consolidation/stabilization follows random clathrin-coat component encounters with the plasma membrane, accounting for the small dim puncta. Because neither intersectin, CALM, DAB2, nor AP-2 accumulates on concentrated intracellular Tac-μHD, this tactic to selectively mislocalize EPS15 (and HRB) in muniscin-null cells allows further functional dissection of the EPS15/R•μHD interaction in CME using transferrin uptake assays and total internal reflection fluorescence microscopy (TIRFM).

Impact of μHD Interactions on CME

First, we assessed ligand uptake. On a 2-min pulse, fluorescently labeled transferrin clusters at, and is internalized from, abnormally enlarged CCSs in parental 1.E cells. Obviously different from this surrounding untransfected 1.E cell population, the Tac-μHD transfected 1.E cells, which display bright intracellular EPS15 staining and dispersed small faint plasma membrane dots of AP-2, accumulate transferrin diffusely over the plasma membrane, and uptake into endosomes is strongly slowed (Figures S6A–S6A‴), indicating defective CME. Even after 10 min of incubation with transferrin, Tac-μHD-producing cells with dim AP-2 spots internalize very little cargo relative to neighboring untransfected 1.E cells with typically large CCSs (Figures S6B–S6B‴). Thus the fine AP-2 puncta at the surface do not efficiently package the prototypical YXXØ-type signal cargo protein, the transferrin receptor when FCHO1/2 and EPS15 are limiting (i.e., depleted from the vicinity of the plasma membrane). These results are in stark contrast to our previous findings regarding the effect of transfecting the same FCHO1/2-null 1.E HeLa cells with Tac fused instead to the preceding FCHO1 interdomain linker (residues 265–609) (Umasankar et al., 2014). This disordered segment of FCHO1 between the folded EFC domain and μHD also alters the steady-state morphology of CCSs to small regular puncta, but in this case they are brighter (contain more AP-2) and transferrin uptake is actually stimulated (Figures S6C–S6C‴) as the linker on the plasma membrane can trigger AP-2 to adopt an open, cargo-binding conformation (Hollopeter et al., 2014; Umasankar et al., 2014). In fact, in *Caenorhabditis elegans*, numerous *fcho-1*-null bypass suppressor mutations in AP-2 subunits, which shift the equilibrium of AP-2 to the open state, completely negate any requirement for FCHO-1 in vivo (Hollopeter et al., 2014).

Definitive evidence for defective CME comes from cells pulsed with transferrin at 37°C for 2 min, surface stripped on ice, and reheated to 37°C for an additional 2 min. The peripheral transferrin-positive early endosomes abundant in surrounding untransfected 1.E cells are diminished in Tac-μHD-producing 1.E cells that also exhibit altered AP-2 spots (Figures 5A and S6D). By comparison, 1.E cells expressing Tac-μHD (K877E + R879A) have transferrin and AP-2 staining roughly comparable with adjacent untransfected cells (Figure 5B). In transferrin-pulsed 1.E cells labeled for AP-2 and also for EPS15 to observe internal sequestration of this pioneer by Tac-μHD, transferrin uptake is sharply reduced (Figure 5C). By contrast, Tac-linker-positive cells have a characteristic changed AP-2 arrangement, and more numerous and medial transferrin-positive endosomes (Figures 5D and S6D). The observed AP-2 puncta in either Tac-μHD- or Tac-linker-producing 1.E cells are therefore functionally distinct, with the explanation for the opposite outcomes on transferrin internalization being that the Tac-μHD exerts a dominant sequestration effect on EPS15 at intracellular membranes while the Tac-linker works by gain of function on AP-2 at the plasma membrane (Umasankar et al., 2014).

The AP-2 puncta in Tac-μHD-expressing 1.E cells appear similar to the small abortive CCSs that arise in cells expressing AP-2 lacking the α-subunit appendage (Aguet et al., 2013), which binds directly to single EPS15 DPFs among other ligands (Brett et al., 2002; Praefcke et al., 2004). However, marked surface transferrin accumulation shows that Tac-μHD-transfected HeLa 1.E cells do not display compensatory CME, like cells with endogenous Eps15 and Fcho1/2 (Aguet et al., 2013; Motley et al., 2006). Indeed, time-resolved imaging of β2-YFP-marked AP-2 puncta on the bottom of FCHO2-null HeLa cells (clone #46β, Figures S7A–S7E) transfected with Tac-μHD reveals highly dynamic, dim, short-lived (presumably unproductive or abortive) spots (Figures 6A and 6C), explaining the accumulating transferrin at the plasma membrane. For these live-cell studies we used the FCHO2 gene-disrupted HeLa clone #46β cells because FCHO1 protein levels are extremely low in HeLa cells (Hein et al., 2015; Umasankar et al., 2012) and the endocytic phenotype and behavior of FCHO2-null and FCHO1/2-null HeLa cells are indistinguishable (Umasankar et al., 2014). Similarly to HeLa and clone 1.E cells, ectopic Tac-μHD redirects endogenous EPS15 to a perinuclear location in the clone #46β cells (Figures S7F and S7G). The time-resolved imaging experiments show importantly that defective diminutive AP-2 puncta occur in living Tac-μHD-producing cells and that they display abnormal kinetic behavior (Figures 6C and 6D). In parental #46β cells, 75% of patch lifetimes are >108 s (median spot duration ~294 s). When expressing the Tac-μHD, the median spot duration in these cells becomes ~30 s compared with ~74 s for cells expressing the Tac-linker (Figure 6D). The kinetic behavior (median duration ~75 s) and area distributions of AP-2 puncta in Tac-linker expressing clone #46β cells closely resemble HeLa SS6 β2-YFP cells, from which the #46β cells were derived (Figures 6D–6G). Collectively these experiments show that, alone, the Fcho μHD or Fcho linker can change the morphology, kinetic signature, and cargo-sorting capacity of surface CCSs in muniscin-null cells. Since the interdomain linkers of Fcho2 and

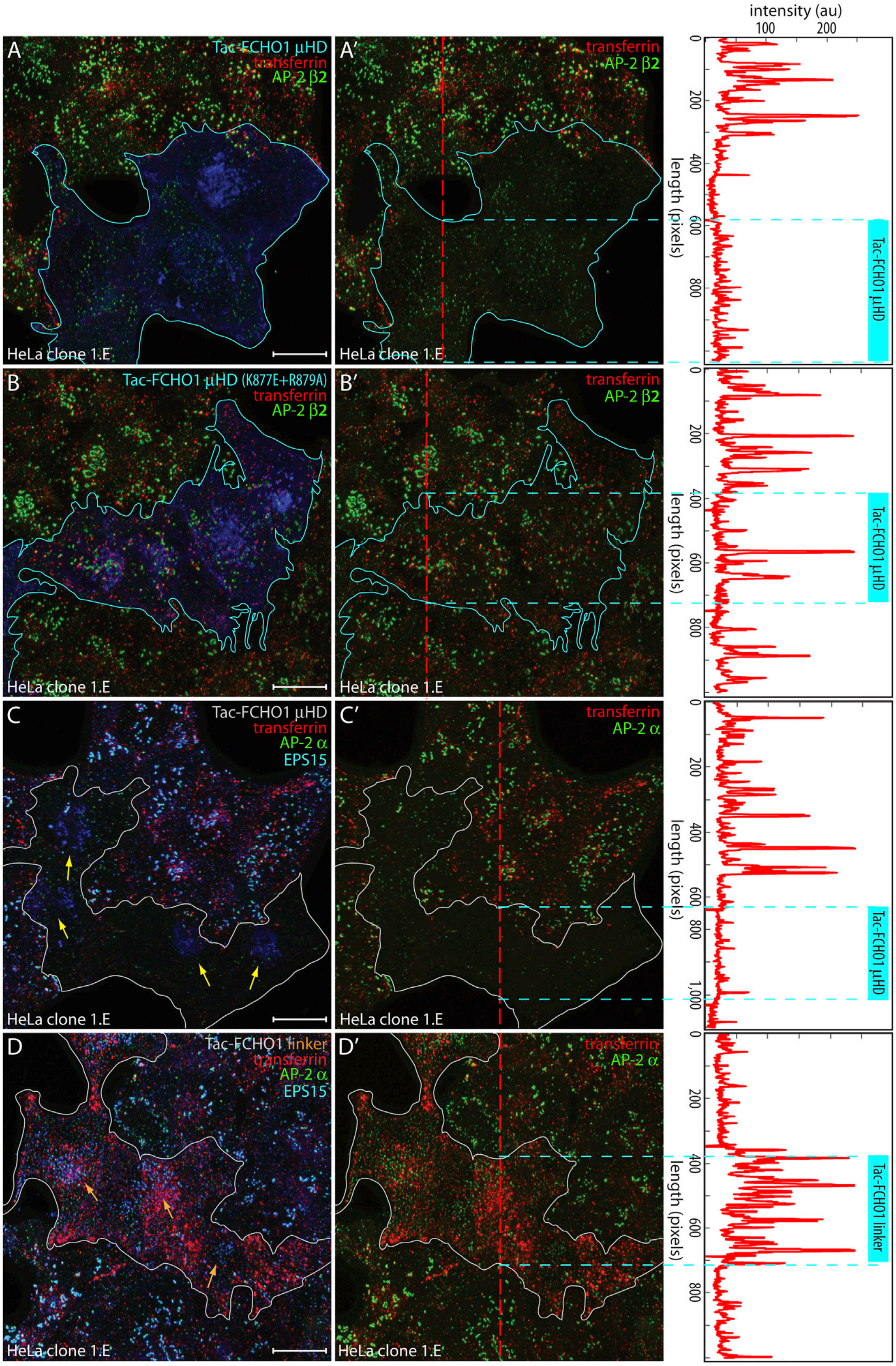

(legend on next page)

Sgip1 also affect AP-2 complex conformation (Hollopeter et al., 2014; Umasankar et al., 2014), our findings underscore the functional adaptation and importance of sequentially arranged regions of the low-abundance muniscins (Figure S1I). In cells, we propose that the linked muniscin domains act on AP-2 in concert by virtue of the protein-binding properties of the μHD; they bring together the necessary protein machinery to switch AP-2 from the basal closed conformational state to the open active form.

DISCUSSION

This work explores how interactions between Fcho1/2, Eps15/R, and AP-2 contribute to CCS nucleation. While Eps15 engages Fcho1/2 and AP-2 in fundamentally different and noncompeting ways, both intriguingly depend on DPF triplet-based recognition (Brett et al., 2002; Praefcke et al., 2004). Within the Eps15 unstructured region, residues 600–650 contain seven DPF repeats that, with limiting spacing requirements, bind Fcho1/2 as a higher-level triple-DPF motif. AP-2 binding to distal Eps15 residues 650–740 (Benmerah et al., 1996; Iannolo et al., 1997) depends on only single DPF motifs (Brett et al., 2002; Praefcke et al., 2004). Relatively tight apparent binding is due to avidity phenomena: multiple α appendages from independent AP-2 heterotetramers bind to the array of single DPFs (Praefcke et al., 2004) on the Eps15 dimer/tetramer (Cupers et al., 1997), and is further enhanced by the AP-2 β2 appendage also binding Eps15, albeit through a different Phe-rich motif (Edeling et al., 2006; Schmid et al., 2006). While this synchronous binding of separate DPFs to individual AP-2 appendages is facilitated by unstructured intervening peptide stretches between DPFs, strict inter-motif intervals are unimportant. Consequently, spacing constraints have allowed Eps15/R to separately tune DPF-binding selectivities for μHDs and AP-2 appendages (Figure 7A). The two proteins bind independently of one another; docking of a triple-DPF motif onto a μHD does not affect the remaining unstructured Eps15 C terminus, leaving it free to engage one or more AP-2 adaptors. The tighter binding and slower off-rate of a triple-DPF•μHD interaction ($K_D \cong 3$ μM) compared with a single DPF•α-appendage interaction ($K_D \sim 200$ μM) (Olesen et al., 2008) favors initial Fcho1/2•Eps15/R heterodimer formation (Figure 7A). On the plasma membrane, at a forming CCS, where clustered AP-2 is bound with low micromolar affinity and oriented by PtdIns(4,5)P_2, the high off-rate of a lone DPF from an appendage is less critical; fast rebinding would occur, and since multiple appendages can simultaneously be engaged by the distal Eps15/R C termini of Eps15 oligomers, Fcho1/2•Eps15/R•AP2 nanocluster formation is favored.

An Endocytic CCS Commencement Model

In HeLa clone 1.E cells lacking muniscins, EPS15/R can direct the assembly of stable, enlarged, long-lived CCSs with delayed CME (Umasankar et al., 2014). Silencing of EPS15/R (Teckchandani et al., 2012) or reconstitution of knocked-down AP-2 cells with an α-appendageless AP-2 heterotetramer (Aguet et al., 2013) similarly causes enlarged, clustered, and functionally abnormal CCSs. We now show that without both FCHO1/2 and EPS15/R, CCSs are short-lived and CME is further disrupted. This information points to an interdependent three-way relationship between muniscins, Eps15/R, and AP-2. So how does the deciphered DPF code support productive CCS formation? Extinguishing clathrin expression in HeLa cells by RNAi blocks CME, but has little impact on deposition of a planar AP-2, EPS15, and CALM matrix on the plasma membrane (Hinrichsen et al., 2003; Meyerholz et al., 2005; Motley et al., 2003). This indicates that adaptors/CLASPs require clathrin, in all probability by clustering CLASPs to high local density, for proper membrane deformation, but that initial congregation of pioneers at emerging buds is largely clathrin independent (Hinrichsen et al., 2006). By contrast, AP-2 transcript silencing leads to diminutive CCSs (Miller et al., 2015; Motley et al., 2003). One interpretation of this is that AP-2 deposition at nascent assembly zones expands the developing coat by loading the interior of a pioneer-rich patch, with open AP-2 able to engage cargo (Figure 7B). How would this happen? AP-2, Eps15/R, and Fcho/2 differ considerably in projected copy number/cell (Hein et al., 2015) and, hence, attainable stoichiometry at individual assembly zones (Borner et al., 2012). When an EFC domain docks on the negatively charged plasma membrane (Umasankar et al., 2012), Fcho1/2•Eps15/R complexes (Figure 7B, step 1) could sequentially bind to (step 2) and then deposit AP-2 adaptors in the immediate vicinity, aided by interaction of the AP-2 α/β2-subunit trunks with plasma membrane PtdIns(4,5)P_2. A local but temporary DPF-mediated three-component nanocluster would allow AP-2 to linger in the vicinity of Fcho1/2 to advance CCS assembly by encounters with the muniscin effector linker (step 3). This interaction leads to conformational rearrangement of AP-2 to an "open" or active form (Hollopeter et al., 2014; Umasankar et al., 2014) that facilitates cargo engagement (step 4) and release of the AP-2 β2-subunit hinge to promote clathrin polymerization (step 5). In this way, a large increase in AP-2 dwell time at the forming CCS results.

This scenario is grounded on the observations that despite retaining PtdIns(4,5)P_2 binding, AP-2 without an α appendage is only weakly plasma membrane associated (Robinson, 1993); that overexpression of an N-terminally-deleted Eps15 mutant lacking EH domains 2 and 3 mislocalizes AP-2 and inhibits

Figure 5. Defective CME in Tac-μHD-Expressing 1.E Cells

(A–B′) HeLa clone 1.E cells transfected with either Tac-μHD (A) or the Tac-μHD (K877E + R879A) mutant (B) and pulsed with transferrin (red) for 2 min at 37°C, surface stripped on ice, and rewarmed to 37°C for another 2 min before fixation. Representative deconvolved maximal intensity z projection of permeabilized cells stained with antibodies against Tac and the AP-2 β2 subunit is indicated. Transfected cell groups are outlined, and superimposed transferrin and AP-2 shown in (A′) and (B′). Scale bar, 10 μm. Correlative linescan analysis at indicated locations in A′ and B′ (dashed vertical line) is shown on the right, with relative position of transfected cells indicated with a vertical cyan bar and horizontal broken cyan lines.

(C–D′) Deconvolved z stack projections of 1.E cells transfected with either Tac-μHD (C) or Tac-linker (D) and similarly pulsed with transferrin as in (A) and (B). Fixed and permeabilized cells were stained with antibodies against the AP-2 α subunit and EPS15 as indicated. Tac-μHD-dependent intracellular clustering of EPS15 (yellow arrows) and diminished transferrin internalization contrasts the retention of EPS15 in regular AP-2-positive surface puncta (orange arrows) and enhanced endocytosis in presence of the Tac-linker. Scale bar, 10 μm. Correlative linescan analysis at indicated locations in (C′) and (D′) (red dashed line) is shown on the right, with relative position of transfected cells indicated as in (A) and (B).

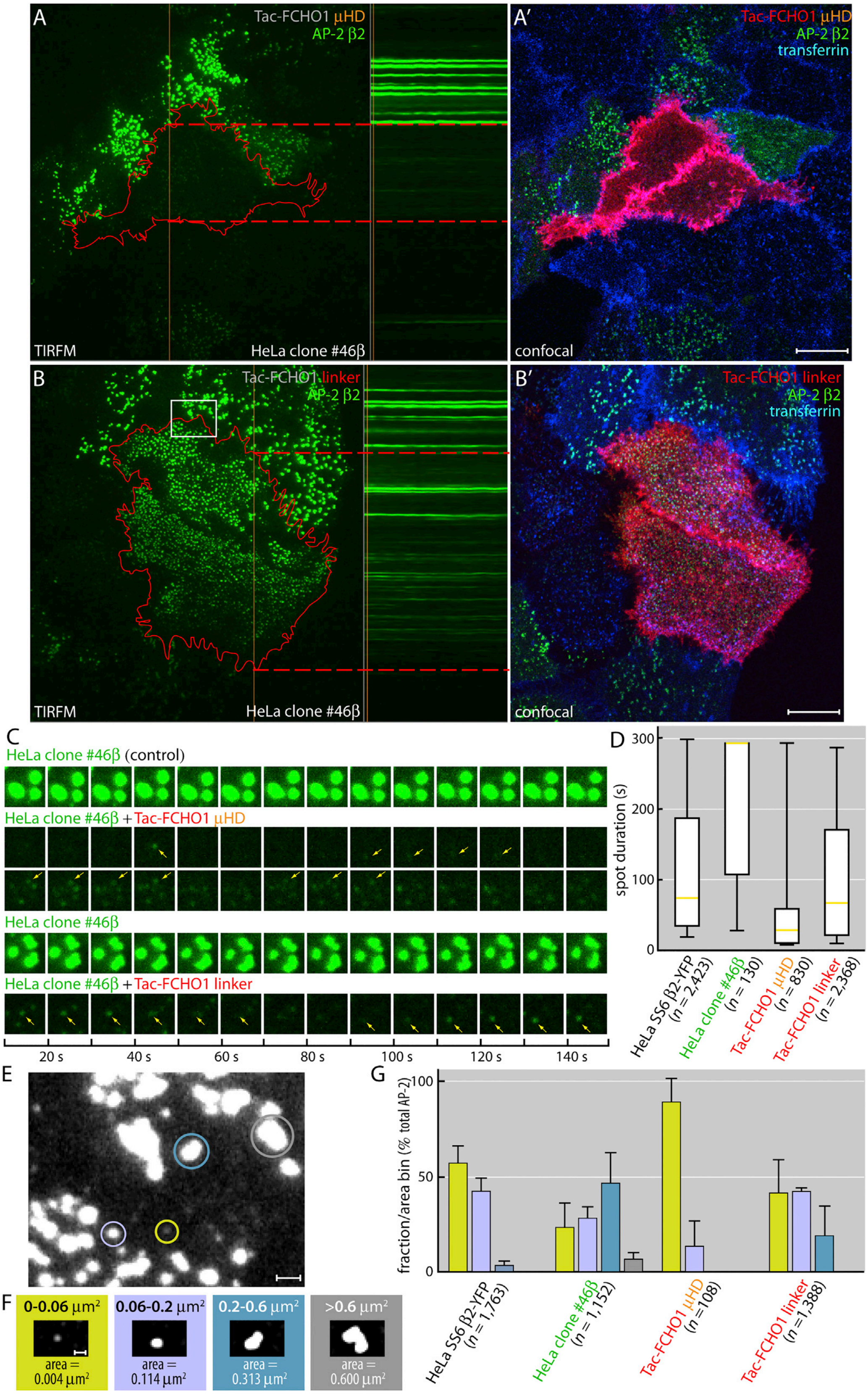

(legend on next page)

CME (Benmerah et al., 1999; Carbone et al., 1997); and that microinjected α appendage inhibits AP-2 deposition at CCSs (Hinrichsen et al., 2006). As the local concentration of AP-2 increases at the assembly zone, other incoming adaptors/CLASPs such as Dab2, ARH, epsin, and CALM, because they are more abundant (Figure S1I) (Hein et al., 2015), can outcompete Eps15/R for appendage binding since they all also bind PtdIns(4,5)P_2, clathrin and cargo in addition to engaging AP-2 (Figure 7B). In this way, Eps15 and associated muniscins will be displaced from the center of the nascent CCS. Eps15/R and associated muniscins become positioned around the growing lattice edge while bulk AP-2 concentrates in the CCS center. Our experimental data support this hypothesis in that, for CCSs of at least moderate size in HeLa cells, FCHO2 is restricted to a band of globular proteinaceous material that we visualize surrounding assembled clathrin (Figures 7C–7E), similarly to Eps15 (Edeling et al., 2006; Henne et al., 2010; Tebar et al., 1996). Proteomic analysis also supports the model, showing that relatively little FCHO2 and EPS15/R is packaged into budded coated vesicles (Borner et al., 2012) despite their early appearance at beginning CCSs (Taylor et al., 2011). The outer rim of the forming CCS will thus delimit the perimeter and constrain productive coat polymerization to within the assembly zone. The edge effectively also conveys assembly information because remodeling of the clathrin patch, assembly of which is triggered by activation of AP-2, from an initial flat to hemispherical and then deeply invaginated profile at the center, requires polymeric clathrin (Avinoam et al., 2015; Hinrichsen et al., 2006; Miller et al., 2015; Wu et al., 2003). Finally, the proposed working model provides an explanation for the open question of how AP-2 can be massed at CCSs stoichiometrically exceeding other pioneer factors, yet depends on them in part for recruitment.

A remarkable capability of surface CCSs is to quickly produce cargo-packed transport vesicles, often in single turnover events, at variable locations on the plasma membrane. Our results and others (Mulkearns and Cooper, 2012; Teckchandani et al., 2012) reveal that although alterations in FCHO1/2:EPS15/R ratios change CCS morphology and distribution, CME does not halt under these conditions. AP-2, CALM, DAB2, and other appendage-binding CLASPs co-cluster at enlarged CCSs in FCHO1/2-null cells (Figure 4). Internalization is slowed but, given time, transmembrane cargo internalizes, substantiating that Fchos guide efficient cargo-laden vesicle production. The model we propose also provides a mechanistic rationale for the previously unexplained demonstration of defective AP-2-dependent cargo uptake in *fcho-1* mutant *C. elegans* complemented with a single-copy FCHO-1 lacking the μHD (Hollopeter et al., 2014).

In sum, it is now evident that the role of the multistep (Aguet et al., 2013) initial DPF-code-mediated Fcho1/2-Eps15-AP-2-Dab2-Hrb-CALM interactome, the details of which we illuminate here, is to first prime the endocytic process and then optimize cargo packaging. As this is the principal biological imperative for clathrin-coated vesicle production, the assembly of Fcho•Eps15•AP2 nanoclusters is at the core of efficient CME.

EXPERIMENTAL PROCEDURES

Materials, Plasmids, Protein Expression, and Cell Culture

Standard techniques were used throughout. Detailed information about approaches and description of methods are presented in Supplemental Experimental Procedures. All experiments reported were repeated at least three times with similar results.

Crystallization and Structure Determination

Crystals of the EPS15-Fcho1 μHD chimera grew in hanging drops against a reservoir of 100 mM Bis-Tris propane (pH 6.0), 200 mM sodium citrate, 22% polyethylene glycol 3350, and 10 mM DTT and were cryoprotected in 22% glycerol before freezing. A Xe derivative was used for phasing (Table S1).

Biochemical Assays

Pull-down assays entail mixing glutathione-Sepharose-immobilized GST-fusion proteins with a cell extract or purified proteins, washing, and analysis of resulting supernatant and pellet fractions. For SDS-PAGE and blots, five times more of each pellet versus supernatant fraction was loaded. ITC involves repeated injection of EPS15 peptides into a temperature-controlled solution of μHD utilizing a Nano ITC instrument (TA Instruments).

Endocytosis Assays

Prior to transferrin addition, HeLa cells were incubated for 60 min at 37°C in a starvation medium of DMEM supplemented with 25 mM HEPES and 0.5% BSA of to clear receptors of apotransferrin. Fluorescently labeled Alexa Fluor transferrin (25 μg/ml) was added in prewarmed (37°C) medium and internalization was for either 2 or 10 min at 37°C. For the transferrin pulse-chase uptake assays, starved cells were incubated with fluorescent transferrin at 37°C for 2 min and then immediately chilled on ice and washed with PBS as described by Reis et al. (2015). Surface-bound transferrin was then stripped by washing the adherent cells three times with cold 0.2 M acetic acid and 0.2 M NaCl (pH 2.5) on ice. Prewarmed DMEM containing 25 mM HEPES and 0.5% BSA was added to the stripped cells, which were then returned to a 37°C water bath for an additional 2 min before fixation.

Microscopy

Confocal imaging was on an inverted Olympus FV1000 microscope equipped with a PlanApo N (60 ×/1.42 NA) oil objective. Excitation and emission wavelengths were preprogrammed by the instrument running the FV10-ASW software, and emission signals in the different channels were collected in the sequential scan mode. Exported TIFF file images were imported into Adobe Photoshop CS4 for minor adjustments to brightness and/or contrast. Quantitation of transferrin uptake in confocal image files was performed using the linescan application within MetaMorph (Molecular Devices). The extent of

Figure 6. Uptake Defects in FCHO2 and EPS15 Compromised Cells

(A–B′) TIRFM analysis of HeLa clone #46β cells transfected with either Tac-μHD (A) or Tac-linker (B). The initial frame of the kymograph series (left), the kymograph (center), and a final confocal image (A′, B′) of the same area after labeling with anti-Tac and fluorescent transferrin (right) are shown. A single pixel-width vertical line (orange) on the first TIRFM fame (left) indicates the coordinates for the kymograph (center panel), and the relative locations of the transfected cells in the kymographs are marked (dashed horizontal red lines). Scale bar, 10 μm.

(C) Time-lapse examples of local β2-YFP puncta selected from the image series in (A) and (B) to detail the major differences between the long-lived large structures in the control clone 1.E cells compared with the transfected cells. Representative individual spot life times (arrows) are indicated.

(D) Box-and-whisker (minimum-maximum) plot quantitation of computed spot durations from the kymographs in (A) and (B), and also compared with the parental HeLa SS6 β2-YFP cells. Median is indicated in yellow and the number of fluorescent puncta analyzed/condition is indicated.

(E–G) Enlarged gray-scale view (E) of region boxed in (B) illustrating selected examples of the color-coded spot area bins (F) used in quantitation (G). The SD and number of spots analyzed for each condition are indicated. Scale bars, 1 μm (E) and 0.5 μm (F).

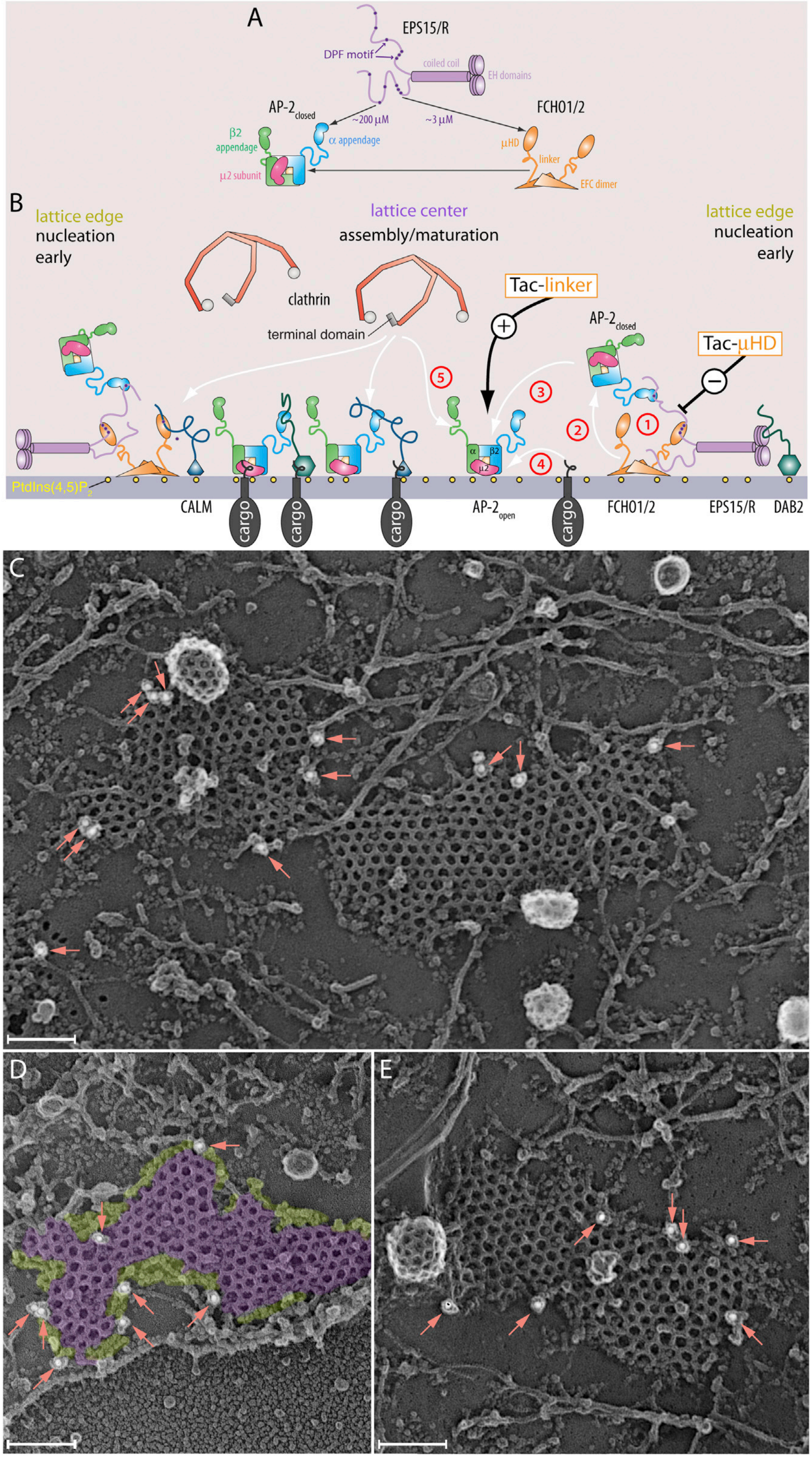

Figure 7. Transient Higher-Order Complexes to Load Active AP-2 at Growing Assembly Zones

(A and B) Schematic models for edge effects on vectorial CCS initiation. The DPF code (A) will have consequences for the formation and stability of Eps15•Fcho1/2, Eps15•AP-2, and Eps15•Fcho1/2•AP-2 nanoclusters. Because of the higher apparent affinity of the Fcho μHD for the DPFs in Eps15/R, formation of Eps15•Fcho1/2 will be favored at inchoate sites of CCS assembly.

(C–E) Representative deep-etch electron microscopic image views of adherent ventral cell membranes of HeLa SS6 cells immunogold labeled for FCHO2 with 18 nm colloidal gold (arrows). Proteins assembled around the perimeter (chartreuse) of characteristic polyhedrally assembled clathrin lattice (purple) are pseudo-colored (C). Scale bar, 100 nm.

transferrin internalization in confocal image stacks that were collected following the pulse-chase format was analyzed using Fiji software (Schindelin et al., 2012). Images were corrected for the background. A 10.09 × 10.09-μm area was selected as a region of interest (ROI) in basal, middle, and upper sections of each cell in the z stack. Each optical section image was saved and processed separately. A mask for each image was created by applying a manual threshold. The mask was then used on the corresponding ROI image to measure the number and intensity of each spot in different cells. For cells in the different populations (control untransfected, Tac-μHD, or Tac-linker expressing), the intensity values of spots in each population set were pooled together

and plotted in the histogram with the same total area (2,443.4 μm^2) for comparison.

Live-Cell Imaging

Starved cells in glass-bottomed MatTek dishes were imaged by TIRFM. Four separate positions were selected for each experiment and images were collected every 5 s for 5 min. The surface residence times of positively labeled structures were calculated using Imaris (Bitplane). The "spots" function was used to delineate AP-2 (β2-YFP)-positive structures in selected ROIs using local contrast thresholding of the TIRFM datasets. Spots were uniformly sized at slightly greater than diffraction limit (267 nm) for the 100 × 1.49 NA TIRFM objective and the wavelength of light collected. Spot persistence was tracked over time (5 s image separation) to determine duration of residency of individual structures at the cell surface. In addition, live-cell TIRFM images and immunofluorescence confocal images from fixed cells were analyzed by using Fiji and MetaMorph. Raw AP-2 channel images were background corrected. For AP-2 TIRFM datasets, 2D deconvolution was then applied to these images in MetaMorph to increase the signal-to-noise ratio. Further analysis was carried out using Fiji; individual cells were marked and saved separately. Deconvoluted images were smoothed and thresholded manually to create a mask, and masks used on the corresponding background-corrected images to calculate values for the size/area of the AP-2 puncta.

ACCESSION NUMBERS

The final coordinates and structure factors have been deposited in the PDB with accession code PDB: 5JP2.

SUPPLEMENTAL INFORMATION

Supplemental Information includes Supplemental Experimental Procedures, seven figures, and one table and can be found with this article online at http://dx.doi.org/10.1016/j.devcel.2016.05.003.

AUTHOR CONTRIBUTIONS

Conceptualization, L.M.T; Methodology, D.J.O. and L.M.T; Software, A.J.M; Validation, L.M., P.K.U., A.G.W., A.L., A.J., D.J.O., and L.M.T.; Formal Analysis, A.J.M., A.G.W., S.S.H., and S.C.W.; Investigation, L.M., P.K.U., A.G.W., A.L., A.J., A.J.M., D.J.O., S.S.H., T.P.-S., L.M.T., and S.C.W.; Resources, S.S.H., A.J., A.L., L.M., A.J.M., D.J.O., T.P.-S., L.M.T., P.K.U., S.C.W., and A.G.W.; Writing – Original Draft, D.J.O. and L.M.T.; Writing – Review & Editing, D.J.O., L.M.T., and P.K.U.; Visualization, D.J.O., L.M.T., P.K.U., and A.G.W.; Supervision, D.J.O. and L.M.T; Funding Acquisition, D.J.O. and L.M.T.

ACKNOWLEDGMENTS

We are grateful to our many colleagues, including Juan Bonifacino, Frances Brodsky, Pietro de Camilli, Adam Linstedt, and Ernst Ungewickell, for generously sharing important reagents, and to Robyn Roth for her amazing mastery of freeze-etch. We thank the beamline scientists at Diamond Light Source and Phil Evans for assistance. Supported by NIH R01 GM106963 to L.M.T. and Wellcome Trust grants 090909/Z to D.J.O., 097040 to A.G.W., and 100140 to CIMR.

Received: November 9, 2015
Revised: April 8, 2016
Accepted: May 2, 2016
Published: May 26, 2016

REFERENCES

Aguet, F., Antonescu, C.N., Mettlen, M., Schmid, S.L., and Danuser, G. (2013). Advances in analysis of low signal-to-noise images link dynamin and AP2 to the functions of an endocytic checkpoint. Dev. Cell *26*, 279–291.

Antonescu, C.N., Aguet, F., Danuser, G., and Schmid, S.L. (2011). Phosphatidylinositol-(4,5)-bisphosphate regulates clathrin-coated pit initiation, stabilization, and size. Mol. Biol. Cell *22*, 2588–2600.

Avinoam, O., Schorb, M., Beese, C.J., Briggs, J.A., and Kaksonen, M. (2015). Endocytic sites mature by continuous bending and remodeling of the clathrin coat. Science *348*, 1369–1372.

Benmerah, A., Begue, B., Dautry-Varsat, A., and Cerf-Bensussan, N. (1996). The ear of α-adaptin interacts with the COOH-terminal domain of the eps15 protein. J. Biol. Chem. *271*, 12111–12116.

Benmerah, A., Bayrou, M., Cerf-Bensussan, N., and Dautry-Varsat, A. (1999). Inhibition of clathrin-coated pit assembly by an Eps15 mutant. J. Cell Sci. *112*, 1303–1311.

Bitsikas, V., Correa, I.R., and Nichols, B.J. (2014). Clathrin-independent pathways do not contribute significantly to endocytic flux. Elife *3*, e03970.

Borner, G.H., Antrobus, R., Hirst, J., Bhumbra, G.S., Kozik, P., Jackson, L.P., Sahlender, D.A., and Robinson, M.S. (2012). Multivariate proteomic profiling identifies novel accessory proteins of coated vesicles. J. Cell Biol. *197*, 141–160.

Brett, T.J., Traub, L.M., and Fremont, D.H. (2002). Accessory protein recruitment motifs in clathrin-mediated endocytosis. Structure (Camb.) *10*, 797–809.

Carbone, R., Fre, S., Iannolo, G., Belleudi, F., Mancini, P., Pelicci, P.G., Torrisi, M.R., and Di Fiore, P.P. (1997). eps15 and eps15R are essential components of the endocytic pathway. Cancer Res. *57*, 5498–5504.

Cupers, P., ter Haar, E., Boll, W., and Kirchhausen, T. (1997). Parallel dimers and anti-parallel tetramers formed by epidermal growth factor receptor pathway substrate clone 15. J. Biol. Chem. *272*, 33430–33434.

Edeling, M.A., Mishra, S.K., Keyel, P.A., Steinhauser, A.L., Collins, B.M., Roth, R., Heuser, J.E., Owen, D.J., and Traub, L.M. (2006). Molecular switches involving the AP-2 β2 appendage regulate endocytic cargo selection and clathrin coat assembly. Dev. Cell *10*, 329–342.

Ehrlich, M., Boll, W., Van Oijen, A., Hariharan, R., Chandran, K., Nibert, M.L., and Kirchhausen, T. (2004). Endocytosis by random initiation and stabilization of clathrin-coated pits. Cell *118*, 591–605.

Gadeyne, A., Sanchez-Rodriguez, C., Vanneste, S., Di Rubbo, S., Zauber, H., Vanneste, K., Van Leene, J., De Winne, N., Eeckhout, D., Persiau, G., et al. (2014). The TPLATE adaptor complex drives clathrin-mediated endocytosis in plants. Cell *156*, 691–704.

Godlee, C., and Kaksonen, M. (2013). From uncertain beginnings: initiation mechanisms of clathrin-mediated endocytosis. J. Cell Biol. *203*, 717–725.

Hein, M.Y., Hubner, N.C., Poser, I., Cox, J., Nagaraj, N., Toyoda, Y., Gak, I., Weisswange, I., Mansfeld, J., Buchholz, F., et al. (2015). A human interactome in three quantitative dimensions organized by stoichiometries and abundances. Cell *163*, 712–723.

Henne, W.M., Kent, H.M., Ford, M.G., Hegde, B.G., Daumke, O., Butler, P.J., Mittal, R., Langen, R., Evans, P.R., and McMahon, H.T. (2007). Structure and analysis of FCHo2 F-BAR domain: a dimerizing and membrane recruitment module that effects membrane curvature. Structure *15*, 839–852.

Henne, W.M., Boucrot, E., Meinecke, M., Evergren, E., Vallis, Y., Mittal, R., and McMahon, H.T. (2010). FCHo proteins are nucleators of clathrin-mediated endocytosis. Science *328*, 1281–1284.

Hinrichsen, L., Harborth, J., Andrees, L., Weber, K., and Ungewickell, E.J. (2003). Effect of clathrin heavy chain- and α-adaptin specific small interfering RNAs on endocytic accessory proteins and receptor trafficking in HeLa cells. J. Biol. Chem. *278*, 45160–45170.

Hinrichsen, L., Meyerholz, A., Groos, S., and Ungewickell, E.J. (2006). Bending a membrane: how clathrin affects budding. Proc. Natl. Acad. Sci. USA *103*, 8715–8720.

Hirst, J., Schlacht, A., Norcott, J.P., Traynor, D., Bloomfield, G., Antrobus, R., Kay, R.R., Dacks, J.B., and Robinson, M.S. (2014). Characterization of TSET, an ancient and widespread membrane trafficking complex. Elife *3*, e02866.

Hollopeter, G., Lange, J.J., Zhang, Y., Vu, T.N., Gu, M., Ailion, M., Lambie, E.J., Slaughter, B.D., Unruh, J.R., Florens, L., et al. (2014). The membrane-associated proteins FCHo and SGIP are allosteric activators of the AP2 clathrin adaptor complex. Elife *3*, http://dx.doi.org/10.7554/eLife.03648.

Humphrey, J.S., Peters, P.J., Yuan, L.C., and Bonifacino, J.S. (1993). Localization of TGN38 to the trans-Golgi network: involvement of a cytoplasmic tyrosine-containing sequence. J. Cell Biol. *120*, 1123–1135.

Iannolo, G., Salcini, A.E., Gaidarov, I., Goodman, O.B., Jr., Baulida, J., Carpenter, G., Pelicci, P.G., Di Fiore, P.P., and Keen, J.H. (1997). Mapping of the molecular determinants involved in the interaction between eps15 and AP-2. Cancer Res. *57*, 240–245.

Jackson, L.P., Kelly, B.T., McCoy, A.J., Gaffry, T., James, L.C., Collins, B.M., Honing, S., Evans, P.R., and Owen, D.J. (2010). A large-scale conformational change couples membrane recruitment to cargo binding in the AP2 clathrin adaptor complex. Cell *141*, 1220–1229.

Katoh, M. (2004). Identification and characterization of human *FCHO2* and mouse *Fcho2* genes *in silico*. Int. J. Mol. Med. *14*, 327–331.

Kelly, B.T., Graham, S.C., Liska, N., Dannhauser, P.N., Höning, S., Ungewickell, E.J., and Owen, D.J. (2014). AP2 controls clathrin polymerization with a membrane-activated switch. Science *345*, 459–463.

Larson, B.T., Sochacki, K.A., Kindem, J.M., and Taraska, J.W. (2014). Systematic spatial mapping of proteins at exocytic and endocytic structures. Mol. Biol. Cell *25*, 2084–2093.

McMahon, H.T., and Boucrot, E. (2011). Molecular mechanism and physiological functions of clathrin-mediated endocytosis. Nat. Rev. Mol. Cell Biol. *12*, 517–533.

Meyerholz, A., Hinrichsen, L., Groos, S., Esk, P.C., Brandes, G., and Ungewickell, E.J. (2005). Effect of clathrin assembly lymphoid myeloid leukemia protein depletion on clathrin coat formation. Traffic *6*, 1225–1234.

Miller, S.E., Mathiasen, S., Bright, N.A., Pierre, F., Kelly, B.T., Kladt, N., Schauss, A., Merrifield, C.J., Stamou, D., Honing, S., et al. (2015). CALM regulates clathrin-coated vesicle size and maturation by directly sensing and driving membrane curvature. Dev. Cell *33*, 163–175.

Motley, A., Bright, N.A., Seaman, M.N., and Robinson, M.S. (2003). Clathrin-mediated endocytosis in AP-2-depleted cells. J. Cell Biol. *162*, 909–918.

Motley, A.M., Berg, N., Taylor, M.J., Sahlender, D.A., Hirst, J., Owen, D.J., and Robinson, M.S. (2006). Functional analysis of AP-2 α and μ2 subunits. Mol. Biol. Cell *17*, 5298–5308.

Mulkearns, E.E., and Cooper, J.A. (2012). FCHO2 organizes clathrin-coated structures and interacts with Dab2 for LDLR endocytosis. Mol. Biol. Cell *23*, 1330–1342.

Olesen, L.E., Ford, M.G., Schmid, E.M., Vallis, Y., Babu, M.M., Li, P.H., Mills, I.G., McMahon, H.T., and Praefcke, G.J. (2008). Solitary and repetitive binding motifs for the AP2 complex alpha-appendage in amphiphysin and other accessory proteins. J. Biol. Chem. *283*, 5099–5109.

Praefcke, G.J., Ford, M.G., Schmid, E.M., Olesen, L.E., Gallop, J.L., Peak-Chew, S.Y., Vallis, Y., Babu, M.M., Mills, I.G., and McMahon, H.T. (2004). Evolving nature of the AP2 α-appendage hub during clathrin-coated vesicle endocytosis. EMBO J. *23*, 4371–4383.

Reider, A., and Wendland, B. (2011). Endocytic adaptors - social networking at the plasma membrane. J. Cell Sci. *124*, 1613–1622.

Reider, A., Barker, S.L., Mishra, S.K., Im, Y.J., Maldonado-Baez, L., Hurley, J.H., Traub, L.M., and Wendland, B. (2009). Syp1 is a conserved endocytic adaptor that contains domains involved in cargo selection and membrane tubulation. EMBO J. *28*, 3103–3116.

Reis, C.R., Chen, P.H., Srinivasan, S., Aguet, F., Mettlen, M., and Schmid, S.L. (2015). Crosstalk between Akt/GSK3beta signaling and dynamin-1 regulates clathrin-mediated endocytosis. EMBO J. *34*, 2132–2146.

Robert, X., and Gouet, P. (2014). Deciphering key features in protein structures with the new ENDscript server. Nucleic Acids Res. *42*, W320–W324.

Robinson, M.S. (1993). Assembly and targeting of adaptin chimeras in transfected cells. J. Cell Biol. *123*, 67–77.

Schifferer, M., Feng, S., Stein, F., Tischer, C., and Schultz, C. (2015). Reversible chemical dimerizer-induced recovery of PIP levels moves clathrin to the plasma membrane. Bioorg. Med. Chem. *23*, 2862–2867.

Schindelin, J., Arganda-Carreras, I., Frise, E., Kaynig, V., Longair, M., Pietzsch, T., Preibisch, S., Rueden, C., Saalfeld, S., Schmid, B., et al. (2012). Fiji: an open-source platform for biological-image analysis. Nat. Methods *9*, 676–682.

Schmid, E.M., Ford, M.G., Burtey, A., Praefcke, G.J., Peak Chew, S.Y., Mills, I.G., Benmerah, A., and McMahon, H.T. (2006). Role of the AP2 β-appendage hub in recruiting partners for clathrin coated vesicle assembly. PLoS Biol. *4*, e262.

Suckling, R.J., Poon, P.P., Travis, S.M., Majoul, I.V., Hughson, F.M., Evans, P.R., Duden, R., and Owen, D.J. (2015). Structural basis for the binding of tryptophan-based motifs by δ-COP. Proc. Natl. Acad. Sci. USA *112*, 14242–14247.

Taylor, M.J., Perrais, D., and Merrifield, C.J. (2011). A high precision survey of the molecular dynamics of mammalian clathrin mediated endocytosis. PLoS Biol. *9*, e1000604.

Tebar, F., Sorkina, T., Sorkin, A., Ericsson, M., and Kirchhausen, T. (1996). eps15 is a component of clathrin-coated pits and vesicles and is located at the rim of clathrin-coated pits. J. Biol. Chem. *271*, 28727–28730.

Teckchandani, A., Mulkearns, E.E., Randolph, T.W., Toida, N., and Cooper, J.A. (2012). The clathrin adaptor Dab2 recruits EH domain scaffold proteins to regulate integrin β1 endocytosis. Mol. Biol. Cell *23*, 2905–2916.

Traub, L.M. (2009). Tickets to ride: selecting cargo for clathrin-regulated internalization. Nat. Rev. Mol. Cell Biol. *10*, 583–596.

Uezu, A., Horiuchi, A., Kanda, K., Kikuchi, N., Umeda, K., Tsujita, K., Suetsugu, S., Araki, N., Yamamoto, H., Takenawa, T., et al. (2007). SGIP1α is an endocytic protein that directly interacts with phospholipids and Eps15. J. Biol. Chem. *282*, 26481–26489.

Uezu, A., Umeda, K., Tsujita, K., Suetsugu, S., Takenawa, T., and Nakanishi, H. (2011). Characterization of the EFC/F-BAR domain protein, FCHO2. Genes Cells *16*, 868–878.

Umasankar, P.K., Sanker, S., Thieman, J.R., Chakraborty, S., Wendland, B., Tsang, M., and Traub, L.M. (2012). Distinct and separable activities of the endocytic clathrin-coat components Fcho1/2 and AP-2 in developmental patterning. Nat. Cell Biol. *14*, 488–501.

Umasankar, P.K., Ma, L., Thieman, J.R., Jha, A., Doray, B., Watkins, S.C., and Traub, L.M. (2014). A clathrin coat assembly role for the muniscin protein central linker revealed by TALEN-mediated gene editing. Elife *3*, e04137.

Wu, X., Zhao, X., Puertollano, R., Bonifacino, J.S., Eisenberg, E., and Greene, L.E. (2003). Adaptor and clathrin exchange at the plasma membrane and *trans*-Golgi network. Mol. Biol. Cell *14*, 516–528.

Zoncu, R., Perera, R.M., Sebastian, R., Nakatsu, F., Chen, H., Balla, T., Ayala, G., Toomre, D., and De Camilli, P.V. (2007). Loss of endocytic clathrin-coated pits upon acute depletion of phosphatidylinositol 4,5-bisphosphate. Proc. Natl. Acad. Sci. USA *104*, 3793–3798.

Developmental Cell, Volume *37*

Supplemental Information

Transient Fcho1/2·Eps15/R·AP-2 Nanoclusters

Prime the AP-2 Clathrin Adaptor for Cargo Binding

Li Ma, Perunthottathu K. Umasankar, Antoni G. Wrobel, Anastasia Lymar, Airlie J. McCoy, Sachin S. Holkar, Anupma Jha, Tirthadipa Pradhan-Sundd, Simon C. Watkins, David J. Owen, and Linton M. Traub

Inventory of Supplemental Materials:

Figure S1, related to Figure 1. The μHD protein–protein interaction web.

Figure S2, related to Figure 3. Fcho1 μHD co-crystal and evolutionary conservation of the concave μHD interaction surface upon subdomain A.

Figure S3, related to Figure 3. Additional characterization of structure-guided mutations in FCHO1 μHD subdomain A.

Figure S4, related to Figure 4. Tac-μHD lures only select endocytic pioneers/CLASPs onto intracellular compartments.

Figure S5, related to Figure 4 and 5. Remodeling of cell-surface AP-2 puncta in presence of ectopic Tac-Sgip1 μHD expression or after extinguishing EPS15 and EPS15R with transfected siRNA SMARTpools.

Figure S6, related to Figure 5. Defective CME in FCHO1/2-null HeLa 1.E cells expressing Tac-μHD.

Figure S7, related to Figure 6. Characterization of the HeLa SS6 β2-YFP clone #46β FCHO2-null line

Table S1, related to Figure 3. Data collection and refinement statistics.

Supplemental Experimental Procedures

Supplemental References

Figure S1

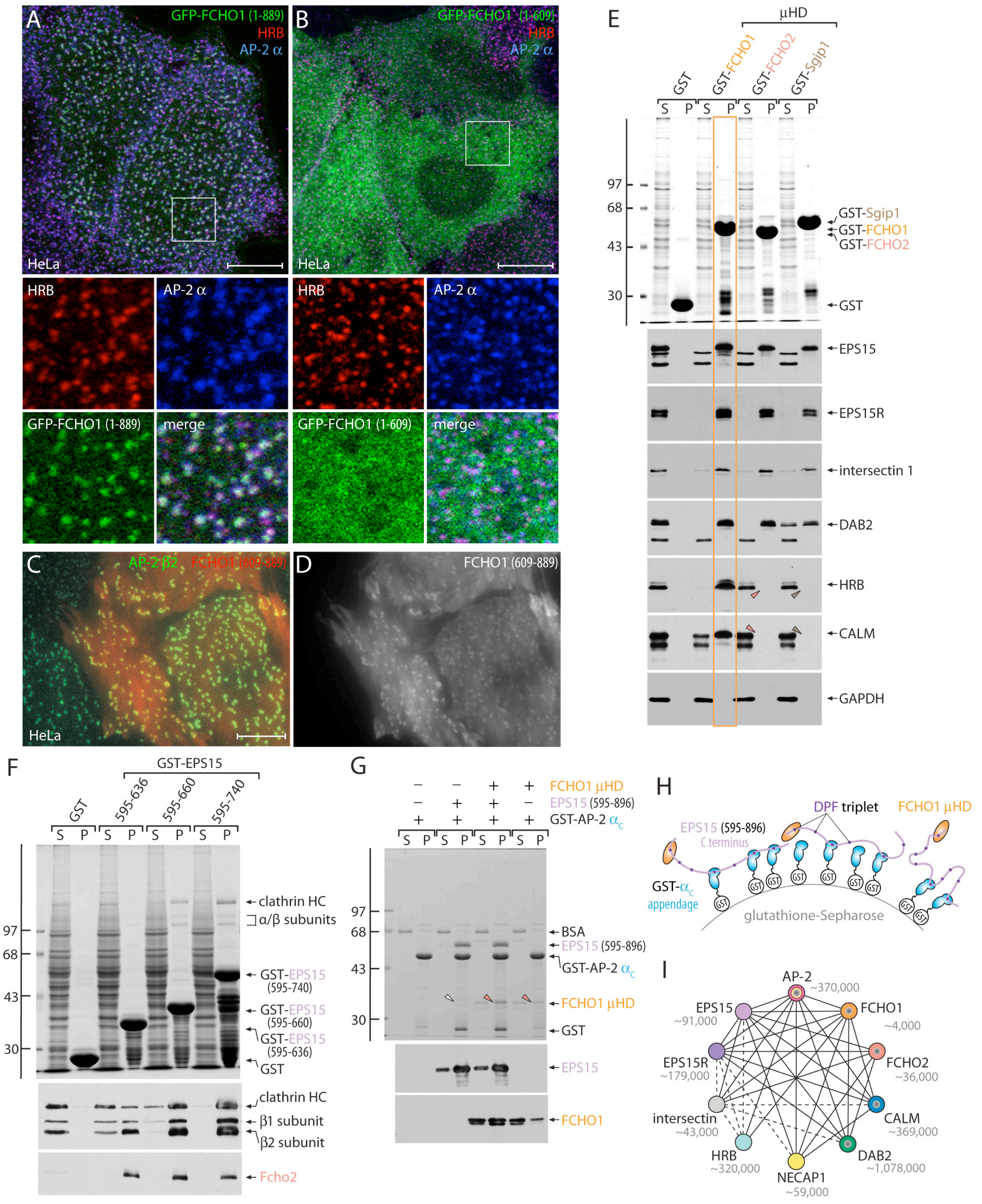
A
GFP-FCHO1 (1-889)
HRB
AP-2 α
HeLa
HRB
AP-2 α
GFP-FCHO1 (1-889)
merge
B
GFP-FCHO1 (1-609)
HRB
AP-2 α
HeLa
HRB
AP-2 α
GFP-FCHO1 (1-609)
merge
C
AP-2 β2
FCHO1 (609-889)
HeLa
D
FCHO1 (609-889)
E
µHD
GST
GST-FCHO1
GST-FCHO2
GST-Sgip1
S P S P S P S P
97
68
43
30
GST-Sgip1
GST-FCHO1
GST-FCHO2
GST
EPS15
EPS15R
intersectin 1
DAB2
HRB
CALM
GAPDH
F
GST-EPS15
GST
595-636
595-660
595-740
S P S P S P S P
97
68
43
30
clathrin HC
α/β subunits
GST-EPS15 (595-740)
GST-EPS15 (595-660)
GST-EPS15 (595-636)
GST
clathrin HC
β1 subunit
β2 subunit
Fcho2
G
FCHO1 µHD
EPS15 (595-896)
GST-AP-2 αc
S P S P S P S P
97
68
43
30
BSA
EPS15 (595-896)
GST-AP-2 αc
FCHO1 µHD
GST
EPS15
FCHO1
H
DPF triplet
FCHO1 µHD
EPS15 (595-896)
C terminus
GST-αc
appendage
glutathione-Sepharose
I
AP-2
~370,000
EPS15
~91,000
FCHO1
~4,000
EPS15R
~179,000
FCHO2
~36,000
intersectin
~43,000
CALM
~369,000
HRB
~320,000
DAB2
~1,078,000
NECAP1
~59,000

Figure S1, linked to Figure 1. The μHD protein–protein interaction web

A-B. Confocal analysis of HeLa SS6 cells transfected with GFP-FCHO1 (1-889) (A) or GFP-FCHO1 (1-609) lacking the μHD (B). Fixed cells were probed with indicated antibodies against the AP-2 α subunit and HRB. Representative merged single confocal sections and enlarged color-separated regions corresponding to the boxed areas are shown. Scale bar = 10 μm.

C-D. Two-color total internal reflection fluorescence microscopy (TIRFM) of stable AP-2 β2-YFP expressing HeLa SS6 cells transfected with tdRFP-FCHO1 (609-889; μHD). A representative merged (C) and a μHD (red channel) image (D) is shown. Scale bar = 10 μm.

E. GST pull-down assays with HeLa cell lysate and 250 μg of either GST or the indicated GST-μHD fusion proteins. Aliquots of 2% of each supernantant (S) and 10% of each pellet (P) fraction were resolved by SDS-PAGE and either stained with Coomassie blue or transferred to nitrocellulose. Replicate blots were probed with the indicated antibodies. Pellet fraction showing solid phase interactions with the FCHO1 μHD is boxed (orange), while arrowheads indicate the lack of a similar interaction with the FCHO2 or Sgip1 μHD. Note that the large splice isoform of CALM binds preferentially to the FCHO1 μHD; this protein differs from the smaller isoform(s) by alternative splicing of a 50 amino-acid residue-encoding exon that contains a DPF tripeptide, supporting the direct role of DPF recognition in μHD associations.

F.GST pull-down assay with rat brain cytosol and ~250 μg of either GST or GST-EPS15 (595-636), (595-660) or (595-740) proteins immobilized on glutathione-Sepharose. Aliquots of each supernatant (2%) and pellet (10%) fraction were resolved by SDS-PAGE and either stained with Coomassie blue or transferred to nitrocellulose. Replicate blots were probed with a mixture of antibodies directed against the clathrin heavy chain (HC) and the β1/β2 subunit of AP-1/2 or an antibody against Fcho2 as indicated.

G. Two-stage pull-down assay with immobilized GST-α_C appendage incubated first with EPS15 (595-896) followed by addition of the FCHO1 μHD in presence of carrier BSA. A stained gel and corresponding blots probed with the indicated antibodies against the EPS15 C terminus and the FCHO1 μHD are shown. The migratory position of the monomeric μHD is indicated (arrowheads).

H. Schematic representation of the conceptual interpretation of the results in G.

I. Wiring diagram of the clathrin pioneer protein biophysical network. Edges show known direct interactions, broken lines denote probable direct physical interactions, and gray centers connote direct PtdIns(4,5)P_2 binding. Approximate copy number estimates in HeLa cells (Hein et al., 2015) are shown.

Figure S2

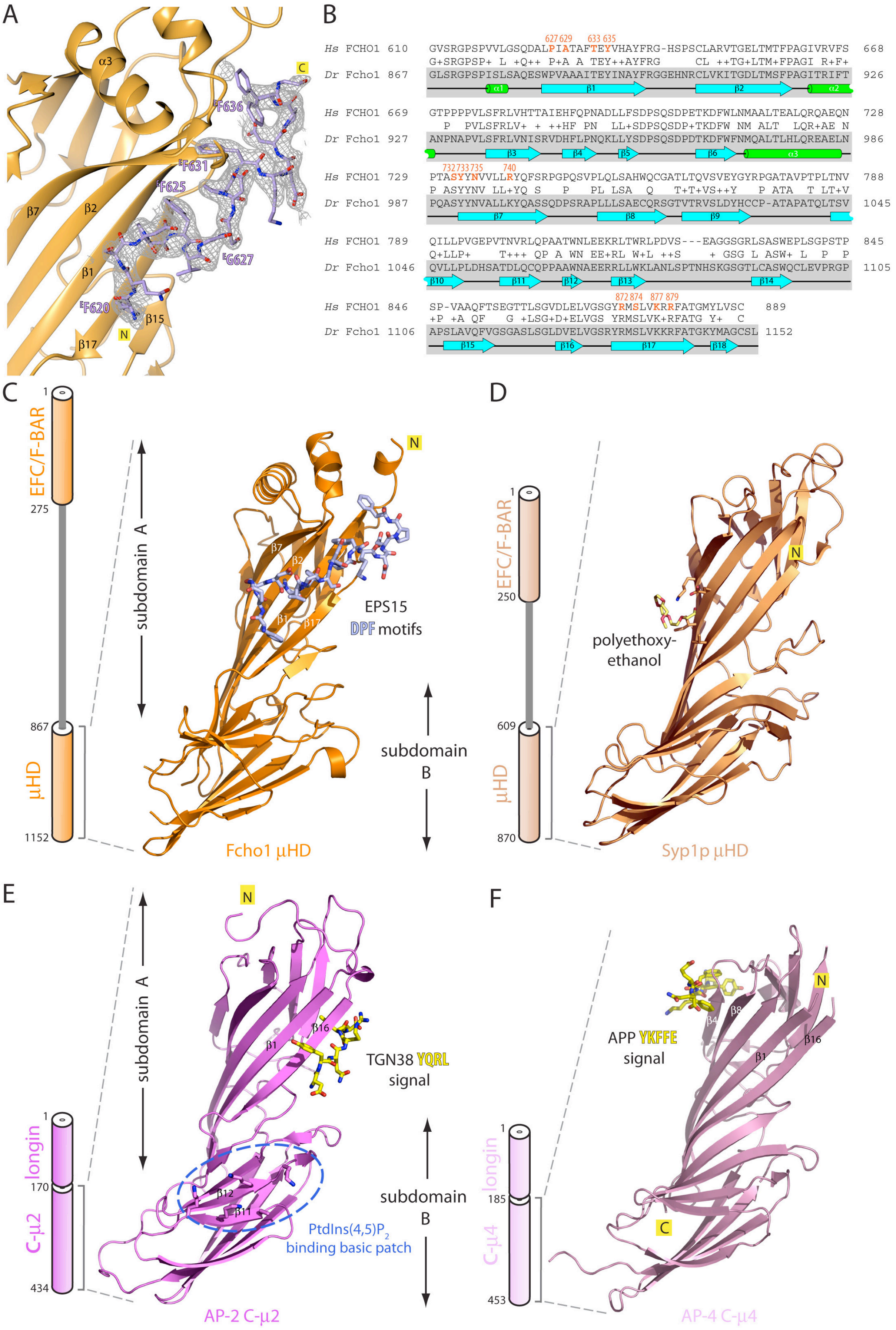
A
α3
C
EF636
EF631
EF625
β7
β2
β1
EG627
EF620
β15
β17
N
B
627 629
633 635
Hs FCHO1 610 GVSRGPSPVVLGSQDALPIATAFTEYVHAYFRG-HSPSCLARVTGELTMTFPAGIVRVFS 668
G+SRGPSP+ L +Q++ P+A A TEY++AYFRG CL ++TG+LTM+FPAGI R+F+
Dr Fcho1 867 GLSRGPSPISLSAQESWPVAAAITEYINAYFRGGEHNRCLVKITGDLTMSFPAGITRIFT 926
α1 β1 β2 α2
Hs FCHO1 669 GTPPPPPVLSFRLVHTTAIEHFQPNADLLFSDPSQSDPETKDFWLNMAALTEALQRQAEQN 728
P PVLSFRLV+ + ++HF PN LL+SDPSQSDP+TKDFW NM ALT LQR+AE N
Dr Fcho1 927 ANPNAPVLSFRLVNISRVDHFLPNQKLLYSDPSQSDPDTKDFWFNMQALTLHLQREAELN 986
β3 β4 β5 β6 α3
732 733 735 740
Hs FCHO1 729 PTASYYNVVLLRYQFSRPGPQSVPLQLSAHWQCGATLTQVSVEYGYRPGATAVPTPLTNV 788
P ASYYNV LL+YQ S P PL LSA Q T+T+VS++Y P ATA T LT+V
Dr Fcho1 987 PQASYYNVALLKYQASSQDPSRAPLLLSAECQRSGTVTRVSLDYHCCP-ATAPATQLTSV 1045
β7 β8 β9
Hs FCHO1 789 QILLPVGEPVTNVRLQPAATWNLEEKRLTWRLPDVS---EAGGSGRLSASWEPLSGPSTP 845
Q+LLP+ T+++ QP A WN EE+RL W+L ++S + GSG L ASW+ L P P
Dr Fcho1 1046 QVLLPLDHSATDLQCQPPAAWNAEERRLLWKLANLSPTNHSKGSGTLCASWQCLEVPRGP 1105
β10 β11 β12 β13 β14
872 874 877 879
Hs FCHO1 846 SP-VAAQFTSEGTTLSGVDLELVGSGYRMSLVKRRFATGMYLVSC 889
+P +A QF G +LSG+D+ELVGS YRMSLVK+RFATG Y+ C
Dr Fcho1 1106 APSLAVQFVGSGASLSGLDVELVGSRYRMSLVKKRFATGKYMAGCSL 1152
β15 β16 β17 β18
C
1
EFC/F-BAR
275
867
μHD
1152
subdomain A
N
β7
β2
β1
β17
EPS15
DPF motifs
subdomain B
Fcho1 μHD
D
1
EFC/F-BAR
250
609
μHD
870
N
polyethoxy-
ethanol
Syp1p μHD
E
1
longin
170
C-μ2
434
subdomain A
N
β1
β16
TGN38 YQRL
signal
β12
β11
PtdIns(4,5)P2
binding basic patch
subdomain B
AP-2 C-μ2
F
1
longin
185
C-μ4
453
N
APP YKFFE
signal
β4
β8
β1
β16
C
AP-4 C-μ4

Figure S2, linked to Figure 3. Fcho1 μHD co-crystal and evolutionary conservation of the concave μHD interaction surface upon subdomain A

A. Electron density map of the EPS15 peptide bound to the zebrafish (*Danio rerio*) Fcho1 μHD. The final refined 2Fo-Fc electron density map contoured at 0.76σ is shown. The interacting EPS15 peptide (mauve) shown in stick representation is superimposed, with nitrogen colored blue and oxygen atoms red. Adjacent is a ribbon representation of a zoomed-in region of subdomain A (orange) from the co-crystal that defines the interaction interface.

B. Primary sequence alignment of the *H. sapiens* (*Hs*) and *D. rerio* (*Dr*) Fcho1 μHDs relative to the secondary structural elements (α-helix, green; β-strand, cyan) present in the *Dr* μHD structure (boxed in gray). The location of the corresponding residues mutated and tested in the *Hs* FCHO1 μHD are shown in orange type.

C. Schematic depiction of the overall domain organization of zebrafish Fcho1 (isoform X1, XM_009303910) alongside a ribbon representation of the backbone of entire Fcho1 μHD. The EPS15 peptide is colored as in A.

D. Domain organization and μHD (PDB ID 3G9H) ribbon diagram for *Saccharomuces cerevisiae* Syp1p. The location of a polyethoxyethanol additive that co-crystallizes with the Syp1p μHD is indicated in yellow sticks (Reider et al., 2009).

E. AP-2 μ2 subunit arrangement and C-μ2 structure (PDB ID 1BXX) ribbon diagram with the bound DYQRLN peptide from TGN38 shown in yellow stick representation (Owen and Evans, 1998). The location of the subdomain B patch with five basic side chains that coordinate a phosphatidylinositol 4,5-bisphosphate (PtdIns(4,5)P_2) head group is shown (blue broken oval).

F. AP-4 μ4 domain organization and C-μ4 (PDB ID 3L81) ribbon diagram, with bound YKFFE peptide from amyloid precursor protein shown in yellow stick representation (Burgos et al., 2010).

Figure S3

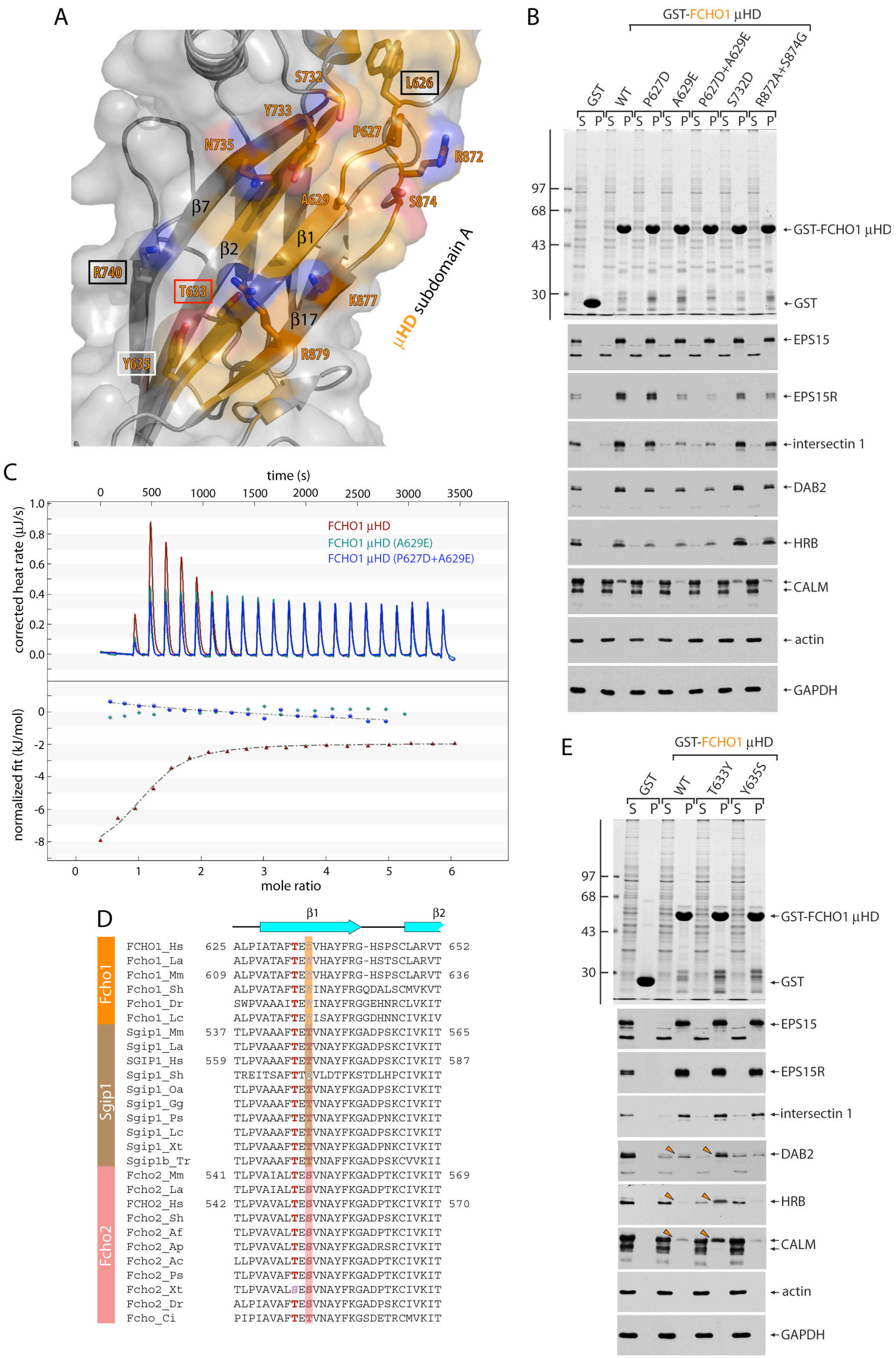
A
S732
L626
Y733
P627
N735
R872
A629
S874
β7
β1
β2
R740
T633
K877
β17
µHD subdomain A
Y635
R879
B
GST-FCHO1 µHD
GST
WT
P627D
A629E
P627D+A629E
S732D
R872A+S874G
S P
97
68
43
30
GST-FCHO1 µHD
GST
EPS15
EPS15R
intersectin 1
DAB2
HRB
CALM
actin
GAPDH
C
time (s)
0 500 1000 1500 2000 2500 3000 3500
corrected heat rate (µJ/s)
1.0 0.8 0.6 0.4 0.2 0.0
FCHO1 µHD
FCHO1 µHD (A629E)
FCHO1 µHD (P627D+A629E)
normalized fit (kJ/mol)
0 -2 -4 -6 -8
0 1 2 3 4 5 6
mole ratio
D
β1
β2
Fcho1
Sgip1
Fcho2
FCHO1_Hs 625 ALPIATAFTEYVHAYFRG-HSPSCLARVT 652
Fcho1_La ALPVATAFTEYVHAYFRG-HSTSCLARVT
Fcho1_Mm 609 ALPVATAFTEYVHAYFRG-HSPSCLARVT 636
Fcho1_Sh ALPVATAFTEYINAYFRGQDALSCMVKVT
Fcho1_Dr SWPVAAAITEYINAYFRGGEHNRCLVKIT
Fcho1_Lc ALPVATAFTEYISAYFRGGDHNNCIVKIV
Sgip1_Mm 537 TLPVAAAFTETVNAYFKGADPSKCIVKIT 565
Sgip1_La TLPVAAAFTETVNAYFKGADPSKCIVKIT
SGIP1_Hs 559 TLPVAAAFTETVNAYFKGADPSKCIVKIT 587
Sgip1_Sh TREITSAFTTCVLDTFKSTDLHPCIVKIT
Sgip1_Oa TLPVAAAFTETVNAYFKGADPSKCIVKIT
Sgip1_Gg TLPVAAAFTETVNAYFKGADPSKCIVKIT
Sgip1_Ps TLPVAAAFTETVNAYFKGADPNKCIVKIT
Sgip1_Lc TLPVAAAFTETVNAYFKGADPSKCIVKIT
Sgip1_Xt TLPVAAAFTETVNAYFKGADPNKCIVKIT
Sgip1b_Tr TLPVAAAFTETVNAFFKGADPSKCVVKII
Fcho2_Mm 541 TLPVAIALTESVNAYFKGADPTKCIVKIT 569
Fcho2_La TLPVAIALTESVNAYFKGADPTKCIVKIT
FCHO2_Hs 542 TLPVAVALTESVNAYFKGADPTKCIVKIT 570
Fcho2_Sh TLPVAVALTESVNAYFKGADPSKCIVKIT
Fcho2_Af TLPVAVALTESVNAYFKGADPTKCIVKIT
Fcho2_Ap TLPVAVALTESVNAYFKGADRSRCIVKIT
Fcho2_Ac LLPVAVALTESVNAYFKGADPTKCIVKIT
Fcho2_Ps TLPVAVAFTESVNAYFKGADPTKCIVKIT
Fcho2_Xt TLPVAVALSESVNAYFRGADPTKCIVKIT
Fcho2_Dr ALPIAVAFTESVNAYFKGADPSKCIVKIT
Fcho_Ci PIPIAVAFTETVNAYFKGSDETRCMVKIT
E
GST-FCHO1 µHD
GST
WT
T633Y
Y635S
S P
97
68
43
30
GST-FCHO1 µHD
GST
EPS15
EPS15R
intersectin 1
DAB2
HRB
CALM
actin
GAPDH

Figure S3, linked to Figure 3. Additional characterization of structure-guided mutations in FCHO1 μHD subdomain A

A. Combined ribbon and molecular surface representation of the *D. rerio* Fcho1 μHD with numbering of the corresponding *H. sapiens* FCHO1 μHD residues shown. Differing side chains are boxed in black, while the TXY residues in strand β1 at the base of the binding trough are boxed either in red (conserved in muniscins) or white (not conserved in Fcho2 or Sgip1), respectively. The location of side chains examined by mutagenesis is shown in stick representation with nitrogen colored blue and oxygen red.

B. GST pull-down assays with HeLa cell lysate and 125 μg of GST or various GST-FCHO1 μHD fusions as indicated. Separated supernatant (2%) and pellet (10%) fractions were either stained or replicates immunoblotted with the indicated antibodies. Notice that for this set of mutations, EPS15, EPS15R, intersectin 1, DAB2 and HRB are not recovered in the supernatant fractions, as is the case with the K877E+R879A mutation (Figure 3E-F). This indicates that these mutations to the helical region of subdomain A primarily affect the off-rate of the interactions with the μHD.

C. Representative ITC experiments with the wild-type *Hs* FCHO1 μHD or (A629E)- or (P627D+A629E)-substituted FCHO1 μHDs titrated with the EPS15 616-638 peptide. Titrations were performed at 4°C due to thermal instability and aggregation of these mutants upon prolonged stirring at 12°C.

D. Alignment of stand β1 amino acid regions from muniscin family members of selected organisms (genus/species abbreviations: Hs, *Homo sapiens*; La, *Loxodonta africana* (African bush elephant); Mm, *Mus musculus*; Sh; *Sarcophilus harrisii* (Tasmanian devil); Dr, *Danio rerio*; Lc, *Latimeria chalumnae* (coelacanth); Oa, *Ornithorhynchus anatinus* (duck-billed platypus); Gg, *Gallus gallus*; Ps, *Pelodiscus sinensis* (Chinese softshell turtle); Xt, *Xenopus tropicalis*; Tr, *Takifugu rubripes* (Japanese pufferfish); Af, *Aptenodytes forsteri* (emperor penguin); Ap, *Anas platyrhynchos* (domestic duck); Ac, *Anolis carolinensis* (green anole lizard); Ci, *Ciona intestinalis* (sea squirt)) are shown with appropriate residues numbers indicated. Identical residues are highlighted in red, highly similar residues in pale pink.

E. Pull-down assay with HeLa cell lysate and the indicated amount of GST, GST-FCHO1 μHD mutants immobilized on glutathione-Sepharose. Separated supernatant (2%) and pellet (10%) fractions were either stained or replicates immunoblotted with the indicated antibodies. The apparent gain-of-function effect of the T633Y substitution on the weaker binding partners is highlighted in comparison with the wild-type FCHO1 μHD (arrowheads).

Figure S4

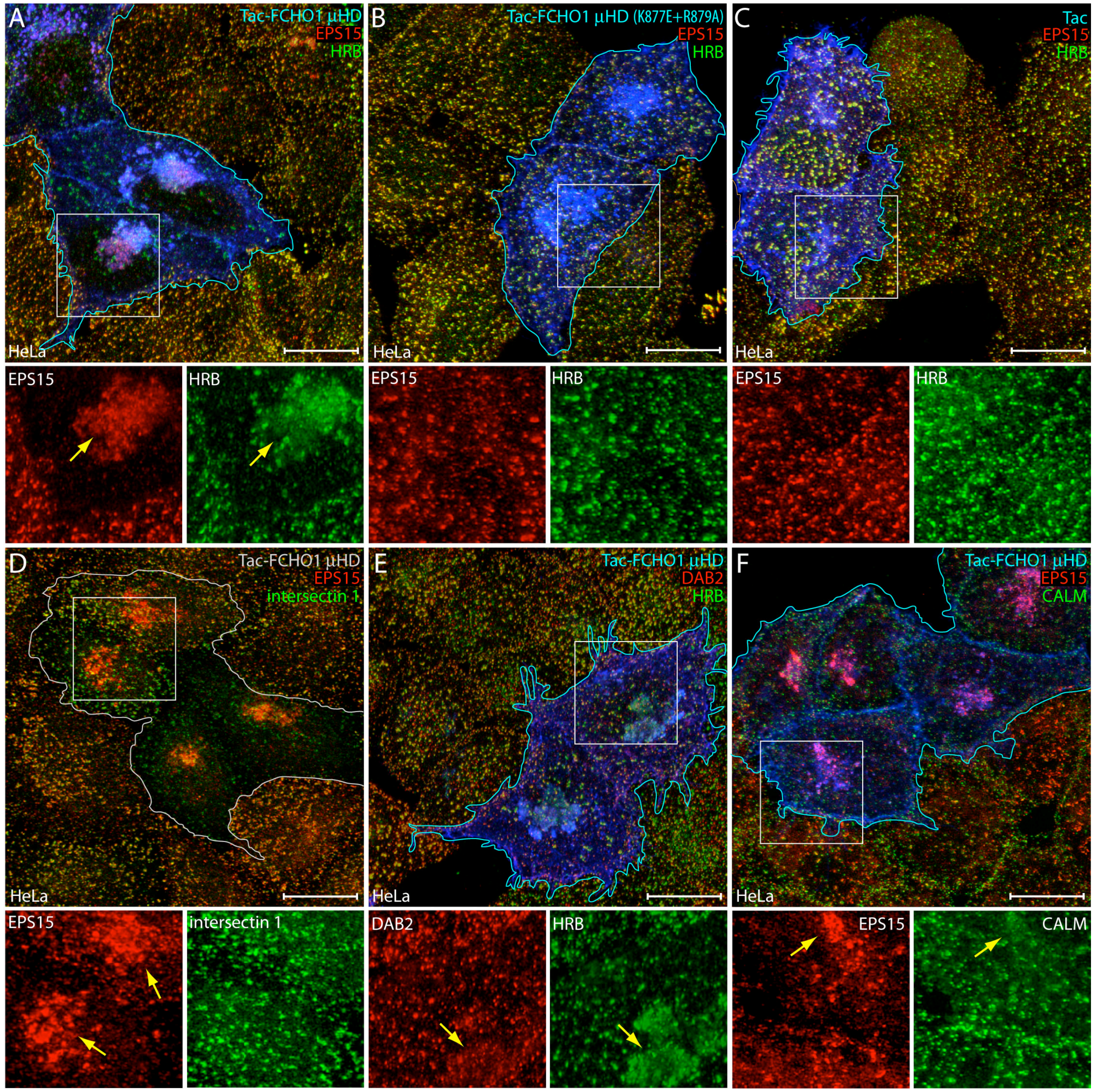
A
Tac-FCHO1 µHD
EPS15
HRB
HeLa
EPS15
HRB
B
Tac-FCHO1 µHD (K877E+R879A)
EPS15
HRB
HeLa
EPS15
HRB
C
Tac
EPS15
HRB
HeLa
EPS15
HRB
D
Tac-FCHO1 µHD
EPS15
intersectin 1
HeLa
EPS15
intersectin 1
E
Tac-FCHO1 µHD
DAB2
HRB
HeLa
DAB2
HRB
F
Tac-FCHO1 µHD
EPS15
CALM
HeLa
EPS15
CALM

Figure S4, linked to Figure 4. Tac-μHD lures only select endocytic pioneers/CLASPs onto intracellular compartments

A-C. Typical maximal intensity projections of confocal image *z* stacks from fixed HeLa cells transiently expressing Tac (E) or Tac-μHD (K877E+R819A) (F) and stained with antibodies against EPS15, HRB and Tac (transfected cell groups outlined in blue) as indicated. Enlarged, color-separated channels of the boxed regions are shown below. Unlike the Tac-μHD, little intracellular relocation of EPS15 or HRB (arrows) is apparent on overexpression of either Tac or the Tac-μHD (K877E+R879A) mutant.

D-F. Representative maximal intensity projections of deconvolved confocal image *z* stacks of HeLa SS6 cells transiently transfected with Tac-FCHO1 μHD. Fixed cells were stained with anti-EPS15, anti-intersectin 1, anti-Dab2, anti-HRB, anti-CALM and anti-Tac antibodies as indicated, Pronounced movement of EPS15 away from the surface (arrows) on expression of the wild-type μHD fused to the Tac C-terminus is not accompanied by equivalent repositioning of all μHD partners. Note that intersectin 1 resists prominent relocation onto intracellular membranes in parallel to EPS15, which identifies the Tac-μHD-transfected cells (gray outline) in absence of anti-Tac staining. Sale bar = 10 mm.

Figure S5

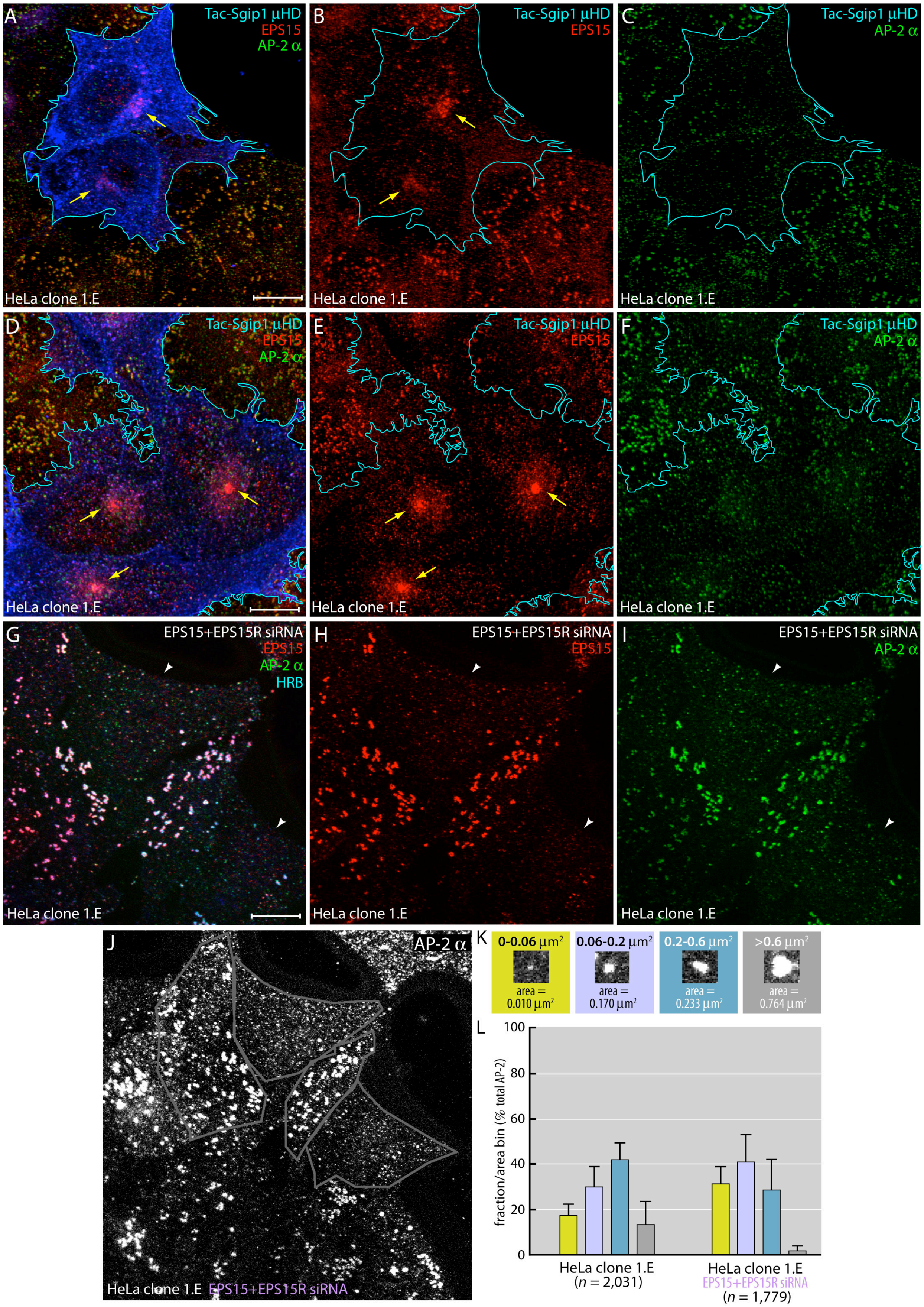

A
Tac-Sgip1 µHD
EPS15
AP-2 α
HeLa clone 1.E
B
Tac-Sgip1 µHD
EPS15
HeLa clone 1.E
C
Tac-Sgip1 µHD
AP-2 α
HeLa clone 1.E
D
Tac-Sgip1 µHD
EPS15
AP-2 α
HeLa clone 1.E
E
Tac-Sgip1 µHD
EPS15
HeLa clone 1.E
F
Tac-Sgip1 µHD
AP-2 α
HeLa clone 1.E
G
EPS15+EPS15R siRNA
EPS15
AP-2 α
HRB
HeLa clone 1.E
H
EPS15+EPS15R siRNA
EPS15
HeLa clone 1.E
I
EPS15+EPS15R siRNA
AP-2 α
HeLa clone 1.E
J
AP-2 α
HeLa clone 1.E EPS15+EPS15R siRNA
K
0-0.06 µm²
area = 0.010 µm²
0.06-0.2 µm²
area = 0.170 µm²
0.2-0.6 µm²
area = 0.233 µm²
>0.6 µm²
area = 0.764 µm²
L
fraction/area bin (% total AP-2)
100
80
60
40
20
0
HeLa clone 1.E
(n = 2,031)
HeLa clone 1.E
EPS15+EPS15R siRNA
(n = 1,779)

Figure S5, linked to Figures 4 and 5. Remodeling of cell-surface AP-2 puncta in presence of ectopic Tac-Sgip1 μHD expression or after extinguishing EPS15 and EPS15R with transfected siRNA SMARTpools

A-F. Two selected but representative maximal-projection images of deconvoluted confocal *z* stacks from HeLa clone 1.E cells transiently transfected a Tac-Sgip1 μHD plasmid. Fixed and permeabilized cells were stained with antibodies against EPS15, the α subunit of AP-2 and Tac (transfected cell groups outlined in blue), as indicated. Separated color-channels (B, C and E, F) are shown to highlight the change in AP-2 puncta when EPS15 is redirected to the cell interior with the Tac-Sgip1 μHD chimera. Scale bar = 10 μm.

G-I. HeLa clone 1.E cells were transfected twice sequentially with SMARTpools of siRNA oligonucleotides targeting both the *EPS15* and *EPS15L1* (EPS15R) transcripts. Fixed and permeabilized cells were labeled with antibodies raised against EPS15, the α subunit of AP-2 and HRB. A maximal projection of deconvolved *z* stacks is shown, with cells displaying minimal EPS15 signal and a corresponding alteration in the AP-2 distribution indicated with arrowheads. The changed AP-2 morphology is consistent with the phenotype observed with Tac-μHD expression in these cells. Note that in these experiments, we unfortunately cannot determine the expression level of EPS15R in individual cells due to a lack of a suitable antibody for immunofluorescence analysis. Scale bar = 10 μm.

J-L. Quantitative analysis of area distributions of AP-2 assemblies in EPS15 + EPS15R-silenced HeLa clone 1.E cells. A representative gray scale image of AP-2 α-subunit staining (J), examples of AP-2-positive puncta within the color-coded area bins selected (K), and puncta area distributions and standard deviations in EPS15 positive (clone 1.E) and siRNA-silenced EPS15-deficient cells (L) are shown.

Figure S6

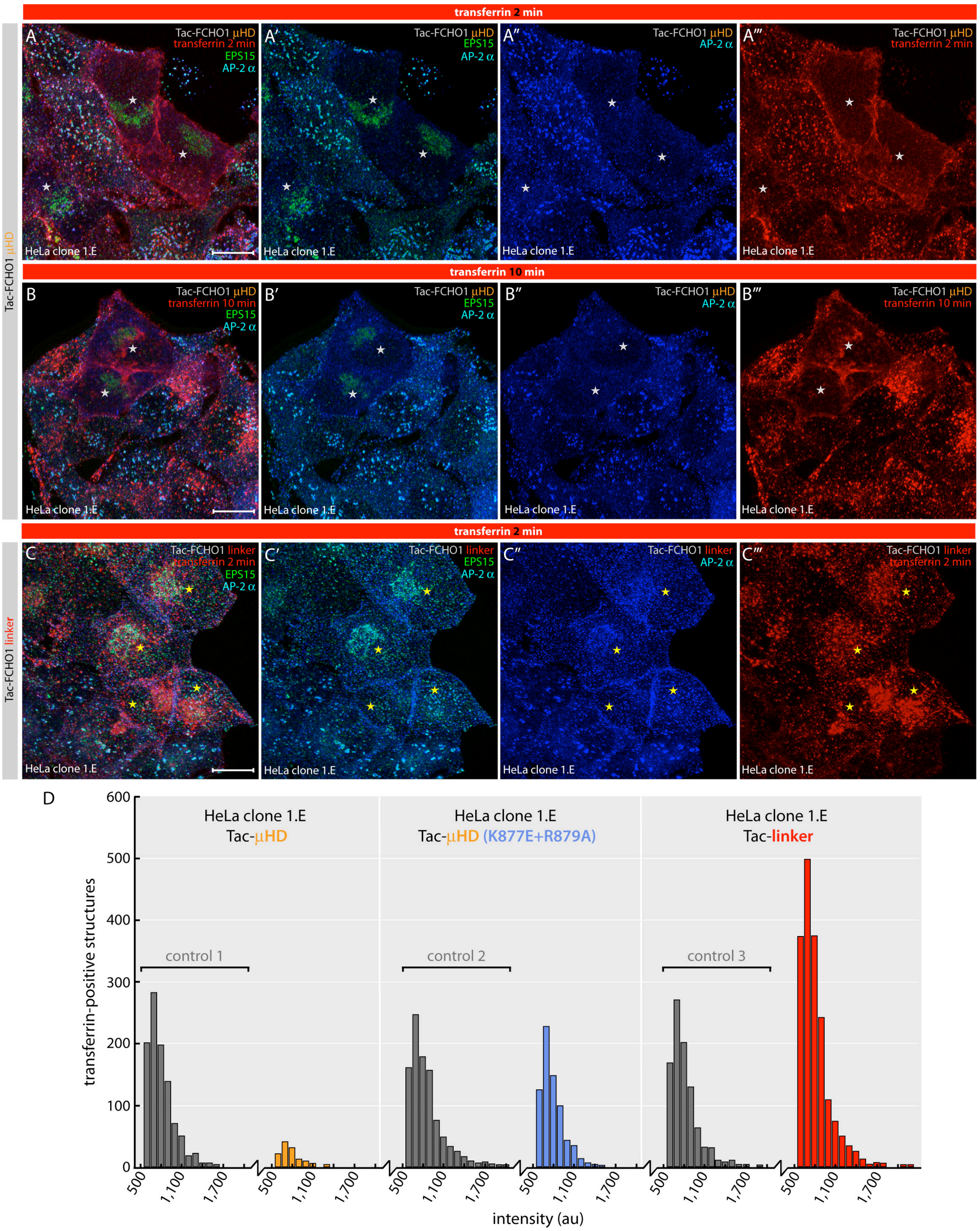
transferrin 2 min
Tac-FCHO1 µHD
A
Tac-FCHO1 µHD
transferrin 2 min
EPS15
AP-2 α
HeLa clone 1.E
A′
Tac-FCHO1 µHD
EPS15
AP-2 α
HeLa clone 1.E
A″
Tac-FCHO1 µHD
AP-2 α
HeLa clone 1.E
A‴
Tac-FCHO1 µHD
transferrin 2 min
HeLa clone 1.E
transferrin 10 min
B
Tac-FCHO1 µHD
transferrin 10 min
EPS15
AP-2 α
HeLa clone 1.E
B′
Tac-FCHO1 µHD
EPS15
AP-2 α
HeLa clone 1.E
B″
Tac-FCHO1 µHD
AP-2 α
HeLa clone 1.E
B‴
Tac-FCHO1 µHD
transferrin 10 min
HeLa clone 1.E
transferrin 2 min
Tac-FCHO1 linker
C
Tac-FCHO1 linker
transferrin 2 min
EPS15
AP-2 α
HeLa clone 1.E
C′
Tac-FCHO1 linker
EPS15
AP-2 α
HeLa clone 1.E
C″
Tac-FCHO1 linker
AP-2 α
HeLa clone 1.E
C‴
Tac-FCHO1 linker
transferrin 2 min
HeLa clone 1.E
D
HeLa clone 1.E
Tac-µHD
HeLa clone 1.E
Tac-µHD (K877E+R879A)
HeLa clone 1.E
Tac-linker
control 1
control 2
control 3
transferrin-positive structures
0
100
200
300
400
500
600
500
1,100
1,700
intensity (au)

Figure S6, linked to Figure 5. Defective CME in FCHO1/2-null HeLa 1.E cells expressing Tac-μHD

A-C. Selected but representative maximal intensity projection images of deconvoluted confocal *z* stacks from HeLa clone 1.E cells transiently transfected with either the Tac-FCHO1 μHD (A-B''') or the Tac-FCHO1 linker (C-C''') plasmid. Serum-starved cells were pulsed with Alexa Fluor 546 transferrin for 2 (A and C) or 10 (B) min before fixation. Permeabilized cells were stained with antibodies directed against EPS15 and the AP-2 α subunit as indicated. Composite images (A-C), dual color EPS15 and AP-2 α subunit (A'-C') as well as individual AP-2 α subunit (A''-C'') or transferrin (A'''-C''') color channels are shown. Tac-μHD-expressing cells are identified by prominent accumulation of EPS15 in the Golgi region (white asterisks) and by loss of the phenotypical enlarged CCSs on the ventral plasma membrane surface. These attributes correlate with the changed AP-2 distribution in the cells. By contrast, Tac-FCHO1 linker expressing 1.E cells have a different alteration to the steady-state distribution of AP-2, which is correlated with increased transferrin internalization (yellow asterisks). Scale bar = 10 μm.

D. Histograms of quantified data from transferrin pulse-chase uptake experiments performed as described in Figure 5. The total number of transferrin-positive internalized puncta (after a two minute chase at 37°C) counted from three or four separate confocal data sets are: HeLa clone 1.E cell control 1 = 980, Tac-μHD-expressing cells = 113; HeLa clone 1.E cell control 2 = 953, Tac-μHD (K877E+R879A)-expressing cells = 690; HeLa clone 1.E cell control 3 = 914, Tac-linker-expressing cells = 1,785, and the same aggregate total area (2,443.4 μm^2) was analyzed for each population.

Figure S7

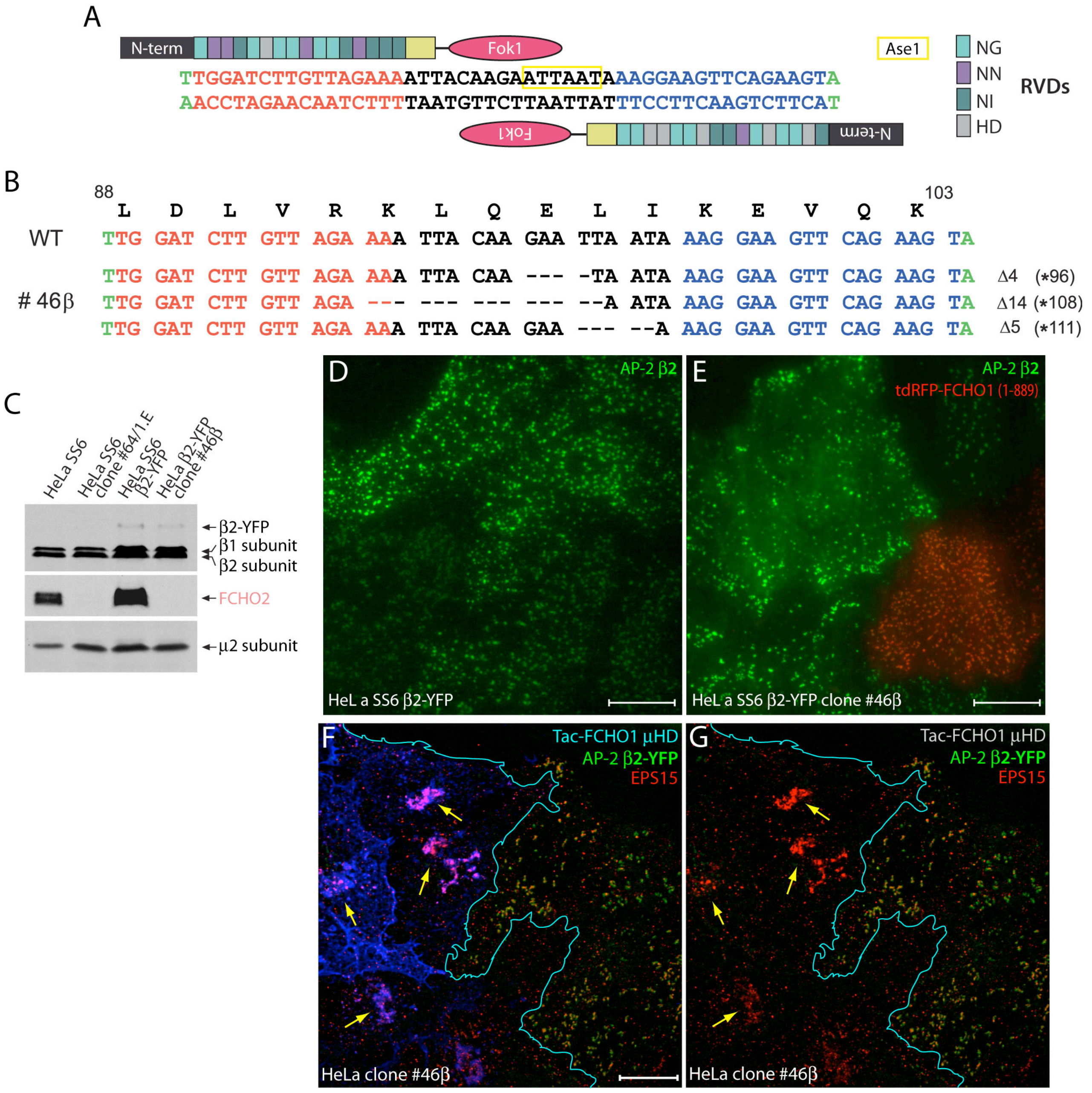
A
N-term
Fok1
Ase1
TTGGATCTTGTTAGAAAATTACAAGAATTAATAAAGGAAGTTCAGAAGTA
AACCTAGAACAATCTTTTAATGTTCTTAATTATTTCCTTCAAGTCTTCAT
Fok1
N-term
NG
NN
NI
HD
RVDs
B
88
L D L V R K L Q E L I K E V Q K
103
WT
TTG GAT CTT GTT AGA AAA TTA CAA GAA TTA ATA AAG GAA GTT CAG AAG TA
46β
TTG GAT CTT GTT AGA AAA TTA CAA --- -TA ATA AAG GAA GTT CAG AAG TA Δ4 (*96)
TTG GAT CTT GTT AGA --- --- --- --- --A ATA AAG GAA GTT CAG AAG TA Δ14 (*108)
TTG GAT CTT GTT AGA AAA TTA CAA GAA --- --A AAG GAA GTT CAG AAG TA Δ5 (*111)
C
HeLa SS6
HeLa SS6 clone #64/1.E
HeLa SS6 β2-YFP
HeLa β2-YFP clone #46β
β2-YFP
β1 subunit
β2 subunit
FCHO2
μ2 subunit
D
AP-2 β2
HeL a SS6 β2-YFP
E
AP-2 β2
tdRFP-FCHO1 (1-889)
HeL a SS6 β2-YFP clone #46β
F
Tac-FCHO1 μHD
AP-2 β2-YFP
EPS15
HeLa clone #46β
G
Tac-FCHO1 μHD
AP-2 β2-YFP
EPS15
HeLa clone #46β

Figure S7, linked to Figure 6. Characterization of the HeLa SS6 β2-YFP clone #46β FCHO2-null line

A. Design strategy for an exon 4-targeting TALEN pair mediated disruption of the *FCHO2* locus on chromosome 5 (Umasankar et al., 2014). The different repeat variable dipeptides (RVD) are color coded as indicated. The endogenous internal AseI restriction site used for screening is boxed (yellow).

B. Genotype of clone #46β cells compared with the wild-type (WT) sequence and encoded amino acid sequence above. The * symbol represents an in-frame stop codon at the indicated residue position. Unlike the parental HeLa SS6 cells (Umasankar et al., 2014), the β2-YFP stable line derived therefrom is clearly aneuploid, with three copies of chromosome 5. Two other independent clones (#5β and #52β) similarly have three (differently) disrupted alleles of the *FCHO2* locus.

C. Immunoblot comparison of HeLa SS6 parental cells, the clone#64/1.E FCHO1+FCHO2-null cells, the HeLa SS6 β2-YFP stable cell line, and the clone #46β FCHO2 null β2-YFP expressing line. Note β2-YFP expression levels in these cells are substoichiometric with the endogenous β1 and β2 adaptor subunits.

D. Selected but representative TIRFM images of the adherent ventral surface of HeLa SS6 β2-YFP (D) and clone #46β (E) cells. Note the altered morphology of β2-YFP-positive CCSs in the clone #46β cells that closely resembles that seen in HeLa SS6 clone #64/1.E cells, and that is corrected by transient expression of tdRFP-FCHO1 (1-889). Scale bar = 10 μm.

F. Redistribution of endogenous EPS15 in the HeLa clone #46β FCHO2-null β2-YFP expressing line after ectopic expression of Tac-FCHO1 μHD. A typical maximal intensity projection of deconvolved *z* stacks from fixed and permeabilized HeLa clone #46β cells stained with antibodies directed against EPS15 and Tac (transfected cell groups outlined in blue) as indicated. The conspicupous juxtanuclear sequestration of EPS15 (arrows) away from the enlarged clathrin-coated structures at the cell surface is indicated. Scale bar = 10 μm.

Table S1, linked to Figure 3. Data collection and refinement statistics

	**Native EPS15-Fcho1 μHD chimera**	**Xe derivative EPS15-Fcho1 μHD chimera**
**Data Collection**		
Beamline	Diamond IO4-1	Diamond IO2
Space group	H3	H3
Wavelength (Å)	0.92 Å	1.6 Å
**Cell dimensions (Å)**		
a, b, c (Å)	239.8, 239.8, 44.1	236.5, 236.5, 44.0
α, β, γ (°)	90, 90, 120	90, 90, 120
Resolution range (Å)[a]	45.31-2.40 (2.49-2.40)	118.27-2.79 (2.96-2.79)
R_{merge}[ab]	0.80 (1.236)	0.253 (3.80)
R_{meas}[ac]	0.112 (1.745)	0.274 (4.301)
$CC_{1/2}$[ad]	0.996 (0.246)	0.996 (0.243)
Mean $I/\sigma I$[a]	8.3 (1.0)	10.4 (0.8)
Completeness (%)[a]	96.0 (99.1)	99.4 (96.0)
Multiplicity[a]	2.9 (2.9)	13.3 (8.8)
Wilson <B> ($Å^2$)		
Anomolous completeness (%)[a]	83.7 (85.8)	99.3 (95.7)
$CC_{1/2}$ on Δ_{anom} (inner bin)[a]	-0.078 (-0.109)	0.041 (0.257)
**Phasing (autosol)**		
Number of sites		14
Mean figure of merit		0.229
BAYES-CC		38.4
R-factor after density modification		0.29
Map skew after density modification		0.11
**Refinement**		
Resolution range (Å)	43.3-2.4	
No. of reflections	35451	
R_{work}/R_{free}	0.199/0.234	
Number of atoms	4596	
Protein	4335	
Ligand/ion	1	
Waters	40	
B-factors ($Å^2$)	58.4	
**Rmsd from ideal values**		
Bond lengths (Å)	0.01	
Bond angles (°)	0.92	
**Ramachandran Plot**		
Favoured region (%)	98.8	
Allowed (%)	1.2	
Outliers (%)	0	
Rotamer outliers (%)	2	
C-beta outliers	2.89	

[a] Values in parentheses are for the highest resolution shell. [b]$R_{merge} = \Sigma(I_{hl} - \langle I_h \rangle)/\Sigma(I_{hl})$ where $\langle I_h \rangle$ is the mean intensity of unique reflection *h*, summed over all reflections for each observed intensity I_{hl}. [c]$R_{meas} = \Sigma(n/n - 1)^{1/2} (I_{hl} - \langle I_h \rangle)/\Sigma(I_{hl})$ where *n* is the number of observations for unique reflection *h* with mean intensity $\langle I_h \rangle$, summed over all reflections for each observed intensity I_{hl}. [d]$CC_{1/2}$ is the correlation coefficient on $\langle I \rangle$ between random halves of the dataset. Δ_{anom}, anomalous difference $I^+ - I^-$.

Supplemental Experimental Procedures

Materials

Fetal calf serum (FCS) was obtained from Atlanta Biologicals and paraformaldehyde was from Electron Microscopy Sciences. Frozen unstripped rat brain tissue was from PelFreez. Tissue culture medium, trypsin and additives were purchased from Lonza or Mediatech. Alexa Fluor 488, -546 and -647 conjugated transferrin (Tf) was from Invitrogen. Antibiotics, IPTG and DTT were obtained from Gold Biotechnology. Acrylamide, benzamidine, bis-acrylamide, Coomassie blue R250, Tris, Hepes, imidazole, EDTA, EGTA, ammonium persulfate, TEMED, B-PER and Y-PER, LB medium, 2 × YT medium, Lipofectamine 2000, Oligofectamine and a 10 × concentrated PBS stock from Thermo Fisher, benzonase, lysozyme, fatty-acid free BSA, PMSF, reduced glutathione, fish skin gelatin, saponin, Triton X-100 and Ponceau S from Sigma, and cOmplete protease inhibitor tablets from Roche Applied Science. Ni-NTA-agarose was purchased from McLab, Bradford reagent and SDS was from Biorad. Protein molecular mass standards, glutathione-Sepharose and nitrocellulose were purchased from GE Healthcare, and synthetic EPS15 peptides were obtained from Genscript. All DNA primers were synthesized by Integrated DNA Technologies (IDT) and all dideoxynucleotide sequencing was performed by Genewiz using commercial sequencing primers.

Antibodies

Antigen	Species	Designation/ clone	Catalogue number	Supplier/source
β-actin	mouse	mAb C4	sc-47778	Santa Cruz Biotechnology
AP-2 α subunit	mouse	mAb C-8	sc-17771	Santa Cruz Biotechnology
AP-2 α subunit	mouse	mAb AP.6	—	Frances Brodsky
AP-1/2 β1/β2 subunit	rabbit	antigen affinity purified pAb GD/1	—	Traub laboratory
AP-1/2 β1/β2 subunit	mouse	mAb 100/1	—	Ernst Ungewickell
AP-2 μ2 subunit	rabbit	R11-29 serum	—	Juan Bonifacino
CALM	goat	affinity purified pAb C-18	sc-6433	Santa Cruz Biotechnology
CALM/PICALM	rabbit	mAb ab172962	EPR12177	AbCAM
clathrin heavy chain	mouse	mAb TD.1	—	Frances Brodsky
Dab2	rabbit	antigen affinity purified pAb	—	Traub laboratory
Dab2	rabbit	mAb D709T	12906	Cell Signaling Technology
Disabled-2/p96	mouse	mAb 52/p96	610464	BD Transduction Laboratories
Eps15	rabbit	affinity purified pAb C-20	sc-534	Santa Cruz Biotechnology
Eps15	rabbit	antigen affinity purified pAb	—	Ernst Ungewickell
Eps15	rabbit	mAb D3K8R	12460	Cell Signaling Technology
Eps15R	rabbit	mAb ab53006	EP1147Y	AbCAM
Epsin 1	rabbit	antigen affinity purified pAb	—	Traub laboratory
FCHO1	rabbit	antigen affinity	—	Traub laboratory

		purified pAb		
FCHO1	mouse	mAb G-7	sc-365043	Santa Cruz Biotechnology
FCHO2	rabbit	antigen affinity purified pAb	NBP2-32694	Novus Biologicals
GAPDH	mouse	mAb	Y1041	UBP Bio
GFP	rabbit	antigen affinity purified pAb B5	—	Phyllis Hanson
GPP130	rabbit	serum	—	Adam Linstedt
Hrb/RIP/Rab	goat	affinity purified pAb C-19	sc-1424	Santa Cruz Biotechnology
Hrb/AGFG1	mouse	mAb H-2	sc-166651	Santa Cruz Biotechnology
Intersectin 1	mouse	mAb clone 29	611574	BD Transduction Laboratories
PKCα	mouse	mAb MC5	sc-80	Santa Cruz Biotechnology
SHIP2	mouse	mAb E-2	sc-166641	Santa Cruz Biotechnology
Tac (CD25)	mouse	mAb 7G7B6	174-020	Ancell
Tac (CD25)	goat	antigen affinity-purified pAb	AF-223-NA	R & D Systems

Plasmids, DNA manipulations and RNAi oligonucleotides

We used *Homo sapiens* (*Hs*) FCHO1 μHD, *Danio rerio* (*Dr*) Fcho1, *Hs* FCHO2 μHD, *Hs* EPS15 and *Mus musculus* (*Mm*) Sgip1 μHD cDNAs (Reider et al., 2009; Umasankar et al., 2014; Umasankar et al., 2012). GST was produced from the pGEX-4T-1 plasmid and all GST-fusion proteins were constructed in this parental vector. GST-AP-2 α_C appendage (GST-α_C) (Traub et al., 1999) and GST-FCHO1 μHD, GST-FCHO2 μHD, GST-Sgip1 μHD, GST-EPS15 (595-740), (595-660), (595-636), (595-620) and GST-EPS15 (595-896)-His_{12} have been described previously (Umasankar et al., 2014; Umasankar et al., 2012). *Hs* EPS15 residues 615-636 were excised from GST-EPS15 (595-636) plasmid using an internal EcoRI restriction site and re-cloned into the same vector to create GST-EPS15 (615-636). Similarly, GST-EPS15 (626-639) was obtained by annealing appropriate oligonucleotide pairs between EcoRI and XhoI sites in the parent vector. Addition of synthetic GAGA linker in-frame of *Dr* Fcho1 μHD toward the N-terminus yielded GST-GAGA-*Dr* Fcho1 μHD. Subsequent insertion of a *Hs* EPS15 (615-637) amplicon, further upstream, between BamHI and EcoRI sites in the same vector created GST-EPS15-μHD. Construction of GFP-FCHO1 (1-889), GFP-FCHO1 (1-609), Tac-FCHO1 μHD, and Tac-FCHO1 linker have been explained elsewhere (Umasankar et al., 2014; Umasankar et al., 2012). Full-length *Hs* FCHO1 (1-889), *Hs* FCHO1 μHD (609-889) and *Hs* EPS15 (1-896) were PCR amplified and cloned in frame following tdRFP in the ptdTomato-C1 vector. The GFP-FYVE-FCHO1 μHD was cloned into the GFP-(2 × Hrs)FYVE-UbΔGG plasmid from Pietro De Camilli (Chen and De Camilli, 2005) by replacing the C-terminal ubiquitin with an EcoRI-NotI digested FCHO1 μHD PCR product. Routine PCR amplification typically used PfuTurbo DNA polymerase (Agilent Technologies). Two methods for DNA modification were used. Site-directed mutagenesis most often was with the QuikChange system (Invitrogen), utilizing primer pairs (Table S3) that incorporated the necessary mutations mentioned in the text. The Phusion HT (Thermo Fisher) PCR procedure was used to remove or add additional sequences to existing plasmid templates using the appropriate 5'-phosphorylated primer sets. Detailed information on the plasmids, cDNA inserts and cloning primers are provided below:

Gene product/ Protein	Plasmid	Oligo sequence	Restriction sites	Notes
GST-FCHO1 μHD	pGEX-4T-1	5' ATA GAA TTC CAC GGA GTC TCC CGG G 3'<br>5' GCG TCG ACT CAG CAG CTC ACC AGG TAC 3'	EcoRI/SalI	Directional cloning
GST-FCHO2 μHD	pGEX-4T-1	5' GCG GAT CCG GTG TGT CAC GGG GTC CCA GC 3'<br>5' ATG CGG CCG CTC AAC AAT CCG CCA GGT ATC G 3'	EcoRI/NotI	Directional cloning
GST-*Mm* Sgip1 μHD	pGEX-4T-1	5' TCG GAT CCA CTC CCA CAG TTG GTT CC 3'<br>5' AAG CGG CCG CTT AGT TAT CTG CCA AGT AC 3'	BamHI/NotI	Directional cloning
GST-EPS15 (595-740)	pGEX-4T-1	5' GAA GAT CCT TTT CGT TGA GCC ACA TCG AGC TCT G 3'<br>5' CAG AGC TCG ATG TGG CTC AAC GAA AAG GAT CTT C 3'		QuikChange mutagenesis
GST-EPS15 (595-660)	pGEX-4T-1	5' GAC TGT TTC TTC AGG TAA TCT ACT GAT CCT TTT GCC 3'<br>5' GGC AAA AGG ATC AGT AGA TTA CCT GAA GAA ACA GTC 3'		QuikChange mutagenesis
GST-EPS15 (595-636)	pGEX-4T-1	5' CTT TCA AGG ATG ATC CTT TTT GAA AAA TCG ATC CAT TTG GTG G 3'<br>5' CCA CCA AAT GGA TCG ATT TTT CAA AAA GGA TCA TCC TTG AAA G 3'		QuikChange mutagenesis
GST-EPS15 (595-620)	pGEX-4T-1	5' GAT ACA AAC TTG GAT TTT TTC TAG TCT GAT CCT TTT GTT GGC AG 3'<br>5' CTG CCA ACA AAA GGA TCA GAC TAG AAA AAA TCC AAG TTT GTA TC 3'		QuikChange mutagenesis
GST-EPS15 (626-639)	pGEX-4T-1	5' AAT TCG TTG GCA GTG ATC CTT TCA AGG ATG ATC CTT TTG GAA AAA TCC 3'<br>5' TCG AGG ATT TTT CCA AAA GGA TCA TCC TTG AAA GGA TCA CTG CCA ACG 3'	EcoRI/XhoI	Directional cloning
GST-EPS15 (615-636)	pGEX-4T-1	5' GCT GAC AGG TCC AGT TGC AGA ATT CAC AAA CTT GGA TTT TTT CCA G 3'<br>5' CTG GAA AAA ATC CAA GTT TGT GAA TTC TGC AAC TGG ACC TGT CAG C 3'		QuikChange mutagenesis
GST-EPS15 (615-636) [623]DPF→APA	pGEX-4T-1	5' GGA TTT TTT CCA GTC TGC TCC TGC TGT TGG CAG TGA TCC TTT C 3'<br>5' GAA AGG ATC ACT GCC AAC AGC AGG AGC AGA CTG GAA AAA ATC C 3'		QuikChange mutagenesis
GST-*Dr* Fcho1 μHD	pGEX-4T-1	5' GTG AAT TCG GTC TGT CTC GAG GAC CCA G 3'<br>5' ATG CGG CCG CTC ATA AAG AAC AGC CGG CCA TG 3'	EcoRI/NotI	Directional cloning
GST-*Hs* EPS15-*Dr* Fcho1 μHD	pGEX-4T-1	5' TAT GGA TCC ACA AAC TTG GAT TTT TTC CAG TC 3'<br>5' CGT GAA TTC TCC AAA AGG ATC ATC CTT G 3'	BamHI/EcoRI	Directional cloning
GST-*Hs* EPS15-GAGA-*Dr* Fcho1 μHD	pGEX-4T-1	5' TTC AAG GAT GAT CCT TTT GGA GGC GCT GGA GCA GGT CTG TCT CGA GGA CCC AG 3'<br>5' CTG GGT CCT CGA GAC AGA CCT GCT CCA GCG CCT CCA AAA GGA TCA TCC TTG AA 3'		QuikChange mutagenesis
GST-ΔEPS15-μHD	pGEX-4T-1	5'-/5Phos/GTT GGC AGT GAT CCT TTC AAG GAT GAT C 3'<br>5'-/5Phos/GGA TCC ACG CGG AAC CAG ATC 3'		Phusion PCR
GST-EPS15 [APA]-μHD	pGEX-4T-1	5' GGA TTT TTT CCA GTC TGC TCC TGC TGT TGG CAG TGA TCC TTT C 3'<br>5' GAA AGG ATC ACT GCC AAC AGC AGG AGC AGA CTG GAA AAA ATC C 3'		QuikChange mutagenesis
GST-FCHO1 μHD (K877E)	pGEX-4T-1	5' CAT GTC GCT GGT GGA GAG GAG GTT TGC C 3'<br>5' GGC AAA CCT CCT CTC CAC CAG CGA CAT G 3'		QuikChange mutagenesis
GST-FCHO1 μHD (K877E + R879A)	pGEX-4T-1	5' CGC ATG TCG CTG GTG GAG AGG GCG TTT GCC ACA GGG ATG TAC CTG 3'<br>5' CAG GTA CAT CCC TGT GGC AAA CGC CCT CTC CAC CAG CGA CAT GCG 3'		QuikChange mutagenesis
GST-FCHO2 μHD (K798E)	pGEX-4T-1	5' ATA GGC TTT CCT TAA TAG AGA AGC GGT TTG CTA CTG G 3'		QuikChange [illegible]

GST-FCHO2 μHD (K798E)	pGEX-4T-1	5' ATA GGC TTT CCT TAA TAG AGA AGC GGT TTG CTA CTG G 3'<br>5' CCA GTA GCA AAC CGC TTC TCT ATT AAG GAA AGC CTA T 3'		QuikChange mutagenesis
GST-FCHO1 μHD (Y733A)	pGEX-4T-1	5' AAC CCC ACT GCC TCC GCC TAC AAC GTG GTG C 3'<br>5' GCA CCA CGT TGT AGG CGG AGG CAG TGG GGT T 3'		QuikChange mutagenesis
GST-FCHO1 μHD (R872A)	pGEX-4T-1	5' GTG GGC AGC GGT TAC GCC ATG TCG CTG GTG 3'<br>5' CAC CAG CGA CAT GGC GTA ACC GCT GCC CAC 3'		QuikChange mutagenesis
GST-FCHO1 μHD (S874G)	pGEX-4T-1	5' AGC GGT TAC CGC ATG GGG CTG GTG AAG AGG AG 3'<br>5' CTC CTC TTC ACC AGC CCC ATG CGG TAA CCG CT 3'		QuikChange mutagenesis
GST-FCHO1 μHD (R872A+S874G)	pGEX-4T-1	5' GTG GGC AGC GGT TAC GCC ATG GGG CTG GTG AAG AGG AG 3'<br>5' CTC CTC TTC ACC AGC CCC ATG GCG TAA CCG CTG CCC AC 3'		QuikChange mutagenesis
GST-FCHO1 μHD (R872M)	pGEX-4T-1	5' CTG GTG GGC AGC GGT TAC ATG ATG TCG CTG GTG AAG AG 3'<br>5' CTC TTC ACC AGC GAC ATC ATG TAA CCG CTG CCC ACC AG 3'		QuikChange mutagenesis
GST-FCHO1 μHD (S732D)	pGEX-4T-1	5' CAG AAC CCC ACT GCC GAC TAC TAC AAC GTG GTG CTG 3'<br>5' CAG CAC CAC GTT GTA GTA GTC GGC AGT GGG GTT CTG 3'		QuikChange mutagenesis
GST-FCHO1 μHD (N735A + R740A)	pGEX-4T-1	5' GCC TCC TAC TAC GCC GTG GTG CTG CTG GCA TAC CAG TTC TCC CGC 3'<br>5' GCG GGA GAA CTG GTA TGC CAG CAG CAC CAC GGC GTA GTA GGA GGC 3'		QuikChange mutagenesis
GST-FCHO1 μHD (T633Y)	pGEX-4T-1	5' GCC ACA GCC TTC TAT GAG TAT GTC CAC GCC TAC TTC CG 3'<br>5' CGG AAG TAG GCG TGG ACA TAC TCA TAG AAG GCT GTG GC 3'		QuikChange mutagenesis
GST-FCHO1 μHD (Y635S)	pGEX-4T-1	5' GCC TTC ACG GAG TCT GTC CAC GCC TAC TTC CG 3'<br>5' CGG AAG TAG GCG TGG ACA GAC TCC GTG AAG GC 3'		QuikChange mutagenesis
GST-FCHO1 μHD (P627D)	pGEX-4T-1	5′ GGC TCC CAG GAT GCC CTG GAC ATA GCC ACA GCC TTC 3′<br>5′ GAA GGC TGT GGC TAT GTC CAG GGC ATC CTG GGA GCC 3′		QuikChange mutagenesis
GST-FCHO1 μHD (A629E)	pGEX-4T-1	5′ CAG GAT GCC CTG CCC ATA GAG ACA GCC TTC ACG GAG 3′<br>5′ CTC CGT GAA GGC TGT CTC TAT GGG CAG GGC ATC CTG 3′		QuikChange mutagenesis
GST-FCHO1 μHD (P627D + A629E)	pGEX-4T-1	5′ GCC TCC CAG GAT GCC CTG GAC ATA GAG ACA GCC TCC ACG GAG TAT G 3′<br>5′ CAT ACT CCG TGA AGG CTG TCT CTA TGT CCA GGG CAT CCT GGG AGC C 3′		QuikChange mutagenesis
GST-FCHO1 μHD (R872E)	pGEX-4T-1	5′ CTG GTG GGC AGC GGT TAC GAG ATG TCG CTG GTG AAG AGG 3′<br>5′ CCT CTT CAC CAG CGA CAT CTC GTA ACC GCT GCC CAC CAG 3′		QuikChange mutagenesis
GFP-FCHO1 (1-889)	pEGFP-C1	5' ATT GAA TTC TAT GTC GTA TTT TGG CGA GC 3'<br>5' GCG TCG ACT CAG CAG CTC ACC AGG TAC 3'	EcoRI/SalI	Directional cloning
GFP-FCHO1 (1-609)	pEGFP-C1	5' CAG ACA GGA CAC TGA GTC TCC CGG G 3'<br>5' CCC GGG AGA CTC AGT GTC CTG TCT G 3'		QuikChange mutagenesis
tdRFP-FCHO1 (1-889)	ptdTomato-C1	5' ATT GAA TTC TAT GTC GTA TTT TGG CGA GC 3'<br>5' GCG TCG ACT CAG CAG CTC ACC AGG TAC 3'	EcoRI/SalI	Directional cloning
tdRFP-FCHO1 (609-889/μHD)	ptdTomato-C1	5' ATA GAA TTC ACA CGG AGT CTC CCG GG 3'<br>5' GCG TCG ACT CAG CAG CTC ACC AGG TAC 3'	EcoRI/SalI	Directional cloning
tdRFP-EPS15(1-896)	ptdTomato-	5' TTC TCG AGG AAT GGC TGC GGC AGC CC 3'	XhoI/BamHI	Directional

tdRFP-EPS15 (1-896/ Δ618-636)	ptdTomato-C1	5'-/5Phos/CAA GTT TGT ATC TGC AAC TGC ATC TG 3'<br>5'-/5Phos/GGA AAA ATT GAT CCA TTT GGT G 3'		Phusion PCR
tdRFP-EPS15 (1-636)	ptdTomato-C1	5' CCT TTC AAG GAT GAT CCT TTT TGA AAA ATT GAT CCA TTT GGT GG 3'<br>5' CCA CCA AAT GGA TCA ATT TTT CAA AAA GGA TCA TCC TTG AAA GG 3'		QuikChange mutagenesis
tdRFP-EPS15 (1-614)	ptdTomato-C1	5' GAC AGA TGC AGT TGC AGA TTA AAA CTT GGA TTT TTT CCA GTC TGA 3'<br>5' TCA GAC TGG AAA AAA TCC AAG TTT TAA TCT GCA ACT GCA TCT GTC 3'		QuikChange mutagenesis
Tac-FCHO1 μHD	pcDNA3.1	5' ATG ATA TCC ACG GAG TCT CCC GGG G 3'<br>5' TAG CGG CCG CTC AGC AGC TCA CCA GGT ACA TCC 3'	EcoRV/NotI	Directional cloning
Tac-FCHO1 μHD (K877E+R879A)	pcDNA3.1	5' CGC ATG TCG CTG GTG GAG AGG GCG TTT GCC ACA GGG ATG TAC CTG 3'<br>5' CAG GTA CAT CCC TGT GGC AAA CGC CCT CTC CAC CAG CGA CAT GCG 3'		QuikChange mutagenesis
Tac-FCHO1 linker	pcDNA3.1	5' CAG ACA GGA CAC TGA GTC TCC CGG G 3'<br>5' CCC GGG AGA CTC AGT GTC CTG TCT G 3'		QuikChange mutagenesis
GFP-FYVE-FCHO1 μHD	pEGFP-C1	5' ATA GAA TTC CAC GGA GTC TCC CGG G 3'<br>5' TAG CGG CCG CTC AGC AGC TCA CCA GGT ACA TCC 3'	EcoRI/NotI	Directional cloning
Tac-Sgip1 μHD	pcDNA3.1	5' TCG ATA TCG CTG AAA GCA CTT CTT C 3'<br>5' AAG CGG CCG CTT AGT TAT CTG CAA AGT AC 3'	EcoRV/NotI	Directional cloning

For siRNA-mediated silencing of the EPS15- and EPS15R-encoding transcripts, siGENOME human EPS15 (2060; 5'-CCACCAAGAUUUCAUGAUA-3', 5'-GAUCGGAACUCCAACAAGA-3', 5'-AAACGGAGCUACAGAUUAU-3', and 5'-CCACACAGCAUUCUUGUAA-3') and human EPS15L1 (58513; 5'-GAAGUUACCUUGAGCAAUC-3', 5'-GGACUUGGCCGAUCCAGAA-3', 5'-GCACUUGGAUCGAGAUGAG-3', and 5'-CAAAGACCAAUUCGCGUUA-3') SMARTpools were purchased from (Dharmacon/GE Heatlthcare) and were used as described (Teckchandani et al., 2012).

Cell culture and transfections

HeLa SS6 (Elbashir et al., 2001), genome edited FCHO1+FCHO2 functionally-null HeLa SS6 clone #64/1.E (1.E cells) (Umasankar et al., 2014), HeLa SS6 cells stably transfected (Keyel et al., 2008) with a plasmid encoding a YFP-tagged β2 subunit of AP-2 (Sorkina et al., 2002), and a HeLa β2-YFP FCHO2-null gene-edited line designated clone #46β were cultured at 37°C in a 5% CO_2 humidified atmosphere in DMEM supplemented with 10% FCS and 2 mM L-glutamine. The β2-YFP-expressing stable cell medium also contained 0.5 mg/ml G418. The HeLa SS6 clone #46β cells were generated using the parental β2-YFP HeLa SS6 cells and the same TALEN pair (Figure S7A) we used previously to create the HeLa SS6 clone #64 FCHO2-null line. The overall method was identical to that used previously (Umasankar et al., 2014) and the genotype and phenotype of the clone #46β cells is reported in Figure S7B-E.

Preparative batches of HeLa cell lysate were made from HeLa SS6 cells grown to confluence in at least six 150-mm Petri dishes. Cells were detached using Cellstripper (Cellgro/Corning) and washed by centrifugation

in PBS prior to solubilization. To seed cells for transfection, trypsinized cells were plated into 35-mm Petri dishes containing 12-mm #1 round glass cover slips, while sterile 35-mm glass-bottomed, poly-D-lysine-coated MatTek dishes were used for time-resolved live-cell imaging studies. Transfections generally used Lipofectamine 2000 (Thermo Fisher) and endotoxin-free DNA prepared with PureYield midiprep kits (Promega).

For siRNA silencing experiments, adherent HeLa clone 1.E cells were transfected twice with sets of four RNA oligonucleotides targeting either EPS15 or EPS15R transcripts together (200 pmol of each pool) in conjunction with Oligofectamine (ThermoFisher Scientific). After the first-round transfections, cells were trypsinized and replated onto round glass cover slips in 35-mm Petri dishes and analyzed ~40 h after the second-round transfections (Umasankar et al., 2012). Duplicate 35-mm dishes were seeded for preparation of lysates and immunoblotting. Under these conditions, this silencing procedure produces an approximately 50% reduction in steady-state EPS15 levels and approximately 75-80% reduction in EPS15R levels in the whole population.

Protein purification

For pull-down assays, control GST and the required GST-fusion proteins were purified from *E. coli* BL21 codon plus RIL cell pellets by one of two procedures. For the majority of proteins, the frozen pellets were resuspended in a buffer of 50 mM Tris-HCl, pH 7.2, 300 mM sodium chloride, 10 mM β-mercaptoethanol and 0.2% (v/v) Triton X-100 on ice, 1 mM PMSF added, and then subject to three cycles of microprobe sonication on ice with a 1-2 min cooling period between each burst of sonication. Alternatively, bacterial pellets were resuspended at room temperature in a 3:1 mixture of B-PER and Y-PER with 200 μg/ml lysozyme, 0.05 U/ml benzonaze and 1 mM PMSF added. In both cases, the extracts were centrifuged at 24,000 *g* for 20 min and the resulting clarified lysate supernatant transferred to a new tube and gluthathione-Sepharose beads added. After incubation at 4°C for ~2 hours with continuous up-and-down mixing, the beads were recovered by centrifugation at 500 *g* for 5 min and the unbound material discarded. Following extensive washing of the beads with PBS and centrifugation, the GST proteins were eluted with a buffer of 50 mM reduced glutathione and 10 mM DTT. Protein concentrations were determined with the Bradford assay using BSA as a standard. Thrombin-cleaved FCHO1 μHD was prepared from the GST-μHD fusion by overnight incubation with thrombin at room temperature. For the monomeric EPS15 (595-896), a C-terminally His_{12}-tagged GST-fusion form was first collected on glutathione-Sepharose, washed and treated with thrombin overnight. The thrombin-released EPS15 was then purified over a Ni-NTA-agarose column and eluted with 50 mM Tris-HCl, pH 8.0, 300 mM sodium chloride and 500 mM imidazole.

Proteins used in structure determination and ITC were similarly expressed as GST fusion proteins in the *E. coli* BL21(DE3) plysS strain at 22°C for 16 hours. Cells were lysed and insoluble material removed by centrifugation. GST fusion proteins were bound to glutathione-Sepharose in buffer A (20 mM Tris-HCl, pH 7.4, 500 mM sodium chloride) with 0.1 mM ABESF, washed extensively with 100 volumes of buffer A and the

proteins cleaved from GST by overnight incubation at 22°C with thrombin. Proteins were concentrated to ~15 mg/ml and further purified over a HiLoad 26/600 Superdex S200 gel filtration column (GE Healthcare) equilibrated in buffer A with 0.1 mM ABESF and 2 mM DTT. Any remaining GST or uncleaved fusion proteins were removed by passage through a 1 ml glutathione-Sepharose column. For crystallization, protein was gel filtered into in buffer A with ABESF and for ITC into 100 mM Tris-HCl pH 7.5, 200 mM sodium chloride and 0.25 mM TCEP. After first attempting crystallization of μHDs from human, *Xenopus* or zebrafish (*D. rerio*) Fcho1/2, both alone and together with synthetic EPS15 peptides, we switched to in-frame chimeras of human EPS15 (615-637) with either the human FCHO1 or *D. rerio* Fcho1 μHD, only the latter of which crystallized. The best crystals of the EPS15-μHD chimeric protein (15 mg/ml) grew over a period of 2-3 weeks by hanging drop vapor diffusion against a reservoir containing 100 mM Bis-Tris propane pH, 6.0, 200 mM sodium citrate, 22% (v/v) PEG 3350 and 10 mM DTT. For native and xenon derivatives, crystals were cryoprotected in mother liquor fortified with 22% (v/v) glycerol and flash frozen. Xe derivatives were made by pressurizing crystals to between 10 and 12 atmospheres of pressure in a homemade Xe chamber. Crystals were of the space group H3 with two molecules in the asymmetric unit (a=b=239.78 c=44.09, $\alpha=\beta=90.00$ $\gamma=120.00$) and diffracted at best to 2.4 Å at Diamond Light Source synchrotron. Data were collected using an inverse beam strategy with 5° wedges. The structure was solved by single-wavelength anomalous dispersion (SAD) using AutoSol (Terwilliger et al., 2009). Initial building was accomplished with ten cycles of Buccaneer (Cowtan, 2006) and refined using alternating cycles of Phenix.refine (Adams et al., 2010; Afonine et al., 2012) and manual rebuilding in COOT (Emsley et al., 2010). The final model contains 594 residues, 40 water molecules and one Zn^{2+} atom, and was refined to final R/Rfree of 0.199/0.234 (Table S1). The concave face of the Fcho1 μHD is overall considerably more curved than in all other μHDs that were used as models to attempt to solve the structure by molecular replacement, explaining the lack of success using this technique. In addition, the two molecules in the asymmetric unit interact with a metal ion (Zn^{2+} on the basis of weak anomalous scattering) coordinated by H1031 and C1033 in each μHD. These residues are not conserved in Fchos of other species, so we do not consider dimerization biologically relevant.

Rat brain cytosol was prepared in a homogenization buffer of 25 mM Hepes-KOH, pH 7.2, 250 mM sucrose, 2 mM EDTA and 2 mM EGTA. Rapidly thawed frozen brain tissue was homogenized in a Waring Blendor at 4°C in the presence of homogenization buffer with 2 mM PMSF, 5 mM benzamidine and cOmplete protease inhibitor tablets. The resulting thick homogenate was centrifuged at 15,000 *g* for 20 min and the postnuclear supernatant fraction recentrifuged at 17,500 *g* for 20 min. The supernatant was then centrifuged at 105,000 *g* for 60 min. The resulting high-speed supernatant (cytosol) was stored in small frozen aliquots at -80°C. Before use in pull-down assays, the brain cytosol was adjusted to 1 × assay buffer using a 10 × stock solution on ice. HeLa cell Triton X-100 lysates were prepared by first collecting confluent cells from Petri dishes using Cellstripper and centrifuging at 500 *g* for 5 min. The cell pellet was resuspended in cold 25 mM Hepes-KOH, pH 7.2, 125 mM potassium acetate, 5 mM magnesium acetate, 2 mM EDTA, 2 mM EGTA, 2 mM DTT and 1% Triton X-100 and incubated on ice for 30 min with occasional mixing. After centrifugation at 24,000 *g* for 20 min to remove insoluble material, the supernatant (lysate) was stored in frozen aliquots at -

80°C. Thawed cytosol/lysate samples for assays were centrifuged at 125,000 *g* for 20 min at 4°C immediately before use.

GST pull-down assays

The standard format for biochemical assays entails first immobilizing a measured amount of GST or GST-fusion protein onto a standard volume of glutathione-Sepharose beads. In microfuge tubes, the appropriate protein mass was diluted into PBS on ice up to a final volume of 750 μl and then 60 μl of a cold 50% slurry of glutathione-Sepharose beads in PBS added to each tube. After incubation at 4°C with continuous up-and-down mixing, the Sepharose beads were recovered by centrifugation (10,000 *g*, 1 min) and the supernatants aspirated and discarded. Each bead pellet was washed twice with cold assay buffer composed of 25 mM Hepes-KOH, pH 7.2, 125 mM potassium acetate, 5 mM magnesium acetate, 2 mM EDTA, 2 mM EGTA and 2 mM DTT. After the second spin, the majority of the buffer was carefully aspirated, leaving a final equivalent volume of ~50 μl in each tube. Aliquots of rat brain cytosol or HeLa cell Triton X-100 lysate were thawed from -80°C and centrifuged at 125,000 *g* for 20 minutes at 4°C to remove insoluble particulate matter before addition to the tubes of immobilized GST or GST-fusion protein. Typically 200-250 μl of cytosol/lysate was added, the beads resuspended, and then incubated at 4°C for 60 min with continual up-and-down mixing. Binding assays were terminated by centrifugation (10,000 *g*, 1 min, 4°C) and an aliquot (typically 60 μl) removed and transferred to a new microfuge tube. At this stage, the incubations are still at steady state, so comparison of the differences in binding partner protein recovery in the various supernatant fractions presented in the figures *versus* the control GST can be used to evaluate the extent of binding to the relevant GST-fusion protein being tested. The pellets were then washed by centrifugation four times with ~1.5 ml/wash ice-cold PBS and the supernatants aspirated and discarded. The Sepharose beads were resuspended in reducing SDS sample buffer, and the volumes adjusted manually so that all tubes were equivalent. Importantly, during washing, the reactions are no longer at steady state due to repeated dilution, and so comparison of the pellets may give the impression that a given protein bound differentially to various immobilized GST-fused mutants. This is why we present both supernatant and pellet fractions; it allows assessment of whether a particular mutation, or set of mutations, alters either the on-rate (k_{on}), the off-rate (k_{off}), or both. For example in comparing the GST-*Hs* FCHO1 μHD mutants, the N735A+R740A protein is less compromised than a K877E+R879A μHD because less EPS15 and intersectin 1 and no EPS15R remain in the supernatant fractions, and a bound pool of these proteins is evident after washing. After boiling the pull-down samples and centrifugation (12,000 *g*, 1 min), aliquots were fractionated by SDS-PAGE. In all of our pull-down experiments, because of the differences in volumes, we routinely load onto each gel 5 × more of each pellet fraction than of the corresponding supernatant. This accounts for the intensity of the pellet signal often being substantially higher than the control (GST) supernatant fraction.

Isothermal titration microcalorimetry (ITC)

Purified μHDs were gel filtered into 100 mM Tris-HCl pH 7.5, 200 mM sodium chloride and 0.25 mM TCEP. Synthetic peptides (EPS15 residues 615-637 and truncations of thereof) (Genscript) were dissolved in the

same buffer. Experiments were performed using a Nano ITC from TA Instruments. The *Hs* FCHO1 μHD at 0.11 mM was placed in the cell at 12°C and peptides at concentrations between 1.6 and 1.7 μM depending on peptide were titrated in with 19 injections of 2 μl with injections separated by 5 minutes. Experiments with the zebrafish Fcho1 μHD were carried out with 0.3 μM protein in the cell and were carried out at 4°C due to the lower thermal stability of this protein. In addition, the *Hs* FCHO1 μHD mutants in the area of the α-helical portion of subdomain A were labile and prone to aggregation when assayed at 12°C. Therefore, the P627D, A629E and P627D+A629E mutants were similarly tested with the reaction cell equilibrated to 4°C. An appropriate buffer into protein blank was subtracted from all data runs and for constructs which displayed measurable binding, a minimum of four independent runs that showed clear saturation of binding were used to calculate the mean K_D of the reaction, its stoichiometry (n) and their corresponding SEM values. Analysis of results and final figure preparation were carried out using the NanoAnalyze™ software.

SDS-PAGE and immunoblotting

Discontinuous polyacrylamide gels were cast manually using an acrylamide-bis-acrylamide stock solution that differs from the typical 30:0.8 ratio by having half the bis-acrylamide (30:0.4). The utility of this altered ratio of crosslinker is that it markedly improves the separation of closely-spaced bands. The standard denaturing Laemmli buffer system was used with reducing sample buffer. After electrophoresis using constant voltage, gels were either stained in Coomassie Blue in 40% methanol, 10% acetic acid or transferred to nitrocellulose. For blotting, the gels were first equilibrated for ~5-10 min in ice-cold transfer buffer of 15.6 mM Tris, 120 mM glycine to reduce the amount of SDS, and then the gel assembled upon a sheet of wet nitrocellulose on moistened filter paper and transferred in ice-cold transfer buffer at a constant 110 V for 75 min. Proteins were visualized using 0.2% Ponceau S in acetic acid, the position of the molecular mass standards marked in pencil, cut into appropriate numbered sections horizontally with a scalpel blade, and then the nitrocellulose destained completely in transfer buffer at room temperature. Non-specific binding sites on the blots were quenched at room temperature with 5% non-fat dried milk in a blotting buffer of 10 mM Tris-HCl, pH 7.8, 150 mM sodium chloride and 0.1% Tween 20. The relevant portions of the blots were then incubated for 1-2 hours at room temperature with primary antibodies diluted in 1% milk in the blotting buffer, washed in blotting buffer and then incubated in 1:5,000 dilutions of anti-mouse/rabbit/goat IgG coupled to horseradish peroxidase for 1 hour at room temperature. Following extensive washing in blotting buffer, the location of bound antibodies was detected with an enhanced chemiluminescence-type reagent (HyGlo, Denville Scientific) and standard X-ray film.

Immunofluorescence staining

We used two different protocols for labeling cells attached to glass coverslips with appropriate antibodies. The first is a standard procedure using paraformadehyde fixation, Triton X-100 permeabilization and quenching in a buffer with 5% normal goat serum in PBS (Keyel et al., 2006; Umasankar et al., 2012). The modified method generally provides superior results with many, but not all, antibodies. In this procedure, cells were first washed twice with PBS and then fixed for 30 min at room temperature in 4% freshly-prepared

paraformaldehyde in PBS adjusted to pH 8.0. The fixed cells were then washed 2 × 5 min with PBS and subsequently incubated in a quenching solution of 75 mM ammonium chloride and 20 mM glycine dissolved in the pH 8.0 PBS. Next, the cells were permeabilized with 0.1% Triton X-100 in PBS, pH 8.0 at room temperature for 30 min. After washing the cells 3 x 5 min with PBS, the cells were blocked for 10 min at 37°C with a mixture of 5% (v/v) normal goat serum in 0.66% fish skin gelatin, 0.05% saponin in pH 8.0 PBS (buffer IF). Primary antibodies were added after dilution in buffer IF and centrifugation at >12,000 *g* for 5 min to remove aggregated material. Incubation was at 37°C for 60 min, followed by three quick rinses with buffer IF and then 3 × 5 min washes using the same buffer. Secondary antibodies were prepared and used in the same manner as the primary antibodies. The cells were rinsed quickly 3 × with buffer IF followed by 3 × 5 min washes in buffer IF, and then 3 x 5 min washes with PBS. A solution of 0.1% Triton X-100 in PBS was added next, for 5 min, then a 5 min wash in PBS and then a post-fixation step with 4% paraformaldehyde in 100 mM sodium cacodylate, pH 7.4 at room temperature for 30 min. The samples were again incubated in quenching solution for 10 min, washed 2 × 5 min with PBS, pH 8.0, and finally mounted on glass slides in Cytoseal.

Microscopy

Confocal imaging of fixed and fluorescently stained samples was performed on an inverted Olympus FV1000 microscope equipped with a PlanApo N (60 × /1.42 numerical aperture) oil objective. Appropriate excitation and emission wavelengths were preprogrammed by the instrument running the FV10-ASW software, and emission signals in the different channels were always collected in the sequential scan mode. Images were exported in TIFF file format and imported into Adobe Photoshop CS4 for minor adjustments to brightness and/or contrast. Quantitation of transferrin uptake in confocal image files was performed using the linescan application within Metamorph (Molecular Devices). The extent of transferrin internalization in confocal image stacks that were collected following the pulse-chase format (as in Fig. ure 5) was analyzed by using Fiji software (Schindelin et al., 2012) . Images were corrected for the background. A 10.09 μm × 10.09 μm area was selected as a region of interest (ROI) in basal, middle and upper sections of each cell in the *z* stack. Each optical section was saved as a separate image and processed separately. A mask for each image was created by applying a manual threshold. The mask was then used on the corresponding ROI image to measure the number and intensity of each spot in different cells. For cells in the different populations (control untransfected, Tac-μHD or Tac-linker expressing), the intensity values of spots in each population set were pooled together and plotted in the histogram. For the different populations, the same total area, 2,443.4 μm^2, was used for the comparative data presented in Figure S6.

TIRFM image acquisition

Cultured HeLa clone #46β cells in glass-bottomed MatTek dishes were examined using TIRFM optics at 37°C with a Nikon TI microscope equipped with both confocal (A1R spectral), wide field, and through-the-objective type TIRFM capabilities. The camera used for all TIRFM and widefield imaging experiments on the Nikon TI microscope was an Andor Zyla 5.5 at full frame with no binning, the laser launch is a 4 color (405

nm, 488 nm, 561 nm and 647 nm lines). All images were collected using NIS Elements software. The appropriately treated HeLa cell clones in MatTek dishes were mounted in a temperature and CO_2 controlled humidified chamber (Tokai Hit, Tokyo Japan) in a Mad City Labs Piezo controlled *z* stage. Using a drift correction device (Perfect Focus, Nikon Instruments) cells were brought into focus and the *z* position locked. Individual fields were selected blindly and then sequential images collected using conditions that caused minimal signal loss due to bleaching with the illuminating laser. Laser conditions varied with experiments, either YFP alone (488 nm excitation 525/40 nm emission filter) or with the 561 nm (600/50 emission filter) or 647 nm (700/75 emission filter). To ensure perfect image registration between colors, a single 4 color TIRFM filter cube (Chroma tech, Brattleboro VT) was used with a high speed (20 ms change time) filter wheel (FLI, NY) to block bleed through between channels.

Rapid-freeze deep-etch electron microscopy

Cells were cultured on small oriented pieced of glass coverslip and ruptured with a brief burst of sonication at room temperature. The formaldehyde fixed adherent cell membrane preparations were immunogold labeled with rabbit anti-FCHO2 antibodies followed by colloidal-gold labeled anti-rabbit IgG, as we have described previously (Edeling et al., 2006; Hawryluk et al., 2006). A detailed discussion of the technique, procedures and possible pitfalls of generating immunogold-labeled platinum replicas of cell cortices is available (Heuser, 2000).

Supplemental References

Adams, P.D., Afonine, P.V., Bunkoczi, G., Chen, V.B., Davis, I.W., Echols, N., Headd, J.J., Hung, L.W., Kapral, G.J., Grosse-Kunstleve, R.W., *et al.* (2010). PHENIX: a comprehensive Python-based system for macromolecular structure solution. Acta Crystallogr D Biol Crystallogr *66*, 213-221.

Afonine, P.V., Grosse-Kunstleve, R.W., Echols, N., Headd, J.J., Moriarty, N.W., Mustyakimov, M., Terwilliger, T.C., Urzhumtsev, A., Zwart, P.H., and Adams, P.D. (2012). Towards automated crystallographic structure refinement with phenix.refine. Acta Crystallogr D Biol Crystallogr *68*, 352-367.

Burgos, P.V., Mardones, G.A., Rojas, A.L., daSilva, L.L., Prabhu, Y., Hurley, J.H., and Bonifacino, J.S. (2010). Sorting of the Alzheimer's disease amyloid precursor protein mediated by the AP-4 complex. Dev Cell *18*, 425-436.

Chen, H., and De Camilli, P. (2005). The association of epsin with ubiquitinated cargo along the endocytic pathway is negatively regulated by its interaction with clathrin. Proc Natl Acad Sci U S A *102*, 2766-2771.

Cowtan, K. (2006). The Buccaneer software for automated model building. 1. Tracing protein chains. Acta Crystallogr D Biol Crystallogr *62*, 1002-1011.

Edeling, M.A., Mishra, S.K., Keyel, P.A., Steinhauser, A.L., Collins, B.M., Roth, R., Heuser, J.E., Owen, D.J., and Traub, L.M. (2006). Molecular switches involving the AP-2 β2 appendage regulate endocytic cargo selection and clathrin coat assembly. Dev Cell *10*, 329-342.

Elbashir, S.M., Harborth, J., Lendeckel, W., Yalcin, A., Weber, K., and Tuschl, T. (2001). Duplexes of 21-nucleotide RNAs mediate RNA interference in cultured mammalian cells. Nature *411*, 494-498.

Emsley, P., Lohkamp, B., Scott, W.G., and Cowtan, K. (2010). Features and development of Coot. Acta Crystallogr D Biol Crystallogr *66*, 486-501.

Hawryluk, M.J., Keyel, P.A., Mishra, S.K., Watkins, S.C., Heuser, J.E., and Traub, L.M. (2006). Epsin 1 is a polyubiquitin-selective clathrin-associated sorting protein. Traffic *7*, 262-281.

Hein, M.Y., Hubner, N.C., Poser, I., Cox, J., Nagaraj, N., Toyoda, Y., Gak, I., Weisswange, I., Mansfeld, J., Buchholz, F., *et al.* (2015). A human interactome in three quantitative dimensions organized by stoichiometries and abundances. Cell *163*, 712–723.

Heuser, J. (2000). The production of 'cell cortices' for light and electron microscopy. Traffic *1*, 545-552.

Keyel, P.A., Mishra, S.K., Roth, R., Heuser, J.E., Watkins, S.C., and Traub, L.M. (2006). A single common portal for clathrin-mediated endocytosis of distinct cargo governed by cargo-selective adaptors. Mol Biol Cell *17*, 4300-4317.

Keyel, P.A., Thieman, J.R., Roth, R., Erkan, E., Everett, E.T., Watkins, S.C., and Traub, L.M. (2008). The AP-2 adaptor β2 appendage scaffolds alternate cargo endocytosis. Mol Biol Cell *19*, 5309-5326.

Owen, D.J., and Evans, P.R. (1998). A structural explanation for the recognition of tyrosine-based endocytotic signals. Science *282*, 1327-1332.

Reider, A., Barker, S.L., Mishra, S.K., Im, Y.J., Maldonado-Baez, L., Hurley, J.H., Traub, L.M., and Wendland, B. (2009). Syp1 is a conserved endocytic adaptor that contains domains involved in cargo selection and membrane tubulation. EMBO J *28*, 3103-3016.

Schindelin, J., Arganda-Carreras, I., Frise, E., Kaynig, V., Longair, M., Pietzsch, T., Preibisch, S., Rueden, C., Saalfeld, S., Schmid, B., *et al.* (2012). Fiji: an open-source platform for biological-image analysis. Nat Methods *9*, 676-682.

Sorkina, T., Huang, F., Beguinot, L., and Sorkin, A. (2002). Effect of tyrosine kinase inhibitors on clathrin-coated pit recruitment and internalization of epidermal growth factor receptor. J Biol Chem *277*, 27433-27441.

Teckchandani, A., Mulkearns, E.E., Randolph, T.W., Toida, N., and Cooper, J.A. (2012). The clathrin adaptor Dab2 recruits EH domain scaffold proteins to regulate integrin b1 endocytosis. Mol Biol Cell *23*, 2905-2916.

Terwilliger, T.C., Adams, P.D., Read, R.J., McCoy, A.J., Moriarty, N.W., Grosse-Kunstleve, R.W., Afonine, P.V., Zwart, P.H., and Hung, L.W. (2009). Decision-making in structure solution using Bayesian estimates of map quality: the PHENIX AutoSol wizard. Acta Crystallogr D Biol Crystallogr *65*, 582-601.

Traub, L.M., Downs, M.A., Westrich, J.L., and Fremont, D.H. (1999). Crystal structure of the α appendage of AP-2 reveals a recruitment platform for clathrin-coat assembly. Proc Natl Acad Sci USA *96*, 8907-8912.

Umasankar, P.K., Ma, L., Thieman, J.R., Jha, A., Doray, B., Watkins, S.C., and Traub, L.M. (2014). A clathrin coat assembly role for the muniscin protein central linker revealed by TALEN-mediated gene editing. eLife *3*, e04137.

Umasankar, P.K., Sanker, S., Thieman, J.R., Chakraborty, S., Wendland, B., Tsang, M., and Traub, L.M. (2012). Distinct and separable activities of the endocytic clathrin-coat components Fcho1/2 and AP-2 in developmental patterning. Nat Cell Biol *14*, 488-501.
